# Triaminopyrimidine is a fast-killing and long-acting antimalarial clinical candidate

**DOI:** 10.1038/ncomms7715

**Published:** 2015-03-31

**Authors:** Shahul Hameed P., Suresh Solapure, Vikas Patil, Philipp P. Henrich, Pamela A. Magistrado, Sowmya Bharath, Kannan Murugan, Pavithra Viswanath, Jayashree Puttur, Abhishek Srivastava, Eknath Bellale, Vijender Panduga, Gajanan Shanbag, Disha Awasthy, Sudhir Landge, Sapna Morayya, Krishna Koushik, Ramanatha Saralaya, Anandkumar Raichurkar, Nikhil Rautela, Nilanjana Roy Choudhury, Anisha Ambady, Radha Nandishaiah, Jitendar Reddy, K. R. Prabhakar, Sreenivasaiah Menasinakai, Suresh Rudrapatna, Monalisa Chatterji, María Belén Jiménez-Díaz, María Santos Martínez, Laura María Sanz, Olivia Coburn-Flynn, David A. Fidock, Amanda K. Lukens, Dyann F. Wirth, Balachandra Bandodkar, Kakoli Mukherjee, Robert E. McLaughlin, David Waterson, Lyn Rosenbrier-Ribeiro, Kevin Hickling, V. Balasubramanian, Peter Warner, Vinayak Hosagrahara, Adam Dudley, Pravin S. Iyer, Shridhar Narayanan, Stefan Kavanagh, Vasan K. Sambandamurthy

**Affiliations:** 1 Department of Innovative Medicines, AstraZeneca India Pvt. Ltd., Bellary Road, Hebbal, Bangalore 560024, India; 2 Department of Microbiology and Immunology, Columbia University Medical Center, New York, New York 10032, USA; 3 Harvard School of Public Health, 665 Huntington Avenue, Boston, Massachusetts 02115, USA; 4 Tres Cantos Medicines Development Campus. Diseases of Developing World (DDW), GlaxoSmithKline, Severo Ochoa 2, Tres Cantos, Madrid 28760, Spain; 5 Division of Infectious Diseases, Department of Medicine, Columbia University Medical Center, New York, New York 10032, USA; 6 AstraZeneca Infection Innovative Medicines, 35 Gatehouse Drive, Waltham, Massachusetts 02451, USA; 7 Medicines for Malaria Venture, International Center Cointrin, Geneva 1215, Switzerland; 8 AstraZeneca, Alderley Park, Cheshire SK10 4TF, UK

## Abstract

The widespread emergence of *Plasmodium falciparum* (*Pf*) strains resistant to frontline agents has fuelled the search for fast-acting agents with novel mechanism of action. Here, we report the discovery and optimization of novel antimalarial compounds, the triaminopyrimidines (TAPs), which emerged from a phenotypic screen against the blood stages of *Pf*. The clinical candidate (compound **12**) is efficacious in a mouse model of *Pf* malaria with an ED_99_ <30 mg kg^−1^ and displays good *in vivo* safety margins in guinea pigs and rats. With a predicted half-life of 36 h in humans, a single dose of 260 mg might be sufficient to maintain therapeutic blood concentration for 4–5 days. Whole-genome sequencing of resistant mutants implicates the vacuolar ATP synthase as a genetic determinant of resistance to TAPs. Our studies highlight the potential of TAPs for single-dose treatment of *Pf* malaria in combination with other agents in clinical development.

## Abstract (web summary)

The emergence of resistant *Plasmodium* strains fuels the search for new antimalarials. Here, the authors present a new class of potent antimalarial compounds, the triaminopyrimidines, that display low toxicity and long half-life in animal models.

## Graphical abstract

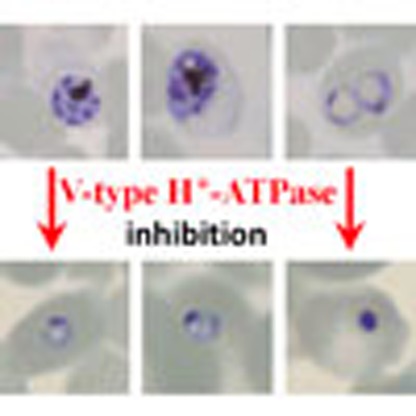

Malaria accounted for an estimated 584,000 deaths in 2013, especially among children and pregnant women^1^. The widespread emergence and dissemination of *Plasmodium falciparum* (*Pf*) strains resistant to conventional antimalarial drugs has intensified the efforts to discover and develop novel, structurally diverse drugs against multidrug-resistant *Plasmodium* strains. This situation is further aggravated by the emergence of *Pf* strains resistant to artemisinin derivatives in parts of South East Asia^2^,^3^,^4^. Even more worrisome is the emergence of *Pf* strains resistant to most available antimalarial medicines in areas along the Cambodia–Thailand border^1^.

A global strategy aimed at administering a combination of novel agents that target the asexual and sexual forms of the parasite is likely to prevent the emergence of resistance. The goal of single-exposure radical cure and prophylaxis can be accomplished through suitable combination of partner drugs with fast-killing and/or long half-life attributes^5^. The advent of high-throughput whole-cell screening against *Pf* has led to the progression of several novel antimalarial agents into preclinical and clinical development^6^,^7^,^8^,^9^,^10^.

The present study describes the discovery and optimization of a novel antimalarial series belonging to the triaminopyrimidines (TAPs) class with the potential for long half-life in humans and displaying potent activity against a panel of clinical strains harbouring resistance to known antimalarial drugs as well as agents in clinical development. The TAPs kill *Pf* parasites rapidly, and the emergence of spontaneous resistance under *in vitro* conditions to this chemical class is rare. We present extensive pharmacokinetic (PK) and preclinical safety data that supports the progression of this novel antimalarial agent towards clinical development to treat malaria.

## Results

### Optimization of TAPs as potent antimalarial agents

**Figure 1:**
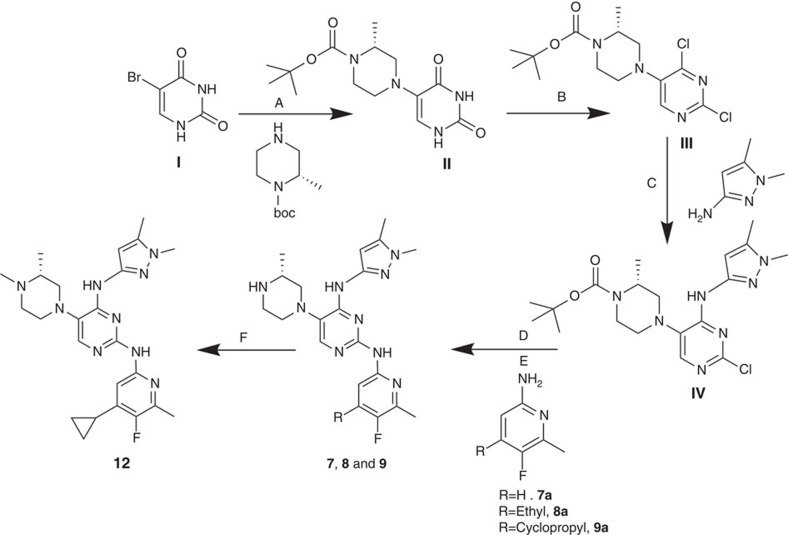
Synthetic schemes and reaction conditions for compounds 7, 8, 9 and 12. (A) Pyridine, microwave, 150 °C, 45 min. (B) (i) POCl_3_, reflux, 6 h (ii) sodium carbonate, di-tert-butyl dicarbonate, room temperature, 16 h. (C) N,N-Diisopropylethylamine (DIPEA), ethanol, microwave, 110 °C, 1 h. (D) (i) Potassium tert-butoxide, 2,2′-bis(diphenylphosphino)-1,1'-binaphthyl (BINAP), pd_2_(dba)_3_, toluene, reflux, 12 h. (E) HCl (4 N) in dioxane, 15–30 min. (F) Compound **9**, DIPEA, dichloromethane, formaldehyde (HCHO), sodium cyanoborohydride, 15 min.

**Figure 2:**
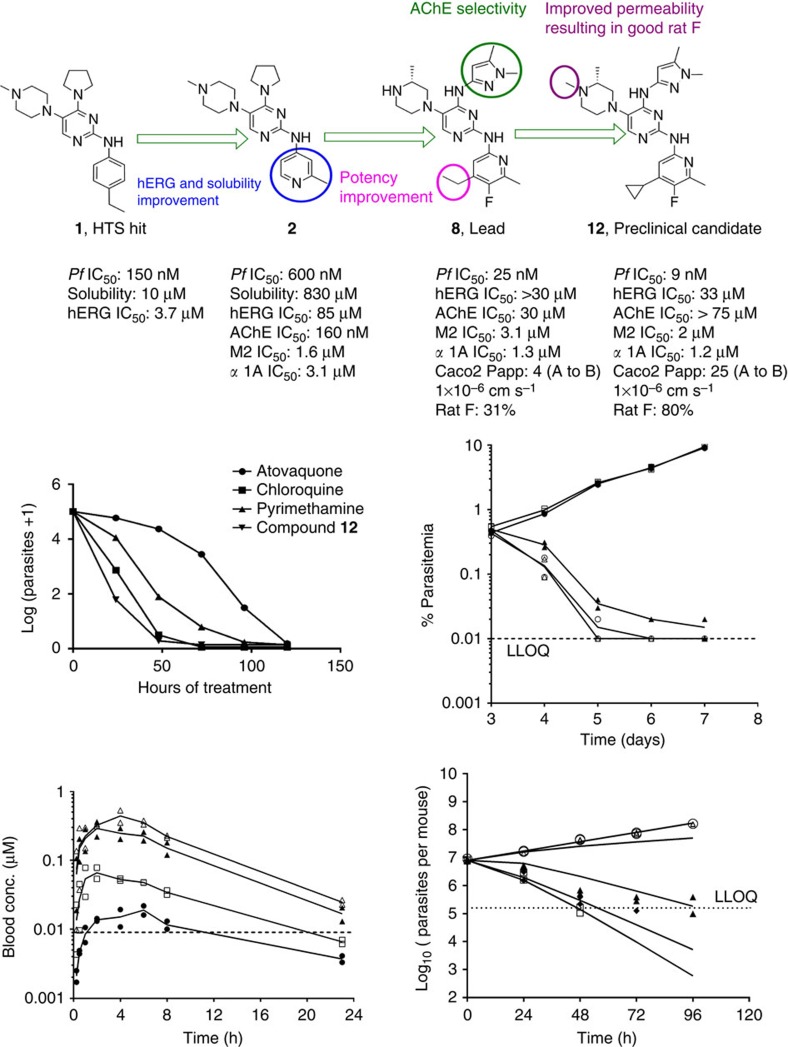
Evolution of clinical candidate compound 12 and its *in vitro* and *in vivo* killing kinetics profile. (**a**) Schematic of medicinal chemistry optimization and identification of clinical candidate **12**. (**b**) *In vitro* parasite reduction ratio (PRR) for compound **12**. The graph shows change in the number of viable parasites over time after exposure of *Pf* 3D7A to atovaquone ([cirf ]), chloroquine (▪), pyrimethamine (▴) or compound 12 (▾) at a concentration (conc.) equal to 10 times their respective IC_50_s. (**c**) Percentage parasitemia in peripheral blood of mice infected with *Pf*3D7^0087/N9^ (*n*=2 per group) after treatment with compound **12** at 10 (□), 20 (▴), 40 (○) or 80 (Δ) mg kg^−1^ or with vehicle (▾). Dotted line indicates the lower limit of quantification (LLOQ) for % parasitemia estimation. (**d**) Blood concentration versus time profile for compound **12** in infected mice is depicted after the first dose of 10 ([cirf ]), 20 (□), 40 (▴) or 80 (Δ) mg kg^−1^. Dotted line indicates the *Pf* IC_50_. Two mice were used per dose group. (**e**) Predicted (lines) and observed (symbols) change in the total parasite burden in infected mice in the *Pf*/SCID model after treatment with compound **12** at 10 (Δ), 20 (▴), 40 (♦) or 80 (□) mg kg^−1^ or with vehicle (○). SCID, severe combined immunodeficient.

The TAPs series emerged from a high-throughput screen (HTS) of 500,000 compounds from the AstraZeneca corporate library against the asexual blood stage of *Pf* using a high-content image-based approach^10^. The synthetic scheme for representative compounds (**7**, **8**, **9** and **12**) is shown in Fig. 1. The procedure for synthesis of compounds **1-6, 10, 11** using a scheme similar to Fig. 1 is provided in Supplementary Note 1. The initial hit provided multiple diversification points for a robust lead optimization campaign. A schematic of the medicinal chemistry optimization and structure activity relationship (SAR) of TAPs are shown in Fig. 2a and Table 1. The SAR analysis revealed essentiality of the basic ring substitution at the C-5 position of the pyrimidine core for *Pf* potency. The initial hit (compound **1**) exhibited cardiac ion channel inhibition (hERG, IC_50_–3.7 μM) and poor solubility (10 μM). Replacement of the phenyl ring with a 4-pyridyl ring system at the 2-amino position improved solubility (830 μM) and reduced the hERG liability (IC_50_ 85 μM). Compounds with 2-pyridyl modification at the 2-amino position of pyrimidine core improved potency and solubility at the cost of potent activity against hERG and acetylcholine esterase (AChE; compounds **3-6**; Table 1). Compound **7**, with a 2-aminopyrazole modification reduced the potency against hERG (IC_50_ >33 μM) and AChE (IC_50_–66 μM) with a concomitant, four-fold loss in *Pf* potency. The observed hERG and AChE selectivity improvement with **7** could be attributed to the polar nature of the pyrazole group and its role in reducing the pKa of the core pyrimidine ring. The introduction of a cyclopropyl group at the 4-position of pyridine in **7** resulted in compound **9** with a >10-fold improvement in *Pf* potency with an excellent hERG and AChE selectivity, albeit with suboptimal bioavailability (Table 1). Compound **12** with *N*-methylpiperazine at the C-5 position (*N*-methyl analogue of **9**) showed the best potency (9 nM) combined with a >1,000-fold selectivity against hERG and AChE (Table 1). In addition, **12** displayed improved bioavailability (80%) as compared with its demethylated analogue, **9** owing to its improved Caco2 permeability (Fig. 2a). Compound **12** exhibiting desirable PK properties was evaluated for *in vivo* efficacy and safety. To assess the potential for cross-resistance, representative TAPs were screened against a panel of clinical isolates and *in vitro*-generated mutant strains displaying varying mechanisms of resistance to antimalarial agents currently in use and under development, respectively, (Supplementary Tables 1–3). TAPs retained their IC_50_ against these strains, thereby suggesting a novel mechanism of action.

TAPs lack appreciable activity against the sexual forms or liver stages of the parasite (IC_50_ >1 μM). TAPs were found to be inactive in the *P. berghei* liver schizont assay (IC_50_ >10μM) as well as the *P. cynomolgi* liver schizont and hypnozoite assay (IC_50_ >10 μM). Compound **12** was tested for its *in vitro* activity in the *Pf* male and female dual gamete assay and was found to be inactive (16.5±6.3% inhibition at 1 μM).

### Pharmacokinetics-pharmacodynamics (PK-PD) of TAPs

Faster reduction in the blood parasite burden is essential to provide quick relief from clinical symptoms and to minimize the risk of emergence of drug resistance^5^. Compound **12** produced a >4-log kill after 48 h of exposure in the *in vitro* parasite reduction ratio (PRR) assay^11^, an effect similar to chloroquine (Fig. 2b).

In the *Pf*/SCID model^12^, **12** cleared *Pf* parasites to below detection limit following 4 days of daily treatment with 20 mg kg^−1^ dose administered through the oral route. A maximum kill rate was observed at 40 mg kg^−1^ (Fig. 2c). Blood *C*_min_ (0.04 μM) observed at this dose (Fig. 2d) was considered as the minimum parasiticidal concentration (MPC) for the human dose prediction. Blood samples collected from mice in the efficacy study were analysed for the presence of parent as well as its active metabolite, compound **9** (*Pf* IC_50_ 14 nM). Compound **9** was evaluated in the *Pf*/SCID model to understand its PK-PD relationship (Supplementary Note 2, Supplementary Figs 4 and 5, and Supplementary Tables 4 and 5). Kill rate constant (*K*_kill_) and blood concentration required for half-maximal kill rate (EC_50_) were estimated by fitting a sigmoidal *E*_max_ model^13^,^14^,^15^,^16^. Efficacy data for **12** was modelled using the PK-PD model for parent plus metabolite assuming an additive effect^14^ (Supplementary Fig. 6 and Supplementary Table 6). The predicted and observed time course of parasitemia following treatment with **12** in the *Pf*/SCID model is shown in Fig. 2e. The blood levels of **9** as a metabolite did not contribute significantly to the observed efficacy of **12** (Supplementary Fig. 7). In addition, the formation of **9** in humans is predicted to be lower than in mice (Supplementary Note 3, Supplementary Figs 8 and 9, and Supplementary Table 7). Therefore, the *in vivo* activity of the metabolite was not considered for the human dose prediction. Compound **12** showed a good *in vitro–in vivo* correlation (IVIVC) between the intrinsic clearance (Cl_int_) in rat and dog hepatocytes and blood clearance (CL) observed *in vivo* in rat and dog, respectively (Supplementary Note 4). Compound **12** displayed a high volume of distribution (*V*_ss_) of 10–20 l kg^−1^, low CL (<35% liver blood flow) and long half-life (11–13 h in an intravenous (i.v.) PK and 14–16 h in an oral PK study) in rat and dog (Supplementary Figs 10 and 11 and Supplementary Tables 8 and 9). Human CL was predicted by *in vitro–in vivo* extrapolation from the *in vitro* Cl_int_ in human hepatocytes and by rat and dog allometry (Supplementary Note 5 and Supplementary Table 10). Human *V*_ss_ was predicted by rat and dog allometry (Supplementary Table 11). The predicted half-life of **12** in human is ~36 h. The predicted half-life of 36 h may not be long enough as compared with many known antimalarial drugs. However, it is sufficiently long for a fast-killing compound such as **12**, as compared with a fast-killing drug such as artemisinin with a half-life of 1 h in humans.

The predicted human exposure after a single dose of 260 mg is likely to sustain the maximum kill rate observed in the *Pf*/SCID model for 4–6 days to achieve >3-log reduction in parasitemia (Supplementary Note 6, Supplementary Figs 12 and 13, and Supplementary Table 12). A patient with clinical symptoms of uncomplicated malaria may have 1–2% parasitemia in blood, which is equivalent to 11–12 log parasites in total, assuming 13 log erythrocytes in human blood^17^. At a dose of 260 mg, compound **12** is predicted to maintain a constant kill rate over two life cycles (96 h) of the parasite to yield a 3 to 4-log reduction in the total parasite load. Therefore, a single-dose treatment with~500 mg might result in >8-log reduction in the parasite load. In a combination-based malaria therapy that is clinically practiced, an additive or synergistic effect of the partner drug with compound **12** is predicted to cure patients following a single-dose treatment.

### Genetic basis of resistance to TAPs

To gain insight into the molecular target, mutant selection was attempted with several TAPs against *Pf* Dd2 strain. The frequency of spontaneous resistance emergence under *in vitro* drug selection with TAPs was very low (<1 in 10^10^ asexual blood stage parasites). However, after ~200 days of increasing *in vitro* drug pressure, resistant mutants to **6** were obtained and cloned by limiting dilution. Five clones from two independent selections displayed a three- to six-fold increase in IC_50_ for **6** and **12** compared with the parental Dd2 line, while retaining susceptibility to other antimalarial agents (Supplementary Table 13). These findings provided compelling evidence that compound **6** and **12** act through the same genetic mechanism. Whole-genome sequencing revealed a novel G29V mutation in the vacuolar ATP synthase (V-type H^+^-ATPase) subunit D (PF13_0227) in all the clones, suggesting the involvement of this protein in conferring resistance to TAPs (Supplementary Table 14). T-coffee alignment of PF13_0227 with *Saccharomyces cerevisiae*, *Homo sapiens* and *Thermus thermophilus* shows the highest homology to subunit D (Supplementary Fig. 14). This gene is highly conserved in the natural parasite population; no non-synonymous mutations have been observed in 1,931 sequenced isolates from South/South East Asia (52%) and Africa (48%; available from the Pf3K Project, accessed as of 19 January 2015), suggesting that the gene is under mutational constraint, and that resistance to TAPs might not develop readily in the field.

This V-type H^+^-ATPase has been localized in the infected host and *Pf* plasma membrane and digestive vacuole, serving as a major route for ATP-dependent H^+^ efflux and pH regulation^18^,^19^,^20^,^21^. The V-type H^+^-ATPase is a large multisubunit complex composed of a proton-translocation domain (V_0_) and an ATP-hydrolytic domain (V_1_), where the subunit D is located^19^. Subunit D is part of the central stalk connecting the V_1_ and V_0_ domains, implicating a role in reversible dissociation of the V_1_V_0_ complex to conserve ATP or coupling of proton transport with ATP hydrolysis^19^. As demonstrated by specific inhibitors of V-type H^+^ ATPase, namely bafilomycin A_1_ (ref. 22) and concanamycin A^23^, inhibition of this pump is likely to result in parasite death due to physiological disturbances. However, specific inhibitors have so far failed to show potency *in vivo*^24^. TAPs being efficacious *in vivo* provide a novel therapeutic approach to treat malaria by targeting this essential proton pump. A very low rate of spontaneous resistance to TAPs coupled with the lack of point mutations in the V-type H^+^-ATPase gene from a diverse collection of global isolates confirm the lack of pre-existing mutations to this important gene in *Pf*.

To test whether the identified mutation in the V-type H^+^-ATPase subunit D protein affects the ionic homeostasis of the parasite’s vacuole, we determined the size of vacuoles relative to overall parasite size from microscopic Giemsa-stained images. Results showed vacuolar size ratios of the parent (Dd2-B2) and compound **6**-resistant mutant strains F2-D6 and F4-D9 cloned from two independent selections (Supplementary Fig. 15). Both mutants showed a relative increase of the digestive vacuole size of 23–28%, implying that the acquired mutation may impair vacuolar maintenance. Further investigations are necessary to characterize the physiological effects on the vacuolar pH, drug accumulation and ion homeostasis of drug-exposed *Pf* parasites.

### Safety studies of TAPs in rats and guinea pigs

**Figure 3:**
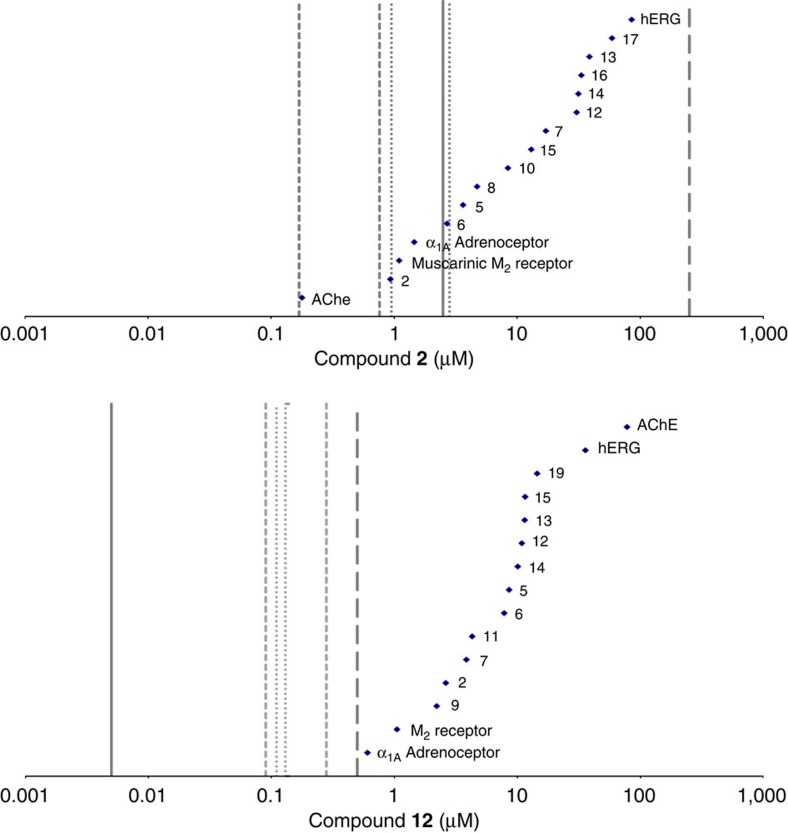
Improvement in safety margins results in the nomination of compound 12 as a clinical candidate. (**a**) Secondary pharmacology selectivity plot for compound **2**. Selectivity ratio for AChE, α_1A_ and M_2_ (IC_50_ against targets (♦)/blood *C*_min_ at ED_90_ in the *Pf*/SCID model (solid vertical line) was <1. Free plasma concentration range achieved in rat (dotted lines; *C*_min_ and *C*_max_) and guinea pig (dashed line; *C*_min_ and *C*_max_) *in vivo* illustrates targets covered during toxicity studies, and that selectivity against all targets was less than our target of >100-fold (long dash). (**b**) Secondary pharmacology selectivity plot for compound **12**. Blood *C*_min_ at ED_90_ in the *Pf*/SCID model (solid line) is significantly lower than that of compound **2**. Success in achieving desired *in vitro* selectivity illustrated as all off-target potencies (♦) sit to the right of our selectivity target: >100-fold (long dash). Free plasma concentration range achieved in rat (dotted lines; *C*_min_ and *C*_max_) and guinea pig (dashed line; *C*_min_ and *C*_max_) *in vivo* illustrates reduced pharmacological coverage during toxicity studies compared with compound **2**, which, in turn, translated into an improved safety margin for compound **12**.

To assess potential for *in vivo* toxicity mediated *via* secondary pharmacological effects, we profiled TAPs through a diverse panel of radioligand binding, enzyme activity and cellular functional assays covering 25 targets, plus hERG (Supplementary Table 15). The findings from the *in vivo* safety studies are described in Supplementary Note 7. The early lead, **2**, was active against several secondary targets (Fig. 3a). *In vivo* toxicity was observed for **2** in the rat and guinea pig models (Supplementary Table 16). However, only a handful of these targets were likely to be pharmacologically occupied at the systemic exposures achieved during those studies, leading to a hypothesis that the adverse events observed *in vivo* could be attributed to perturbation of AChE, α_1A_ adrenoceptor (antagonism) or muscarinic M_2_ receptor (antagonism). We established SAR during lead optimization, resulting in good *in vitro* selectivity against hERG and AChE, with moderate selectivity against α_1A_ adrenoceptor/M_2_ receptor (Fig. 2a). Despite the overall off-target profiles for **2** and **12** being similar (Fig. 3a,b), our success in reducing potency against our primary safety targets coupled with improvements in *Pf* potency (>60-fold) and reduction in free fraction (10-fold) translated into good safety margins for **12** *in vivo* in rat toxicity and guinea pig cardiovascular studies (Table 2 and Supplementary Fig. 16).

## Discussion

A phenotypic high-throughput screen of the AstraZeneca corporate library against the asexual blood stage of *Pf* resulted in the identification of TAPs as a novel class of antimalarial agents with multiple diversification points for lead optimization. TAPs display excellent *in vitro Pf* potency (*Pf* IC_50_ in the range of 5–30 nM) with good solubility and low *in vitro* CL_int_ during the lead identification phase. However, the series had secondary pharmacology-related liabilities, for example, AChE inhibition along with hERG inhibition. By using a diverse panel of radioligand binding enzyme and cellular functional assays covering a total of 85 targets, we established significant improvement in the *Pf* potency (IC_50_ <10 nM), with a concomitant improvement in off-target selectivity (*in vitro*) and good bioavailability (80%). An improved *in vivo* efficacy and selectivity profile of compound **12** translated to good safety margins in a 3-day rat toxicology study. No treatment-related effects in biochemical parameters, clinical pathology or histopathology were observed following dosing with **12** in the rats. These data support the progression of **12** into further preclinical toxicology studies, including second species cardiovascular telemetry. In conclusion, compound **12** with a novel mechanism of action, low frequency of resistance emergence, long half-life *in vivo* and ability to kill parasites rapidly is a potential clinical candidate for malaria.

## Methods

### Synthetic scheme for representative TAPs (compounds 7, 8, 9 and 12)

All reagents, starting materials and solvents described in the procedure were commercially available and used without further purification. Analytical thin-layer chromatography was performed on SiO_2_ plates on alumina and the purity of all final derivatives for biological testing was confirmed to be >95% using the following conditions: a Shimadzu HPLC (high-performance liquid chromatography) instrument with a Hamilton reverse-phase column (HxSil, C18, 3 μm, 2.1 mm × 50 mm (H2)). Eluent A: 5% CH_3_CN in H_2_O and eluent B: 90% CH_3_CN in H_2_O. A flow rate of 0.2 ml min^−1^ was used with ultraviolet detection at 254 and 214 nm. The structure of the intermediates and end products was confirmed by proton (^1^H), carbon (^13^C) nuclear magnetic resonance (NMR) and mass spectroscopy. Proton magnetic resonance spectra were determined in dimethylsulphoxide (DMSO) d_6_ unless otherwise stated, using Bruker DRX 300 or Bruker DRX-400 spectrometers, operating at 300 or 400 MHz, respectively. Splitting patterns are indicated as follows: s, singlet; d, doublet; t, triplet; m, multiplet; and br, broad peak. Liquid Chromatography-Mass Spectrometer (LC-MS) data was acquired using Agilent LCMS VL series. Source: Electron Spray Ionization (ESI), coupled with an Agilent 1100 series HPLC system and an Agilent 1100 series Photodiode Array (PDA) as the front end. high-resolution mass spectrometry (HRMS) data was acquired using an Agilent 6520, Quadrupole-Time of flight tandem mass spectrometry (MS/MS) coupled with an Agilent 1200 series HPLC system.

Generic synthetic route for the synthesis of key compounds (**7**, **8**, **9** and **12**) are illustrated in Fig. 1. The synthesis of compounds **1–6**, **10** and **11** are provided in Supplementary Note 1 using chemical scheme analogues to the one illustrated in Fig. 1 in the supplementary section.

A mixture of 5-bromouracil (90 g, 471 mmol, Aldrich) and tert-butyl (R)-2-methyl-4l2-piperazine-1-carboxylate (141 g, 706.8 mmol, Aldrich) were taken in pyridine (200 ml) to give a white suspension. The suspension was vortexed and subjected to microwave irradiation for 45 min at 150 °C to give a clear brown solution. Solvent was removed under vacuum and the residue was then triturated with ethyl acetate. The resultant suspension was filtered out and dried under vacuum to get (R)-tert-butyl-4-(2, 4-dioxo-1, 2, 3, 4-tetrahydropyrimidin-5-yl)-2-methylpiperazine-1-carboxylate (**I**, 39.6 g, 44%) as an off-white solid. Yield: 44%, purity: >95% by HPLC (ultraviolet at 220 and 254 nm). ^1^H NMR (300 MHz, DMSO-d_6_) *δ* 11.10 (s, 1H), 10.51 (s., 1H), 6.73 (d, *J*=4.7 Hz, 1H), 4.12 (s., H), 3.72 (d, *J*=13.2 Hz, 1H), 3.22–2.93 (m, 3H), 2.42 (dd, *J*=11.3, 3.6 Hz, 1H), 2.30 (d, *J*=2.8 Hz, 1H), 1.40 (s, 9H),1.19 (d, *J*=6.8 Hz, 3H). ^13^C-NMR (126 MHz, DMSO-d_6_) *δ* 161.20, 154.21, 150.73, 127.09, 126.62, 79.22, 54.63, 50.12, 46.88, 40.82. 39.15, 28.52 and 15.94, HRMS, electrospray ionization (HRMS (ESI)): *m/z* calculated for C_14_H_22_N_4_O_4_ +H [M+H]: 308.1532. Found: 308.3420.

Synthesis of (R)-rert-butyl 4-(2, 4-dichloropyrimidin-5-yl)-2-methylpiperazine-1-carboxylate (**II**), R)-tert-butyl 4-(2, 4-dioxo-1, 2, 3, 4-tetrahydropyrimidin-5-yl)-2-methylpiperazine-1-carboxylate (**II**, 22 g, 71.62 mmol) was taken in phosphorus oxychloride (750 ml), to give a brown suspension. The resulting reaction mixture was refluxed for 6 h and the excess phosphorus oxychloride was distilled out under reduced pressure. The remaining oil was diluted with THF (250 ml) under ice cold condition. Subsequently, the reaction mixture was basified to pH 8 with Na_2_CO_3_. To this di-tert-butyl dicarbonate (22.17 ml, 96.41 mmol, Aldrich) was added and stirred at room temperature for 16 h. The reaction mixture was diluted with methanol and filtered it off to remove excess salt. The solvent was removed under vacuum and residue was diluted with water and extracted with ethyl acetate. The combined organic layers were dried over sodium sulphate and then removed under reduced pressure. The crude residue was purified by flash chromatography on silica using ethyl acetate and hexane as eluents (1:4) to obtain pure solid of (R)-rert-butyl 4-(2, 4-dichloropyrimidin-5-yl)-2-methylpiperazine-1-carboxylate (**II**, 23.00 g, 93%). Yield: 93%, Purity: >95% by HPLC (ultraviolet at 220 and 254 nm). ^1^H NMR (300 MHz, CDCl_3_) δ 8.11 (s, 1H), 4.33 (s., 1H), 3.94 (d, *J*=13.9 Hz, 1 H), 3.29–3.15 (m, 3H), 2.93–2.70 (m, 2H) 1.47–1.37 (m, 9H), 1.31 (d, *J*=6.8 Hz, 3H). ^13^C-NMR (126 MHz, DMSO-d_6_) δ 155.65, 154.11, 152.94, 151.49, 143.31, 79.52, 54.82, 50.18, 28.50 and 15.54, HRMS (ESI): *m/z* calculated for C_14_H_20_Cl_2_N_4_O_2_ +H [M+H]: 347.1011. Found: 347.1140.

Synthesis of (R)-tert-butyl 4-(2-chloro-4-[(1, 5-dimethyl-1H-pyrazol-3-yl)amino)pyrimidin-5-yl]-2-methylpiperazine-1-carboxylate (**III**). To a mixture of (R)-tert-butyl 4-(2, 4-dichloropyrimidin-5-yl)-2-methylpiperazine-1-carboxylate (II, 6.92 g, 20 mmol) and 1,5-dimethyl-1H-pyrazol-3-amine (2.2 g, 20 mmol, Matrix scientific) in ethanol (100 ml) was added to DIPEA (5.30 ml, 30 mmol) and the reaction mixture was subjected to microwave irradiation at 110 °C for 1 h. The solvent was removed under vacuum and then ice cold water was added to the residue. The precipitated solid was filtered out and dried under vacuum. The crude product purified by flash chromatography on silica using dichloro methane and methanol as eluents (9:1) to obtain pure solid of (R)-tert-butyl 4-(2-chloro-4-((1,5-dimethyl-1H-pyrazol-3-yl)amino)pyrimidin-5-yl)-2-methylpiperazine-1-carboxylate (2.61 g). Yield: 31%, Purity: >95% by HPLC (ultraviolet at 220 and 254 nm). ^1^H NMR (300 MHz, MeOD) δ 7.99 (s, 1H), 6.61 (s., 1H), 4.39 (m, 1H), 3.99 (d, *J*=13.9 Hz, 1 H), 3.73( s, 3H), 3.4–3.50 (m, 1H), 3.0–3.15 (m, 2H) 2.89–2.92 (m, 2H), 2.70–2,77(m, 1H), 2.33(s, 3H), 1.37–1.48 (m, 12H). ^13^C-NMR (126 MHz, MeOD) δ 156.24, 155.17, 154.88, 146.93, 144.96, 140.28, 130.91, 97.11, 80.04, 55.56, 51.50, 34.43, 27.26, 14.26 and 9.82, HRMS (ESI)): *m/z* calculated for C_19_H_28_ClN_7_O_2_+H [M+H]: 422.1193. Found: 422.2010.

Synthesis of (R)-N4-(1, 5-dimethyl-1H-pyrazol-3-yl)-N2-(5-fluoro-6-methylpyridin-2-yl)-5-(3-methylpiperazin-1-yl)pyrimidine-2,4-diamine (7). To a solution of (R)-tert-butyl 4-(2-chloro-4-((1,5-dimethyl-1H-pyrazol-3-yl)amino)pyrimidin-5-yl)-2-methylpiperazine-1-carboxylate (**III**, 1.14 g, 2.71 mmol) dissolved in toluene (40 ml), were added 2-amino-5-fluoro-6-methylpyridine (0.513 g, 4.07 mmol, Aldrich), potassium tert-butoxide (0.46 g, 4.076 m mol) and degassed for 10 min. Then, BINAP (0.010 g, 0.16 mmol) and Pd_2_(dba)_3_(0.004 g, 0.00543 mol) were added and again degassed for 10 min. The reaction was then refluxed at 110 °C for 12 h. The reaction mixture was filtered through a celite bed and the organic layer was washed with brine, dried over anhydrous sodium sulphate and concentrated in vacuum. The crude residue was purified by flash chromatography on neutral alumina with gradient elution of 0.5–1.0% methanol in dichloromethane to get (R)-tert-butyl 4-(4-((1,5-dimethyl-1H-pyrazol-3-yl)amino)-2-((5-fluoro-6-methylpyridin-2-yl)amino)pyrimidin-5-yl)-2-methylpiperazine-1-carboxylate as an off-white solid. Then, the N-boc-protected precursor was subjected to boc group de-protection using 4 N HCl in dioxane (0.3 ml, Aldrich) at 10 °C with stirring for 15 min. The reaction mixture was concentrated under vacuum afforded the title compound **7** as light brown solid (450 mg). Yield: 31%, purity: >95% by HPLC (ultraviolet at 220 and 254 nm). ^1^H NMR (300 MHz, DMSO-d_6_) *δ* 11.51 (s, 1H), 10.25 (s, 1H) 9.52 (s, 1H) 9.10 (s, 1H) 8.11 (s, 1H) 7.83 (s, 1H) 7.28 (s, 1H) 6.68 (s, 1H ) 3.53–3.44 (m, 2 H) 3.38 (s, 3 H) 3.17–3.07 (m, 3H) 3.05–2.93 (m, 1H) 2.85–2.76 (m, 1H) 2.56 (d, *J*=2.8 Hz, 3H) 2.31 (s, 3H) 1.28 (d, *J*=5.6 Hz, 3H. ^13^C-NMR (126 MHz, DMSO-d_6_) *δ* 157.22, 149.36, 147.53, 143.39, 142.96, 139.26, 133.33, 126.63, 125.59, 112.59, 99.92, 47.79, 35.69, 17.08, 15.44 and 10.9, HRMS (ESI): *m/z* calculated for C_20_H_26_FN_9_+H [M+H]: 412.2295. Found: 412.2367.

The synthesis of 4-ethyl-5-fluoro-6-methylpyridin-2-amine (**8a**) and 4-cyclopropyl-5-fluoro-6-methylpyridin-2-amine (**9a**) was carried out as described earlier^10^.

4-Ethyl-5-fluoro-6-methylpyridin-2-amine (8a). Yield: 22%, purity: >95% by HPLC (ultraviolet at 220 and 254 nm). ^1^H NMR (300 MHz, DMSO-d_6_) *δ* 7.36 (s, 1 H), 2.63–2.81 (m, 2 H), 2.26 (s, 3 H) 1.18 (t, *J*=7.54 Hz, 3 H)). ^13^C-NMR (126 MHz, MeOD) *δ* 155.11, 151.24, 148.89, 140.41, 106.74, 21.60, 15.04 and 12.65, HRMS (ESI): *m/z* calculated for C_8_H_11_FN_2_+H [M+H]: 155.0906. Found: 155.0830.

4-Cyclopropyl-5-fluoro-6-methylpyridin-2-amine (9a), Yield: 22%, purity: >95% by HPLC (ultraviolet at 220 and 254 nm). ^1^H NMR (300 MHz, DMSO-d_6_) *δ* 14.19 (s., 1H), 7.49 (s., 2H), 6.33 (d, *J*=5.8 Hz, 1H) 2.37 (d, *J*=3.0 Hz, 3H) 2.18–1.97 (m, 1H) 1.27–1.14 (m, 2H) 0.92–0.77 (m, 2H). ^13^C-NMR (126 MHz, MeOD) *δ* 153.78, 152.05, 149.93, 147.61, 104.59, 11. 21, 10.58 and 9.16, HRMS (ESI): *m/z* calculated for C_9_H_11_FN_2_+H [M+H]: 167.0906. Found: 167. 0960.

Synthesis of (R)-N4-(1,5-dimethyl-1H-pyrazol-3-yl)-N2-(4-ethyl-5-fluoro-6-methylpyridin-2-yl)-5-(3-methylpiperazin-1-yl)pyrimidine-2,4-diamine dihydrochloride (8) is illustrated in Fig. 1 and synthesis procedure followed analogues to compound **7** using (R)-tert-butyl 4-(2-chloro-4-((1,5-dimethyl-1H-pyrazol-3-yl)amino)pyrimidin-5-yl)-2-methylpiperazine-1-carboxylate (III) and 4-ethyl-5-fluoro-6-methylpyridin-2-amine (8a). Yield: 22%, purity: >95% by HPLC (ultraviolet at 220 and 254 nm). ^1^H NMR (300 MHz, DMSO-d_6_) *δ* δ11.52 (s, 1H)10.28 (s, 1H) 9.82 (s, 1H) 9.40 (br. s., 1H) 8.13 (s, 1H) 7.18 (d, *J*=4.3 Hz, 1H) 6.74 (s, 1H) 3.76–3.60 (m, 4H) 3.54–3.24 (m, 4H) 3.22–2.96 (m, 3H) 2.85 (d, *J*=11.5 Hz, 1H) 2.67 (q, *J*=7.7 Hz, 2H) 2.54 (d, *J*=3.0 Hz, 3H) 2.31 (s, 3H) 1.29 (d, *J*=6.0 Hz, 3H) 1.24–1.15 (m, 3H); ^13^C-NMR (126 MHz, MeOD) *δ* 156.24, 155.17, 154.88, 146.93, 144.96, 140.28, 130.91, 97.11, 80.04, 55.56, 51.10, 34.43, 27.65, 14.26 and 9.82, HRMS (ESI): *m/z* calculated for C_22_H_30_FN_9_+H [M+H]: 440.2608. Found: 440.2500.

Synthesis of N2-(4-cyclopropyl-5-fluoro-6-methylpyridin-2-yl)-N4-(1, 5-dimethyl-1H-pyrazol-3-yl)-5-(3-methylpiperazin-1-yl)pyrimidine-2,4-diamine dihydrochloride (9) is illustrated in Fig. 1 and the synthesis procedure followed analogues to compound **7** using (R)-tert-butyl4-(2-chloro-4-((1,5-dimethyl-1H-pyrazol-3-yl)amino)pyrimidin-5-yl)-2-methylpiperazine-1-carboxylate (III) and 4-cyclopropyl-5-fluoro-6-methylpyridin-2-amine (9a). Yield: 25%, purity: >95% by HPLC (ultraviolet at 220 and 254 nm). ^1^H NMR (300 MHz, DMSO-d_6_) *δ* 13.93 (s., 1H)11.39 (s, 1H) 10.29 (s, 1H) 9.81 (s., 1H) 9.38 (s., 1H) 8.12 (s, 1H) 6.89–6.64 (m, 2H) 3.42 (s., 1H) 3.80–3.56 (m, 4H) 3.38–3.25 (m, 1H) 3.18–2.95 (m, 3H) 2.89–2.76 (m, 1H) 2.54 (d, *J*=3.2 Hz, 3H) 2.31 (s, 3H) 2.13 (t, *J*=4.7 Hz, 1H) 1.29 (d, *J*=6.2 Hz, 3H) 1.22–1.15 (m, 2H) 0.82–0.73 (m, 2H). ^13^C-NMR (126 MHz, DMO-d_6_) *δ* 154.49, 154.03, 152.15, 150.22, 148.03, 146.16. 144.60, 141.40, 139.51, 138.12, 124.82, 105.50, 96.42, 58.92, 51.62, 34.58, 18.69. 16.55, 10.30 and 8.05, HRMS (ESI): *m/z* calculated for C_23_H_30_FN_9_ +H [M+H]: 452.2608. Found: 452.2683.

Synthesis of (R)-N2-(4-cyclopropyl-5-fluoro-6-methylpyridin-2-yl)-N4-(1, 5-dimethyl-1H-pyrazol-3-yl)-5-(3, 4-dimethylpiperazin-1-yl)pyrimidine-2,4-diamine (**12**). (R)-N2-(4-cyclopropyl-5-fluoro-6-methylpyridin-2-yl)-N4-(1,5-dimethyl-1H-pyrazol-3-yl)-5-(3-methylpiperazin-1-yl)pyrimidine-2,4-diamine hydrochloride (compound **9**, 190 mg, 0.42 mmol) was taken in dichloromethane (2 ml) to give a yellow suspension. To this Hunig's Base (0.184 ml, 1.05 mmol) was added and the suspension turned clear. After 10 min of stirring, reaction mixture turned into a white suspension and then it was concentrated to dryness. Resultant residue was dissolved in ethanol (absolute, 99.5%) (3 ml), and formaldehyde (0.042 ml, 0.63 mmol) was added and stirred for 10 min. To this clear solution, sodium cyanoborohydride (26.4 mg, 0.42 mmol) was added in one portion to get a white suspension. The reaction mixture was concentrated and the crude product was purified through reverse-phase chromatography to get the pure off-white solid of (R)-N2-(4-cyclopropyl-5-fluoro-6-methylpyridin-2-yl)-N4-(1, 5-dimethyl-1H-pyrazol-3-yl)-5-(3,4-dimethylpiperazin-1-yl)pyrimidine-2,4-diamine (80 mg, 40.8%). Yield: 40.8%, purity: >95% by HPLC (ultraviolet at 220 and 254 nm). ^1^H NMR (300 MHz, DMSO-d_6_) *δ* 9.26 (s,1H), 8.03 (s, 1H) 8.00 (s, 1H) 7.67 (d, *J*=5.1 Hz, 1H) 6.83 (s, 1H) 3.33 (s, 3H) 2.96–2.73 (m, 4H) 2.75–2.50 (m, 1H) 2.38–2.30 (m, 4H) 2.23 (s, 7H) 2.10–1.96 (m, 1H),1.08–1.02 (m, 2H) 1.00 (d, *J*=6.2 Hz, 3H) 0.78–0.67 (m, 2H). ^13^C-NMR (126 MHz, DMO-d_6_) *δ* 155.30, 154.67, 152.10, 150.93, 148.98, 146.81. 145.29, 141.95, 140.31, 138.81, 124.91, 106.20, 97.07, 58.78, 51.87, 42.16, 35.28, 17.23. 10.99 and 8.77, HRMS (ESI): *m/z* calculated for C_24_H_32_FN_9_+H [M+H]: 466.2765. Found: 466. 2838. Traces of LC-MS, HRMS, ^1^H NMR and ^13^C-NMR of compound **12** are shown in Supplementary Figs 1–3.

### Assessment of cross-resistance to antimalarial agents

To eliminate overlapping mode of action with known targets, cross-resistance testing of representative TAPs against drug-resistant lines with well-characterized mutations in *Pf* dihydroorotate dehydrogenase^25^, *Pf* heat-shock protein 90, *Pf* chloroquine resistance transporter^25^ or *Pf* cytochrome *b* reduction site^26^ was performed. Mutations in these genes contribute to resistance to antimalarial agents under development, namely Genz-669178, geldanamycin, IDI-3783 or IDI-5918, respectively. To assess cross-resistance, the IC_50_ or *Pf* growth inhibition was measured using the SYBR green growth assay—the standard non-radioactive, high-throughput 72-h assay that uses a nucleic acid-binding fluorescent dye^27^,^28^. Assays were carried out in 384-well format with three technical replicates within an assay day and biological replicates carried out on three different assay days to assign a final EC_50_.

### Determination of efficacy in the *Pf*/SCID mouse model

All animal studies were ethically reviewed and carried out in accordance with European Directive 86/609/EEC and the GSK Policy on the Care, Welfare and Treatment of Animals. During this experiment, female mice were used from the batch NSGJ 01/1 (The Jackson Laboratory, USA). The therapeutic efficacy of compound **12** against *Pf*3D7^0087/N9^ was studied using a ‘4-day test’ reported earlier^12^. Briefly, NODscidIL2Rγ^null^ mice engrafted with human erythrocytes were infected with 20 × 10^6^ *Pf*-infected erythrocytes. Infections were performed by i.v. inoculation. All mice were randomly assigned to their corresponding treatment. The treatment started at day 3 and finished at day 6 after infection. The compound was administered orally as a suspension in 0.5% w/v hydroxypropyl methylcellulose (HPMC) with 0.1% v/v Tween 80, using an oral gavage at a maximum dose volume of 20 ml kg^−1^. Vehicle alone was administered in untreated mice. In all cases, parasitemia was assessed in samples from peripheral blood obtained at days 3–7 after infection. A qualitative analysis of the effect of treatment on *Pf*3D7^0087/N9^ was assessed by microscopy and flow cytometry. Fresh samples of peripheral blood from *Pf*-infected mice were stained with TER-119-Phycoerythrine (marker of murine erythrocytes) and SYTO-16 (nucleic acid dye) and then analysed by flow cytometry (FACS Calibur, BD). Microscopy analysis was performed with Giemsa-stained blood smears from samples taken at days 5 and 7 (48 and 96 h after starting treatment, respectively).

### PKs in the blood of infected *Pf*/SCID mice

Peripheral blood samples (25 μl) were taken at different times (0.25, 0.5, 1, 2, 4, 6, 8 and 23 h), mixed with 25 μl of H_2_O Mili-Q and immediately frozen on dry ice. The frozen samples were stored at −80 °C until analysis. Vehicle-treated mice experienced the same blood-sampling regimen. Blood samples were processed by liquid–liquid extraction by mixing 10 μl diluted blood with 180 μl AcN:MeOH (80:20; v-v) mixture. Quantitative analysis by Liquid chromatography-tandem mass spectrometry (LC-MS/MS) was performed using UPLC (Waters) and Sciex API4000. The lower limit of quantification in this assay was 0.005 μg ml^−1^.

### *In vitro* generation of drug-resistant *Pf* lines

In order to identify the mode of action of TAPs, a chemogenomic approach was used whereby resistant parasites are generated by *in vitro* selection under drug pressure and whole-genome sequencing is used to identify the genetic basis of resistance^29^. Generation of *in vitro* resistance to compound **6** was performed as described^26^ with the following modifications. Four independent selections (F1-4) each with Dd2 parasites at an initial population of 10^8^–10^9^ were subjected to drug pressure at 10 × IC_50_ between 2 and 8 days or until all parasites were dead. The drug was then washed off and the parasites were allowed to recover in normal growth media. Four rounds of intermittent drug pressure at 10 × IC_50_ were performed. On the fifth round, the drug pressure was increased to 20 × IC_50_. Drug resistance was confirmed by measuring IC_50_s with the SYBR green growth assay as described above. After ~200 days of intermittent drug pressure, drug-resistant lines were cloned from F2 and F4 by limiting dilution for single-cell isolation^30^, genomic DNA extracted using the QIAmp DNA Blood Mini Kit (Qiagen, USA) and whole-genome sequenced as described below.

### Whole-genome sequencing and analyses

Raw reads were analysed with a customized pipeline based on the GATK toolkit version 2.8.1. In summary, the raw reads where aligned with the BWA-aligner^31^, PCR duplicates were removed, Indel regions were realigned and the resulting assemblies were base-recalibrated using the known single-nucleotide polymorphism (SNP) database for Dd2 parasites (PlasmoDB 11.0). Next, a hard filter was applied in order to mark positions of low-quality scores according to the GATK best practices recommendations for haploid genomes^32^. These regions were not excluded from the final analysis. Only SNPs within genes^33^ were considered and *Pf* variant genes and surface antigens were excluded from the analysis. The results of all strains were combined to evaluate whether the identified SNPs are conserved within different mutants. High-quality SNPs were called if the SNP was identified by at least 87.5% of all reads from the corresponding position, otherwise the SNPs were considered polymorphic.

### Data retrieval

 Next-Generation Sequencing (NGS) assembly files of all *Pf* strains used in this study to identify the resistance determinants can be accessed from the Sequence Read Archive at the NCBI (http://www.ncbi.nlm.nih.gov/sra/?term=SRP052918). Please consult the Sequence Read Archive toolkit documentation (http://www.ncbi.nlm.nih.gov/sites/books/NBK158900/) on how to extract the corresponding read data. The reference used for assembly was taken from PlasmoDB.org, version 11.0 (http://plasmodb.org/common/downloads/release-11.0/Pfalciparum3D7/fasta/) and reordered as indicated in the assembly’s file header. The MD5 checksum of the reference file is included in the @CO header line.

### hERG profiling and assessment of cytotoxicity of TAPs

hERG IC_50_ was determined in an electrophysiology-based hERG assay using IonWorks HT (CHO cells) as described earlier^34^.

The THP-1 cytotoxicity screen was based upon determination of fluorescent signal generated by the reduction of non-fluorescent resazurin (7-hydroxy-3H-phenoxazin-3-one 10-oxide) to the fluorescent resorufin (Alamar blue assay). Cellular reduction of resazurin is dependent on a pool of reductase or diaphorase enzymes derived from the mitochondria and cytosol. Therefore, Alamar blue can be used as an oxidation–reduction indicator in cell viability assays for mammalian cells^35^,^36^.

### *In vitro* safety pharmacology profiling of TAPs

An *in vitro* pharmacological profiling panel was specifically designed for the detection of potential high- risk clinical adverse drug reactions. The panel comprised of 26 diverse targets (6 enzymes, 2 transporters, 9 G-protein-coupled receptors (GPCRs), 5 ion channels along with hERG, M2, alpha1A and AchE) and was conduced externally by CEREP, France and was conducted externally by CEREP, France. For assessment of activity against these targets, compounds were tested in an 8-point concentration–response covering half-log units up to a top test concentration of 100 μM, and an IC_50_ or *K*_i_ was determined, using either radioligand binding assay or an enzyme activity assay (HTRF, LANCE). Detailed methodology for *in vitro* pharmacological profiling assays provided by CEREP can be found at http://www.cerep.fr, with specific reference to the AChE (cat. no. 0363), Muscarinic M_1_ receptor (cat. no. 0091) and Adrenergic α_1A_ receptor (cat. no. 2338) assays. For enzyme and kinase assays, the results were expressed as a percent inhibition of control-specific activity obtained in the presence of the test compound. The IC_50_ values (concentration causing a half-maximal inhibition of control-specific activity) and Hill coefficients (*n*_H_) were determined by nonlinear regression analysis of the inhibition/concentration–response curves using Hill equation curve fitting as described earlier^37^.

For radioligand binding assays, the results are expressed as % inhibition of control-specific binding obtained in the presence of the test compounds. The IC_50_ values (concentration causing a half-maximal inhibition of control-specific binding) and Hill coefficients (*n*_H_) were determined by nonlinear regression analysis of the competition curves generated with mean replicate values using Hill equation curve fitting. The inhibition constants (*K*_i_) were subsequently calculated by applying the Cheng Prusoff equation^38^.

All secondary pharmacology data for both compounds **2** and **12** from the panel were specifically tailored to this compound series.

### Methodology for 3-day rat toxicity study

The study was conducted at AstraZeneca R&D, Alderley Park, Cheshire, UK. All animal studies were undertaken in accordance with the AstraZeneca Bioethics Policy, which requires that work involving animals is carefully considered and justified to ensure that the study is scientifically necessary, and that the principles of the 3Rs (replacement, reduction and refinement) are applied. Animal studies were conducted in compliance with all relevant local and national laws and regulations, and with the principles of the ‘Guide for the Care and Use of Laboratory Animals’ 8th edition (Institute for Laboratory Animal Research).

Two groups of male rats (*n*=3, Han Wistar) were obtained from Charles River Laboratories, UK. Animals were group housed for ~1 week before the initiation of dosing. On the first day of dosing, rats were ~11 weeks of age and weighed 250–300 g and received compound **12** via oral gavage at dose levels of 75 or 150 mg kg^−1^ for three consecutive days and necropsied 24 h after the final dose. Vehicle: 1% pluronic F127; dose volume: 10 ml kg^−1^. In addition to regular monitoring of the animals' general condition, toxicokinetic profiles were obtained on day 1 and clinical chemistry, haematology and histopathology were assessed from all animals on day 4. Female animals were not included in the study as sex differences in either exposure or tolerability were not expected and control animals were excluded from this preliminary study design to reduce animal usage. Body weights were collected during acclimation and before each dose. Food and water consumption were available *ad libitum* but not recorded.

Toxicokinetic profiles were obtained on day 1 from all animals (time points: 0.5, 1, 2, 4, 6 and 24 h post dose) via microsample tail prick (32 μl blood) and were analysed using a qualified analytical procedure. Clinical pathology blood samples were taken at necropsy (24 h after third and final dose) from all animals, and analysed following a routine toxicological parameter list. Urinalysis was not performed. A limited list of key organs were taken at necropsy, processed and examined microscopically to determine whether treatment resulted in any clear pathological effects (heart, kidneys, liver, lungs and gastrointestinal tract).

### Results of 3-day rat toxicity study

No abnormalities were observed in the behaviour or general condition of the animals during treatment. Analysis of the clinical pathology and histopathology revealed no findings considered related to treatment with compound **12**. As no findings of significance were observed, these data are not presented here.

### Design of anaesthetized guinea pig cardiovascular study

The study was conducted in accordance with the European Union (EU) animal welfare regulation for animal use in experimentation (European Directive 2010/63/EEC) and approved by Biotrial Ethical Committee ‘Comité de reflexion Ethique en Expérimentation Animale (CR2EA)’.

Male Dunkin Hartley guinea pigs (*n*=6 per group; 342–401 g; 4–5 weeks old; Charles River Laboratories, L’Arbresle, Cedex) were anaesthetized with sodium pentobarbitone (60 mg kg^−1^ intraperitoneally followed by 6 mg kg^−1^ h^−1^ i.v. (jugular) for maintenance) and mechanically ventilated. Animals were surgically prepared for the measurement of haemodynamic (blood pressure, heart rate and contractility (including left ventricular pressure)) and electrocardiographic (ECG)-derived parameters (QTcB, PR interval and QRS duration). Briefly, catheters were introduced into a carotid artery and a jugular vein for the measurement of arterial blood pressure and administration of the test item, respectively. Needle electrodes were placed subcutaneously in a lead II position for ECG recording. A fluid-filled catheter was introduced into the left ventricle via the opposite carotid artery for left ventricular pressure measurement.

Animals received two consecutive i.v. infusions of either vehicle (group 1; 40% v/v dimethylacetamide/20% v/v polyethylene glycol 400/17% w/v hydroxyl propyl beta cyclodextrin (HPβCD/kleptose) in water for injection) or compound **12** at 10 or 30 mg kg^−1^ (group 2). Each infusion lasted for 15 min at a dose volume of 3 ml kg^−1^ (rate of 0.2 ml kg^−1^ min^−1^). Animals were monitored for a further 30-min washout period. Blood samples were taken at 5, 10 and 15 min after the start of each infusion and at 5, 10, 20 and 30 min during the washout phase.

Following a stabilization period, haemodynamic and ECG parameters were measured (using HEM software, version 4.3, Notocord Systems) at 10 and 5 min before administration (mean of measurements obtained at these time points=baseline; T0), and 2.5, 5, 10 and 15 min after the start of each i.v. infusion of compound **12** or vehicle and 5, 10, 20 and 30 min after the end of the second infusion. A detailed description of the model, its background and validation has been described earlier^39^,^40^.

All parameters were analysed statistically using SAS software. Baseline values were defined as mean of measurments taken 10 or 5 min before dosing. For each parameter, homogeneity of baseline values (T0) between the two groups were tested using a two-tailed Student’s *t*-test. Changes from baseline were assessed using a two-way analysis of variance (group and time) with repeated measurements over the time and group × time interaction.

## Author contributions

S.H.P., S.S., S.K. and V.K.S. designed and directed the study and wrote the manuscript with contributions from all co-authors; S.H.P., V.P., K.M., J.P., E.B., G.S., S.L., K.K., A.R., N.R.C., S.M., S.R., B.B., P.W. and P.S.I., were responsible for medicinal chemistry design, organic synthesis of compounds and data analyses; S.S., S.B., A.S., V.P., R.S., J.R., K.R.P., A.D. and V.H. were responsible for the pharmacokinetics and pharmacodynamics study design and data analysis; P.V., D.A., S.M., N.R., A.A., R.N., M.C., K.M., V.B., S.N. and V.K.S. were responsible for the biological profiling of compounds in various biological assays and data analysis; L.R-R., K.H. and S.K. were responsible for designing the *in vitro* safety pharmacology and *in vivo* safety studies and for data analysis; M.B.J.-D., M.S.M. and L.M.S., were responsible for determining the *in vitro* parasite reduction ratio and evaluating the efficacy parameters in the *Pf*/SCID model; P.A.M., A.K.L. and D.F.W. were responsible for generating cross-resistance and mutant selection data. O.C-F. and D.A.F. were responsible for generating cross-resistance data and mutant selection data; P.P.H. was responsible for measuring the parasite vacuolar size and whole-genome sequence data analysis; D.W. contributed to chemistry design; R.E.M was responsible for generating the whole-genome sequence data of resistant mutants.

## Additional information

**Accession codes:** NGS assembly files of all *Pf* strains used in this study to identify the resistance determinants have been deposited in the NCBI Sequence Read Archive (SRA) with accession code SRP052918.

**How to cite this article:** Hameed P. S. *et al.* Triaminopyrimidine is a fast-killing and long-acting antimalarial clinical candidate. *Nat. Commun.* 6:6715 doi: 10.1038/ncomms7715 (2015).

## Supplementary Material

Supplementary Information Supplementary Figures 1-16, Supplementary Tables 1-16, Supplementary Notes 1-7 and Supplementary References

## Figures and Tables

**Table 1:** Structure activity relationship and cytotoxicity selectivity index for compounds **1**–**12**.

**Table 2:** Summary of the findings from the preclinical *in vivo* toxicology studies.

**Study**	**Dose (mg kg**^−1^ **per day)**	**A—plasma** ***C***_**max**_**(μM) free**	**Summary of clinical signs and effects**	**B—predicted mean human plasma** ***C***_**max**_ **(μM) free**	**Safety margin (A**/**B)-free** ***C***_**max**_
**3-Day rat tolerability**	75 (Oral)	0.11	NOEL	0.0087	12.6
	150 (Oral)	0.13	NOEL	0.0087	15
**Acute anaesthetized guinea pig CVS** **model**	10 (i.v.)	0.09	NOEL	0.0087	10.3
	30 (i.v.)	0.28	Contractility and QTcB effects	0.0087	32.2

i.v., intravenous; NOEL, no observed effect level; *C*_max_, maximum concentration reached in plasma; cardiovascular (CVS); QTcB, heart rate-corrected QT interval.

Safety margins are based upon the predicted human plasma *C*_max_ at 260 mg dose.
