# Supplementary material for: Triaminopyrimidine is a fast-killing and long-acting antimalarial clinical candidate
Source: Nat Commun. 2015 Mar 31;6:6715. doi: 10.1038/ncomms7715 (PMC4389225; doi:10.1038/ncomms7715)
Supplement: Supplementary Information — Supplementary Figures 1-16, Supplementary Tables 1-16, Supplementary Notes 1-7 and Supplementary References [file ncomms7715-s1.pdf]

Supplementary Fig.1: LC-MS chromatogram of compound 12.

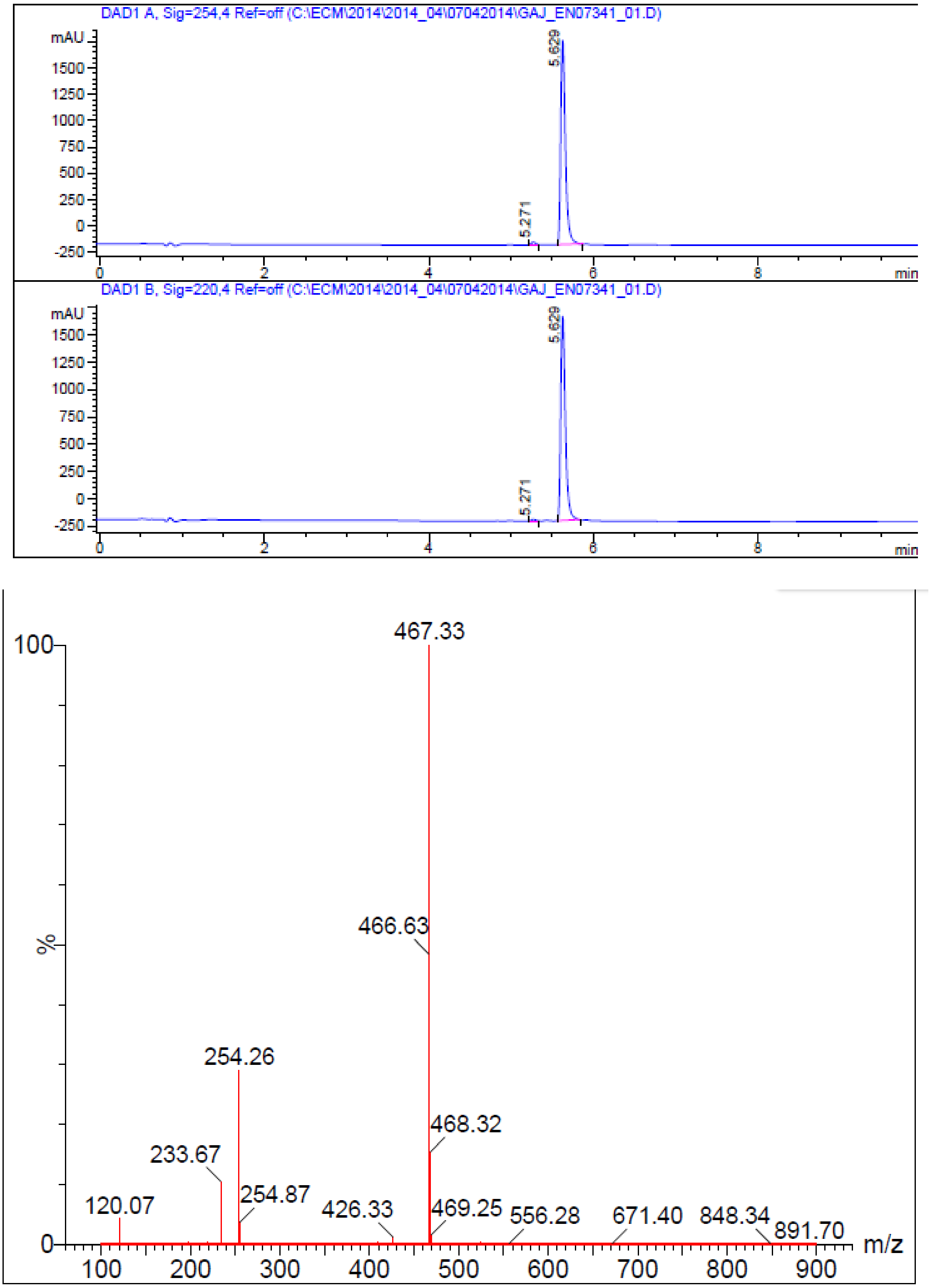

Supplementary Fig. 2: HRMS profile of compound 12.

[illegible]

Fragmentor Voltage 150 Collision Energy 0 Ionization Mode ESI

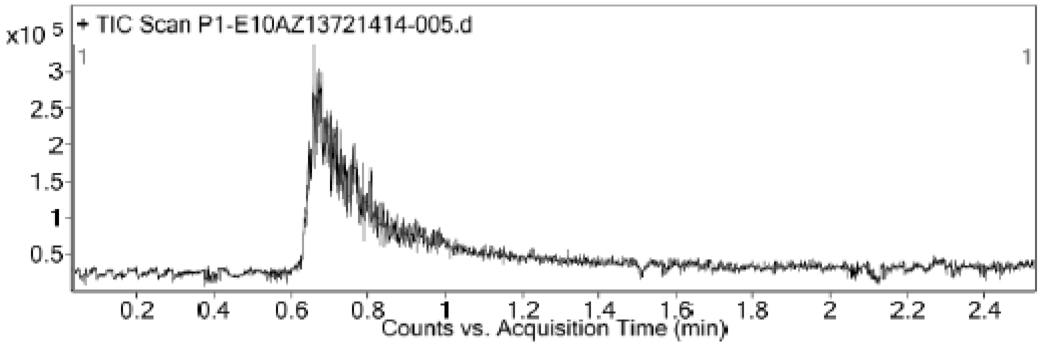

User Spectra

Fragmentor Voltage	Collision Energy	Ionization Mode
150	0	ESI

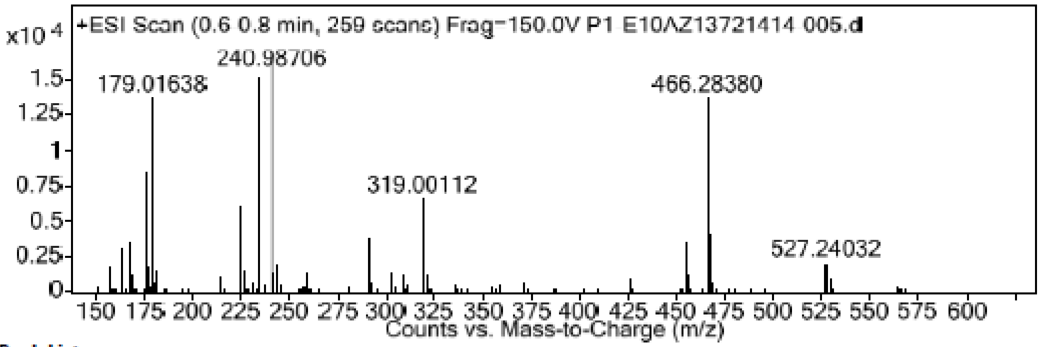

Peak List

m/z	z	Abund
176.00805	2	8399
179.01638		13670
225.00937	1	5983
233.64508	2	15070
234.14636	2	4695
240.98706	1	16517
291.14521		3802
319.00112		6512
466.2838	1	13673
467.28625	1	4054

Supplementary Fig. 3: ^{1}H NMR (top) and ^{13}C NMR (bottom) of compound 12.

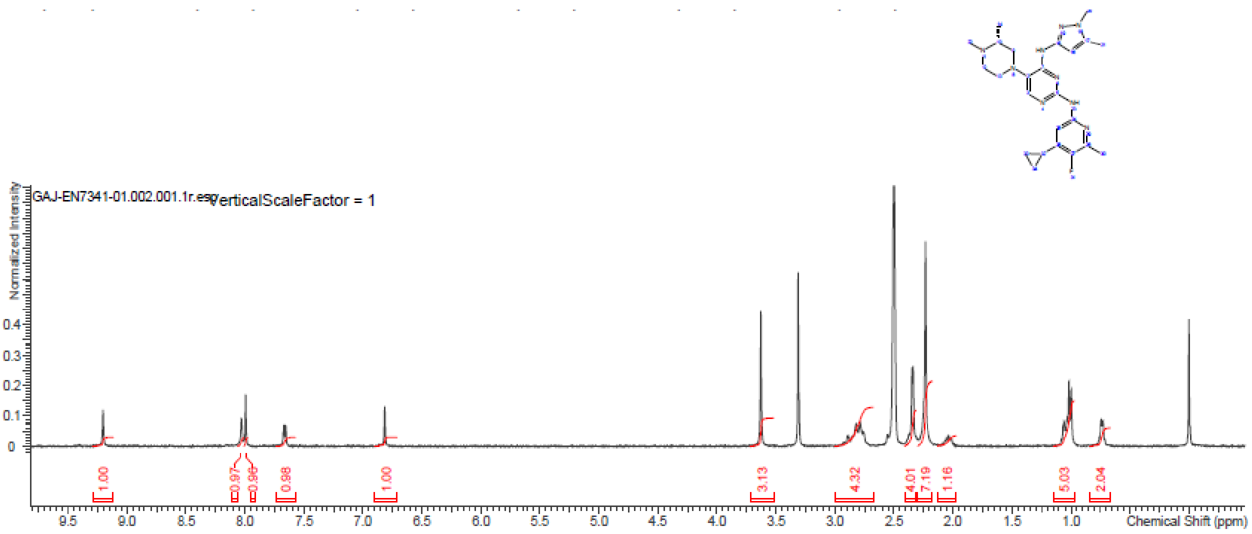

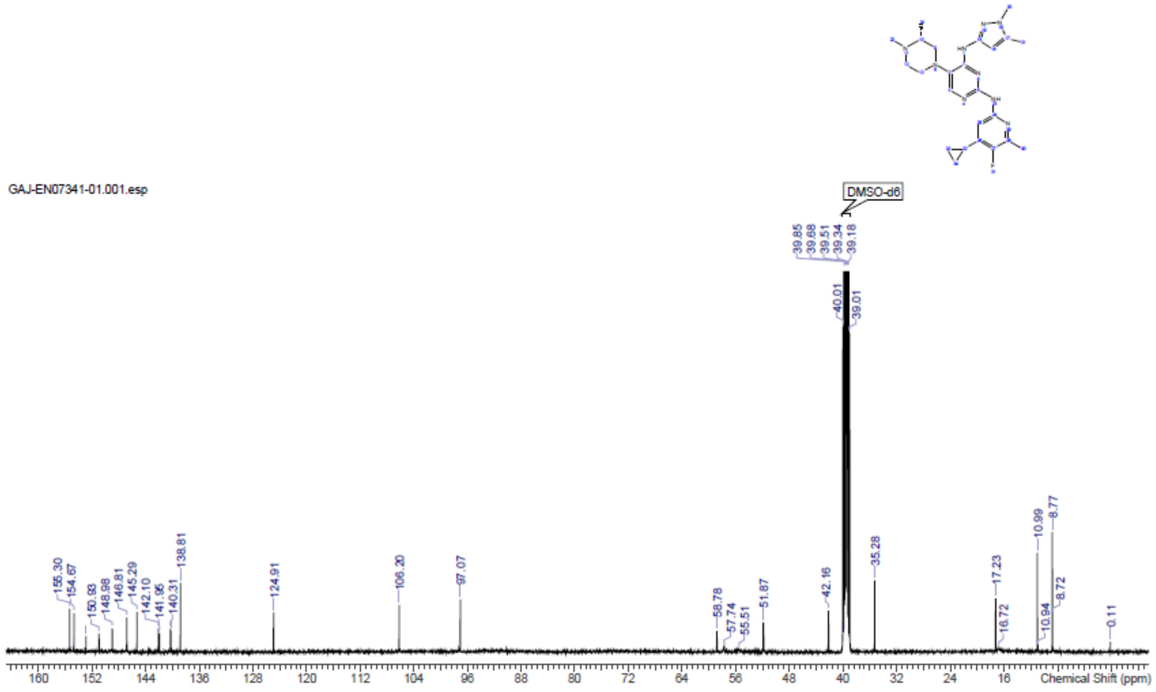

Supplementary Fig. 4: The observed (symbols) and predicted (lines) blood PK profiles of compound 9 in mouse.

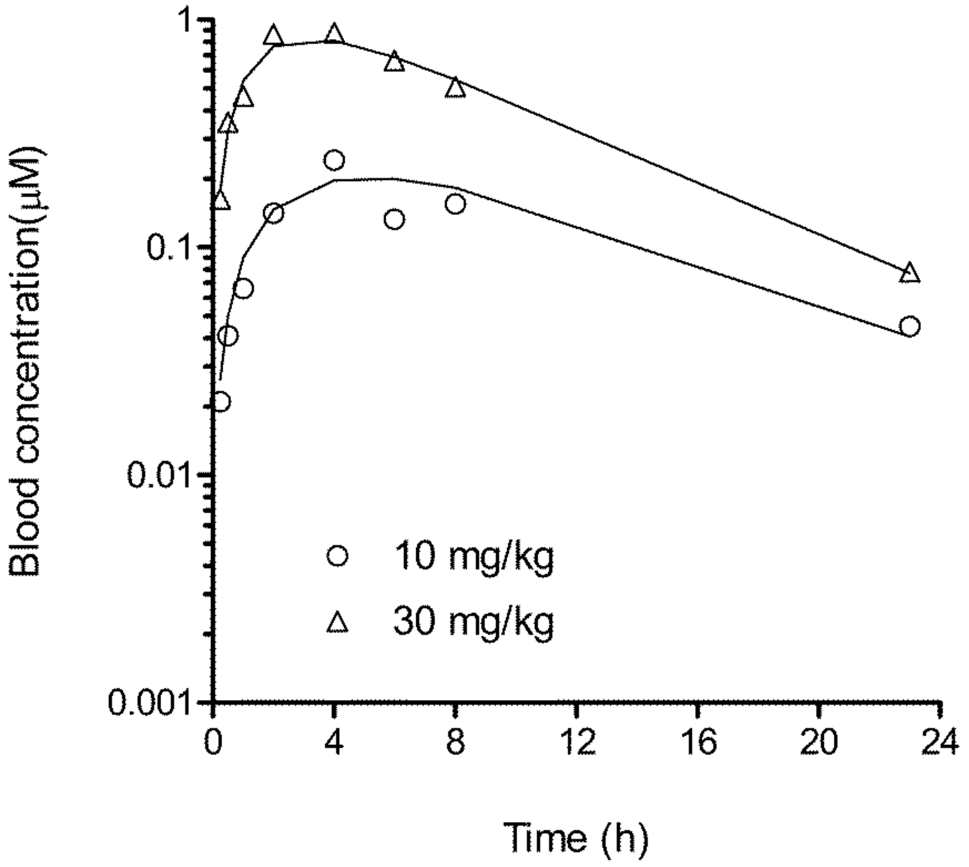

Supplementary Fig. 5: **The observed and predicted change in total parasites in the mouse after treatment with compound 9 dosed at 10 or 30 mg/kg or with vehicle (untreated).**

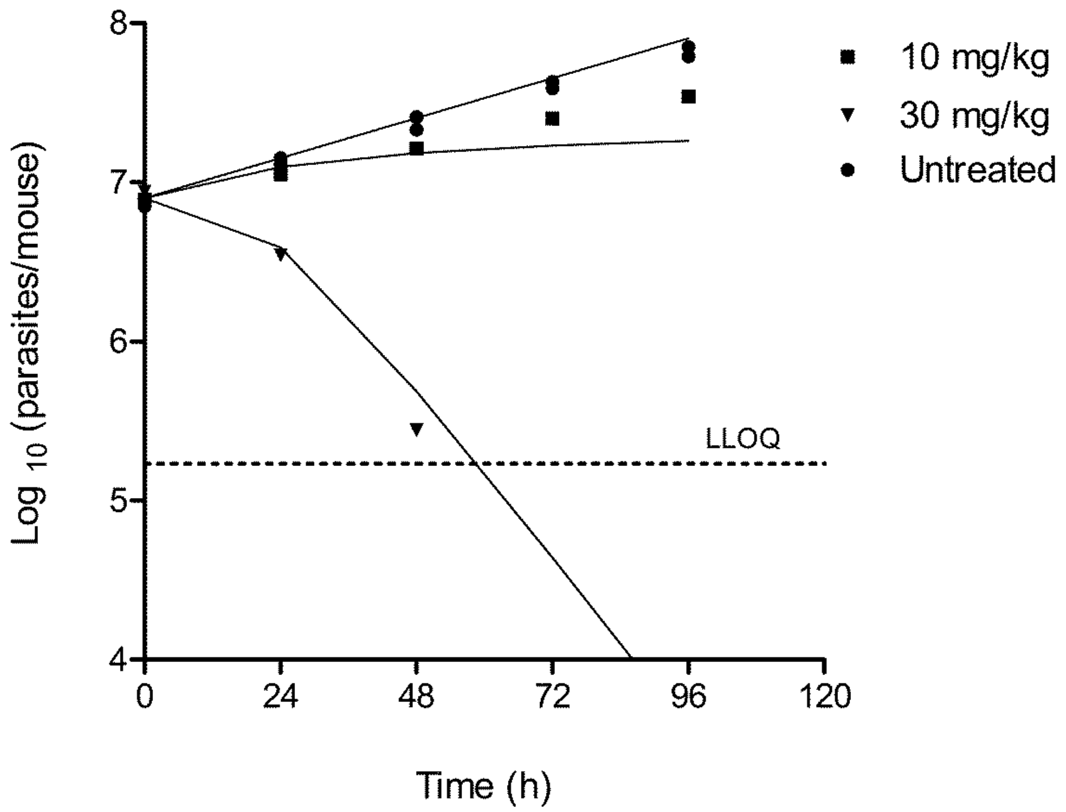

Supplementary Figure 6: PK-PD model for the parent (compound 12) plus metabolite (compound 9).

The active metabolite was generated *in vivo* assuming conversion of the parent to this metabolite was the only clearance mechanism for the parent. The predicted (lines) and observed (symbols) blood PK profiles of the parent and the metabolite at 10, 20, 40 and 80 mg/kg dose of **12** are shown below.

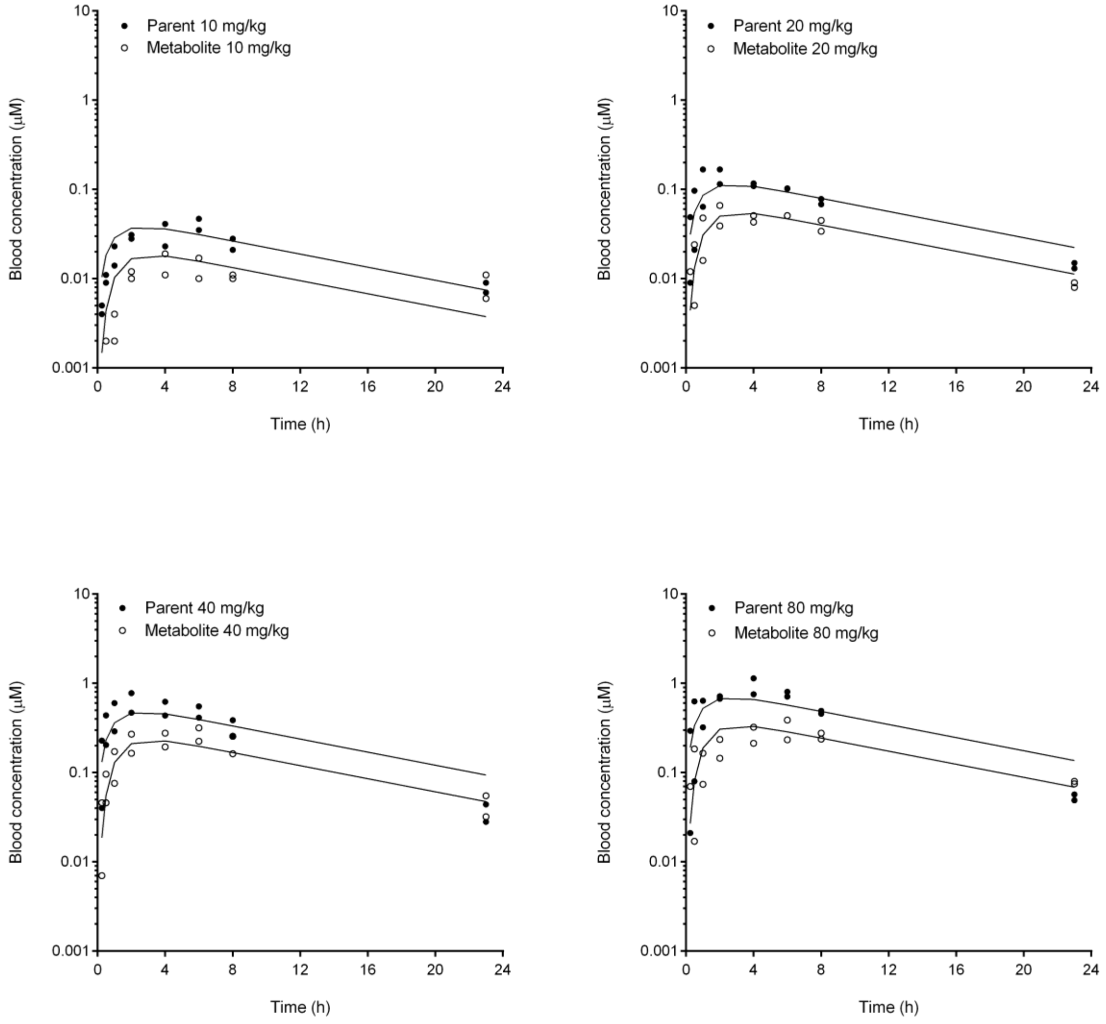

Supplementary Figure 7: Relative contribution of parent (compound 12) and metabolite (compound 9) towards efficacy in the *Pf*/SCID model.

The contribution of an active metabolite, compound **9**, generated in the *Pf*/SCID mouse model to the efficacy observed for the parent, compound **12**, was assessed by simulating efficacy with metabolite (K_{kill} for the metabolite = 0.2.h^{-1}) and without metabolite (K_{kill} for the metabolite = 0.02.h^{-1}). Difference in the predicted efficacy with and without metabolite was insignificant.

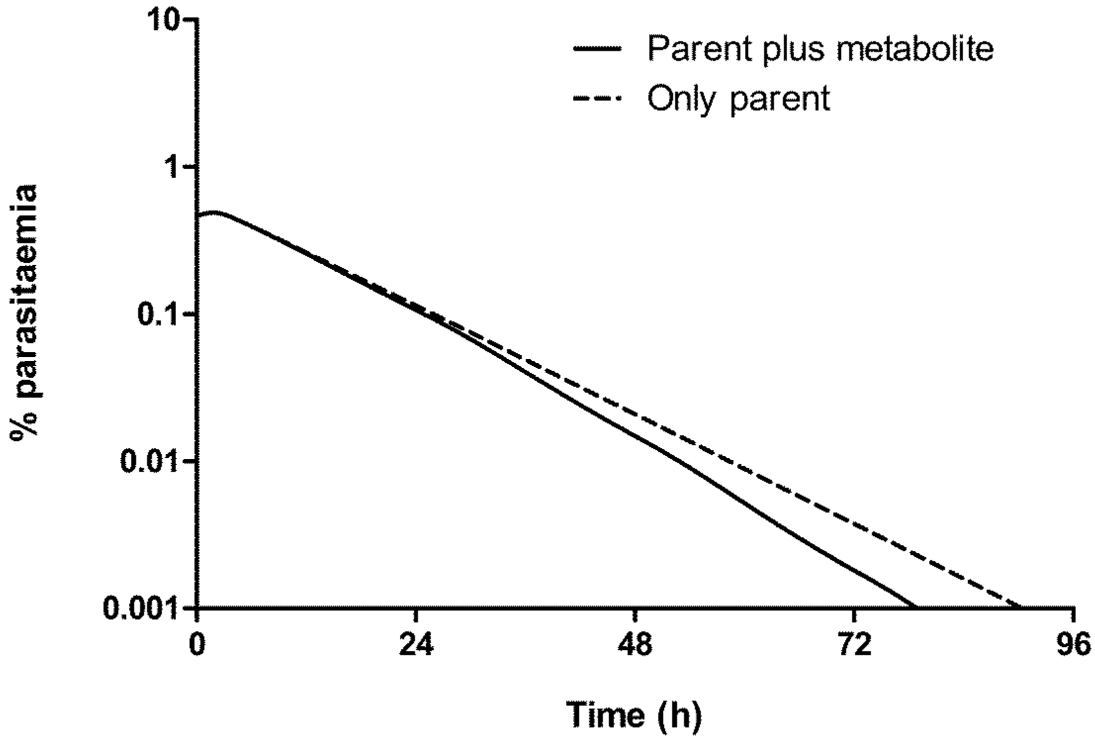

Supplementary Figure 8: **Michaelis-Menten plot for the formation of compound 9.**

The observed (symbols) and predicted (lines) data is shown below.

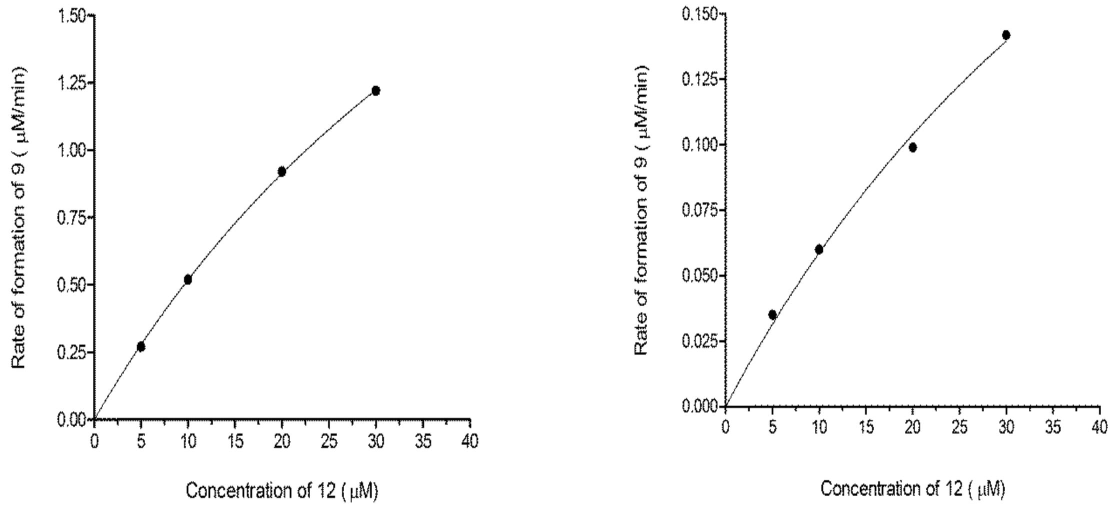

Formation of metabolite *in vitro* was modelled using a Michaelis-Menten equation:

$$V = V_{max} * S/(K_m + S)$$

Supplementary Figure 9: **Time course of *in vitro* metabolism.**

This model was simultaneously fit to the time course of the parent depletion and the metabolite formation as shown below:

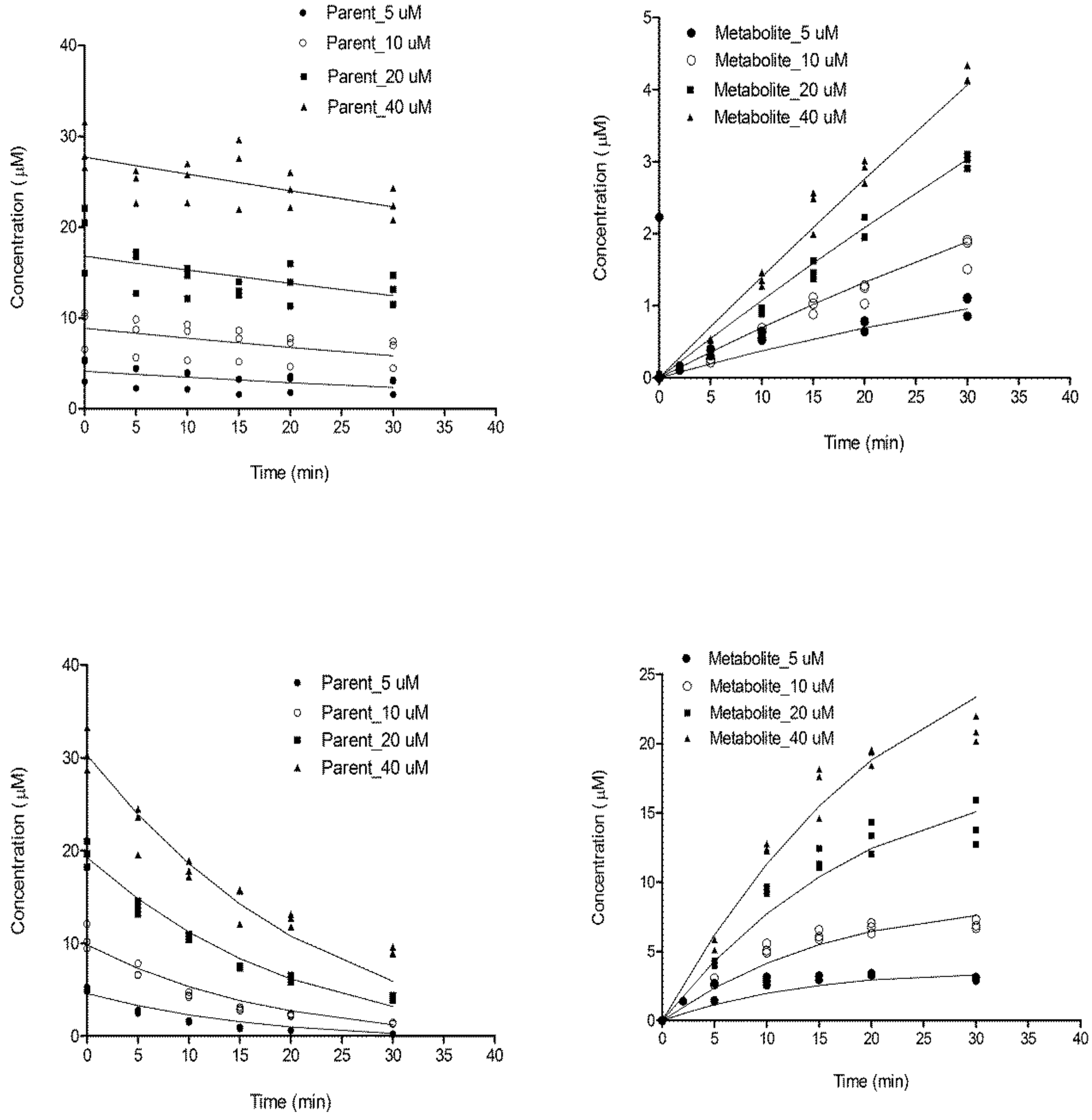

Supplementary Figure 10: **Rat blood PK profile for compound 12.**

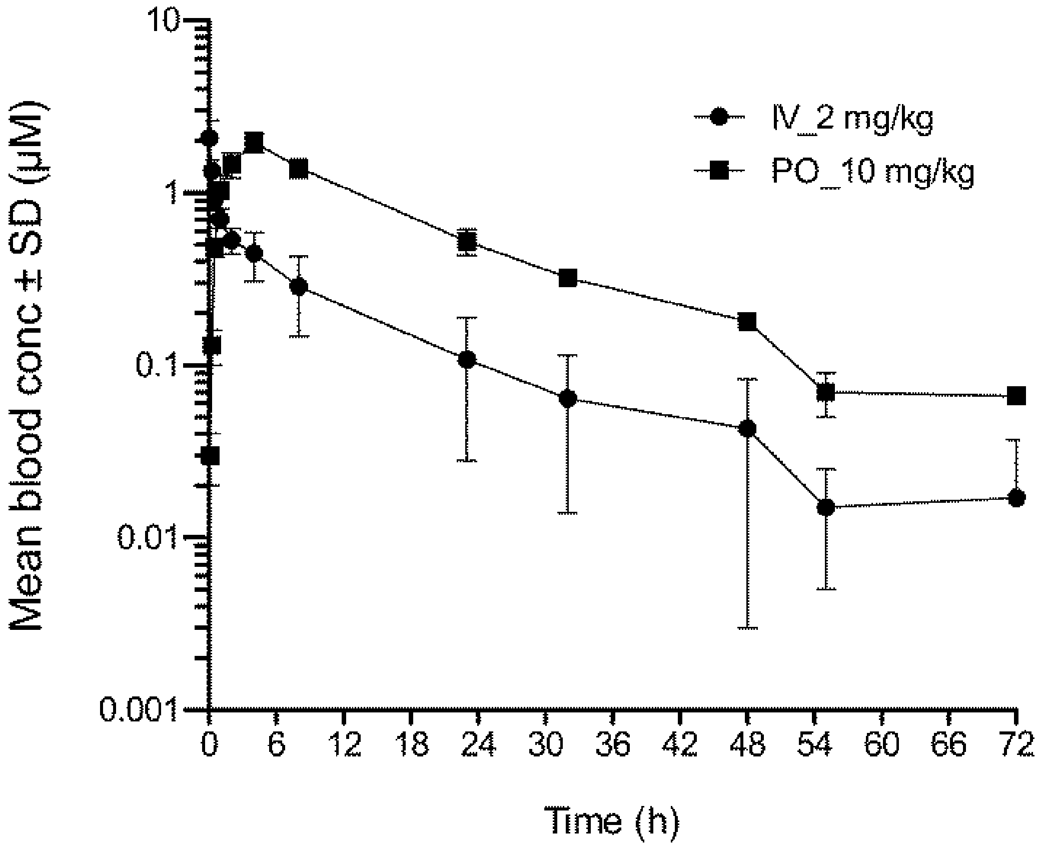

Supplementary Figure 11: **Dog plasma PK profile for compound 12.**

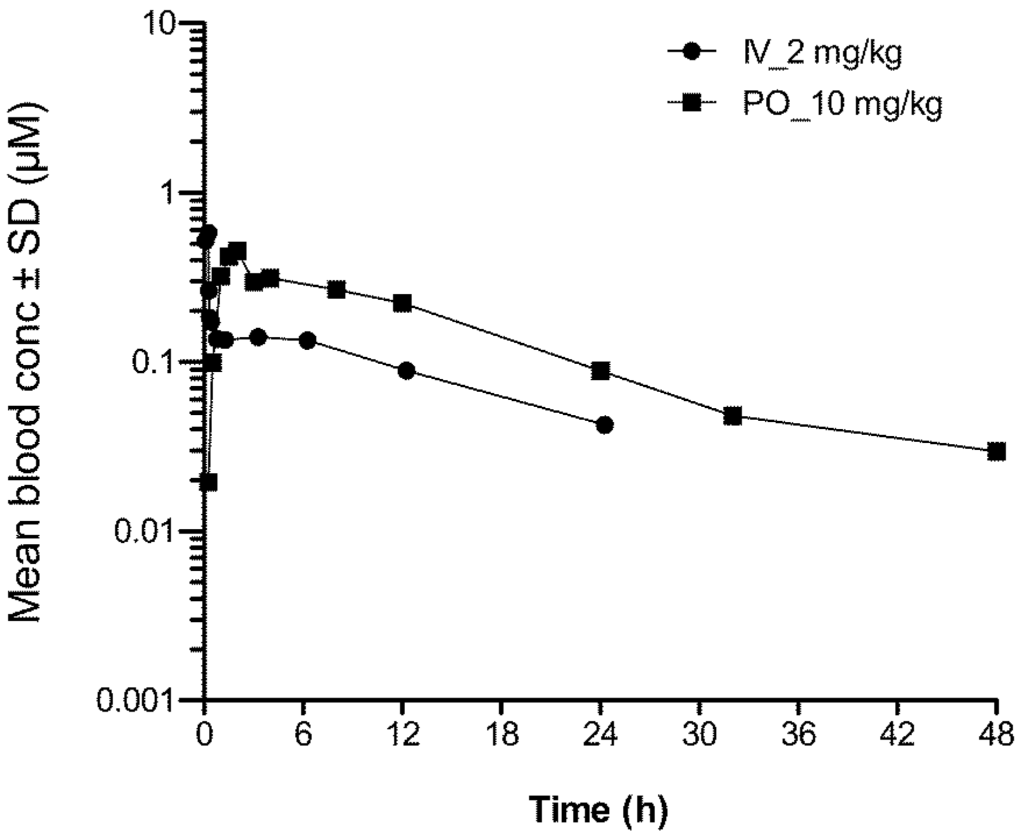

Supplementary Figure 12: Predicted human blood PK profile.

Predicted human POPK profile with dotted line indicating the minimum parasiticidal concentration (MPC) of 0.04 μM.

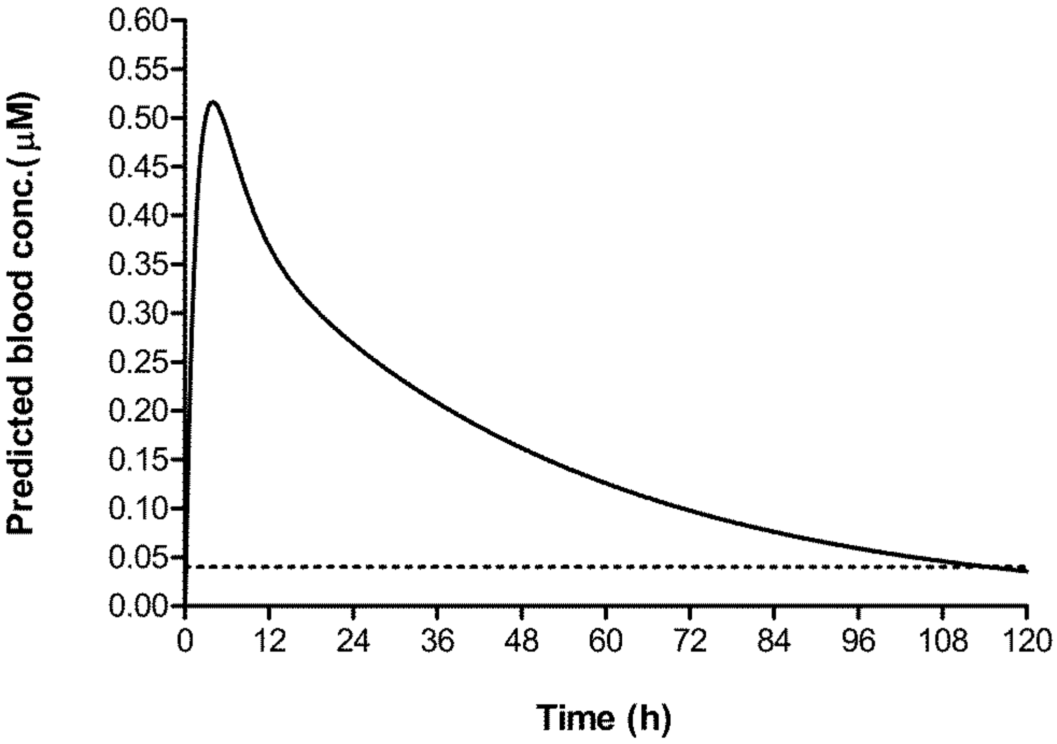

Supplementary Figure 13: Prediction of human efficacy

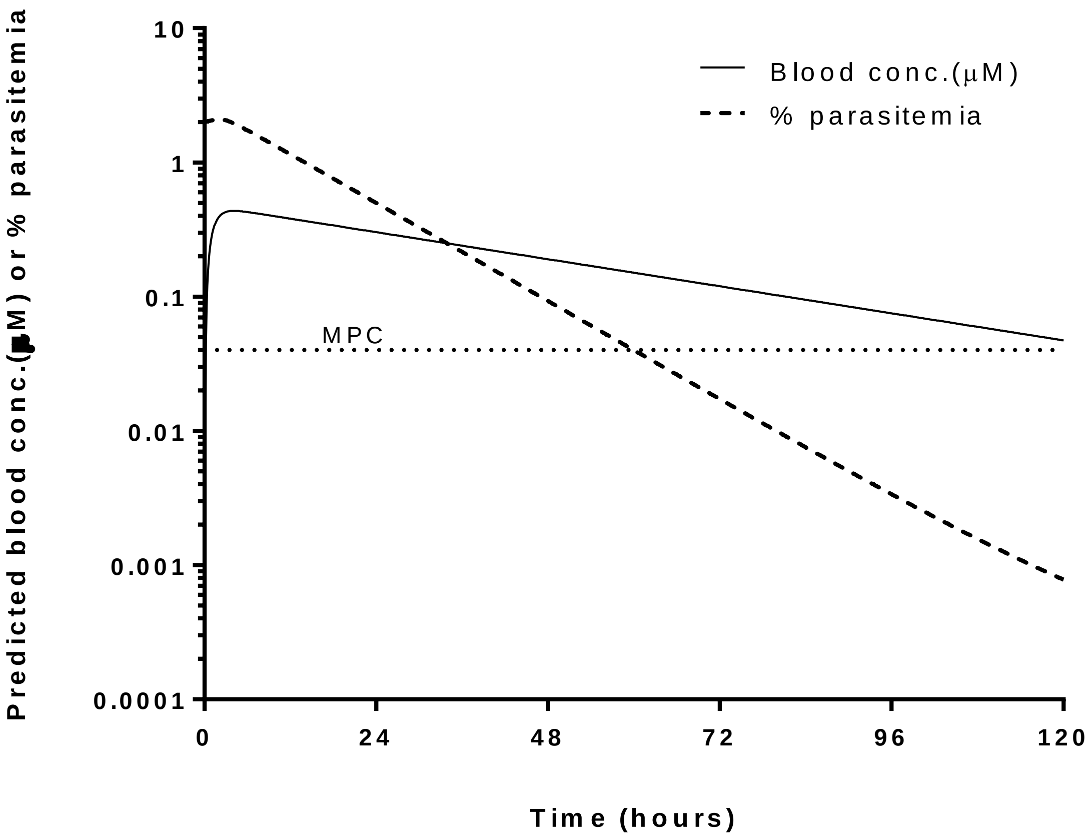

Supplementary Figure 14:

T-Coffee alignment using homologues of PF13_0227. This alignment was built using homologues of PF13_0227 of *Saccharomyces cerevisiase* (Vma8p), *Homo sapiens* (AAH31002) and *Thermus thermophilus* (PDB structure 3W3A_G). The consensus sequence shows conserved amino acids in upper case and marked in red. The top row of each alignment shows alpha-helical regions found in the crystal structure. The shared mutation identified in all drug-pressured mutants is marked in blue. T-Coffee graphical viewer ESPript/ENDscript has been described earlier[1].

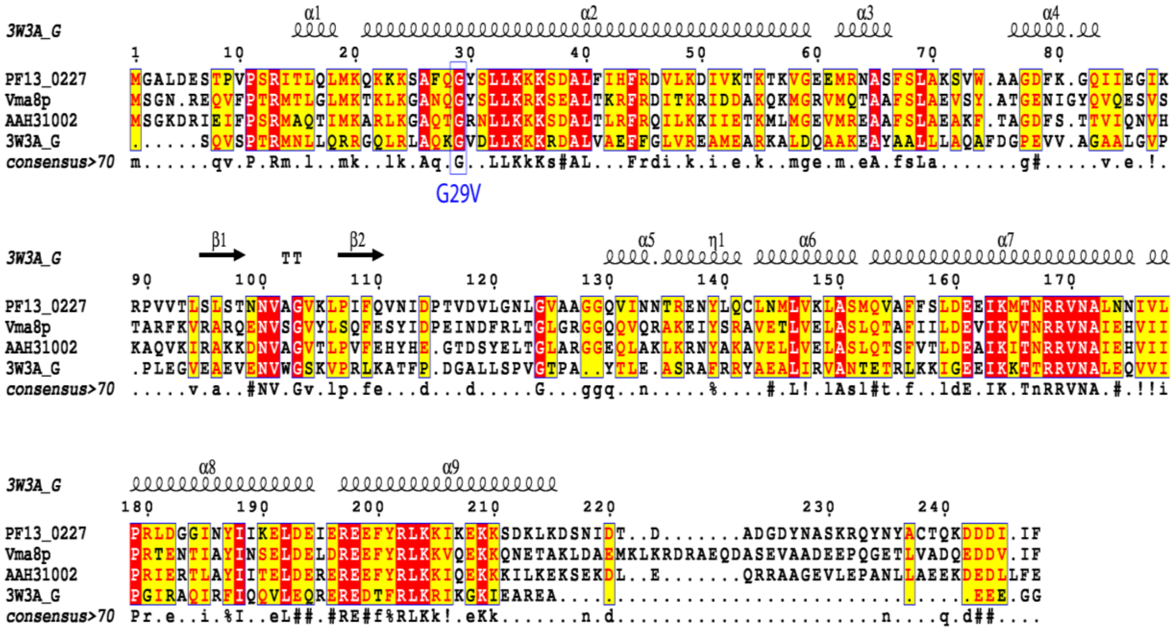

Supplementary Figure 15:

Determination of vacuolar sizes in compound 6-resistant mutants. Vacuolar and cytoplasmic size determination of compound **6**-resistant parasite harbouring the G29V mutation in the V-type ATPase subunit D gene. Sorbitol-synchronized parasites were methanol-fixed and Giemsa stained and images taken using a 100x objective mounted on an Olympus CX41 bright field microscope with a colour camera attachment. The areas of vacuoles and parasites were measured using ImageJ and the statistical analysis using data from 44-80 cells was performed using the R software package (**Supplementary Fig. 15 A**). The size-ratios of clones F2-D6 and F4-D9 were compared to the susceptible, parental strain Dd2-B2 (parent). All data sets (black lines) followed closely an ideal normal distribution (red line, **Supplementary Fig. 15 B**), which was statistically verified by the Shapiro-Wilks test for normality. The unpaired Student's test was used to compare the means from the data of either resistant strain or the parental strain.

A relative vacuole sizes in compound 6-resistant mutants

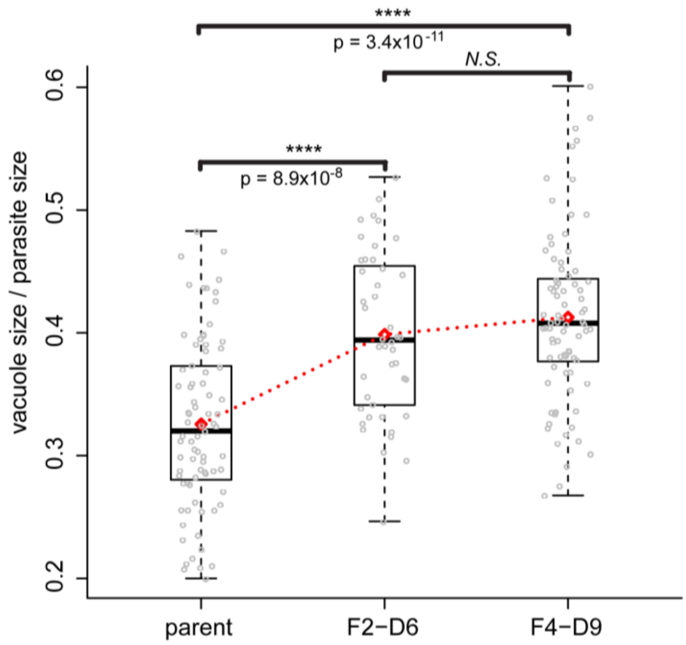

	parent	F2-D6	F4-D9
Number of cells counted (n)	79	44	80
Mean	0.326	0.399	0.413
Standard Deviation	0.068	0.066	0.069
Median	0.320	0.394	0.408

B Distribution of size ratios (vacuole/parasite)

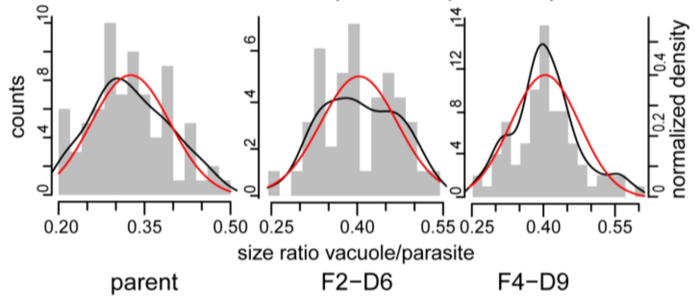

Figure 16a: **Effects of vehicle and compound 12 on systolic arterial blood pressure**

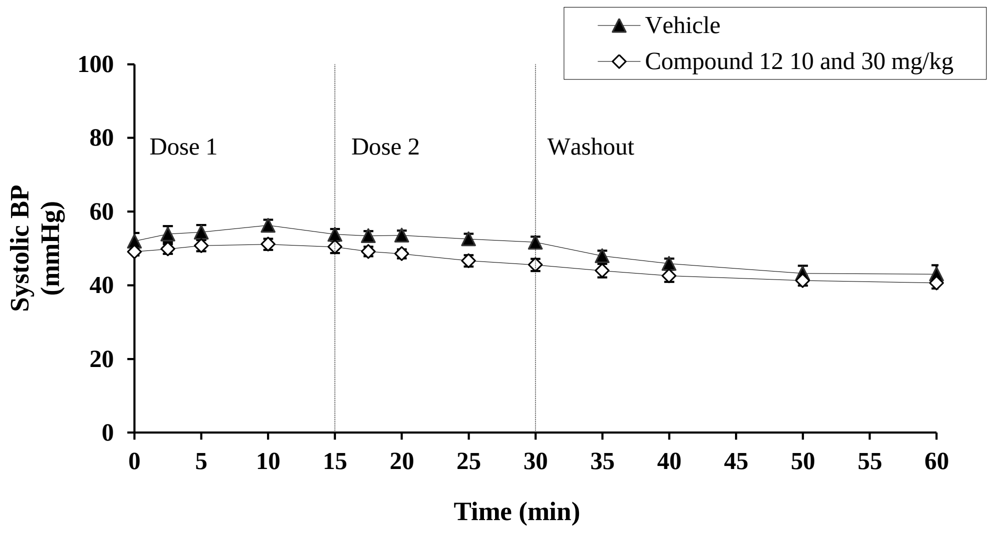

Figure 16b: **Effects of vehicle and compound 12 on diastolic arterial blood pressure**

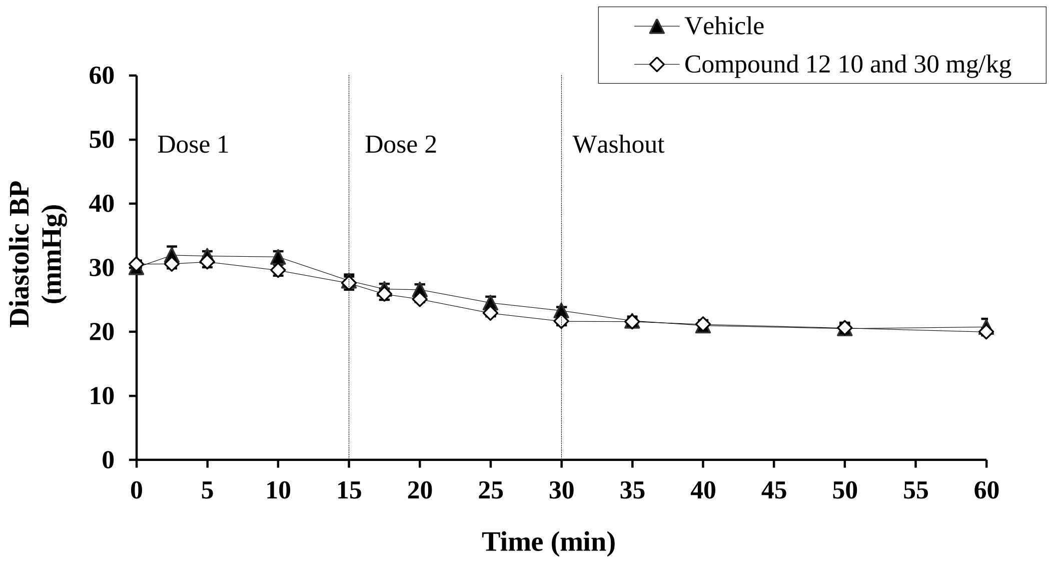

Figure 16c: Effects of vehicle and compound 12 on mean arterial blood pressure

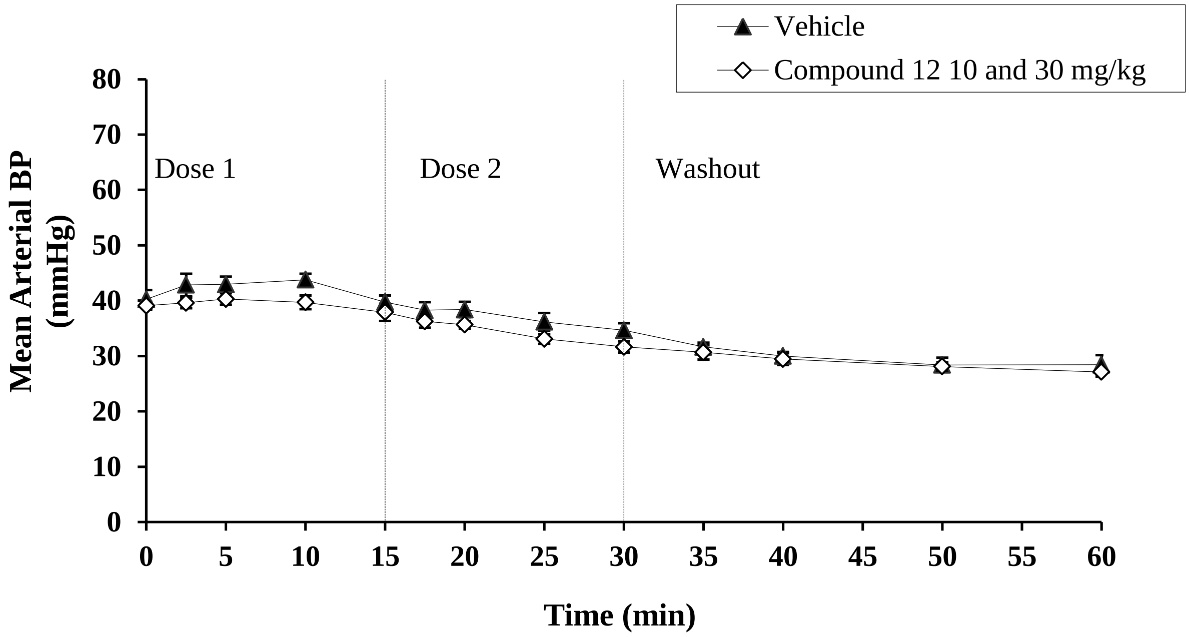

Figure 16d: Effects of vehicle and compound 12 on heart rate

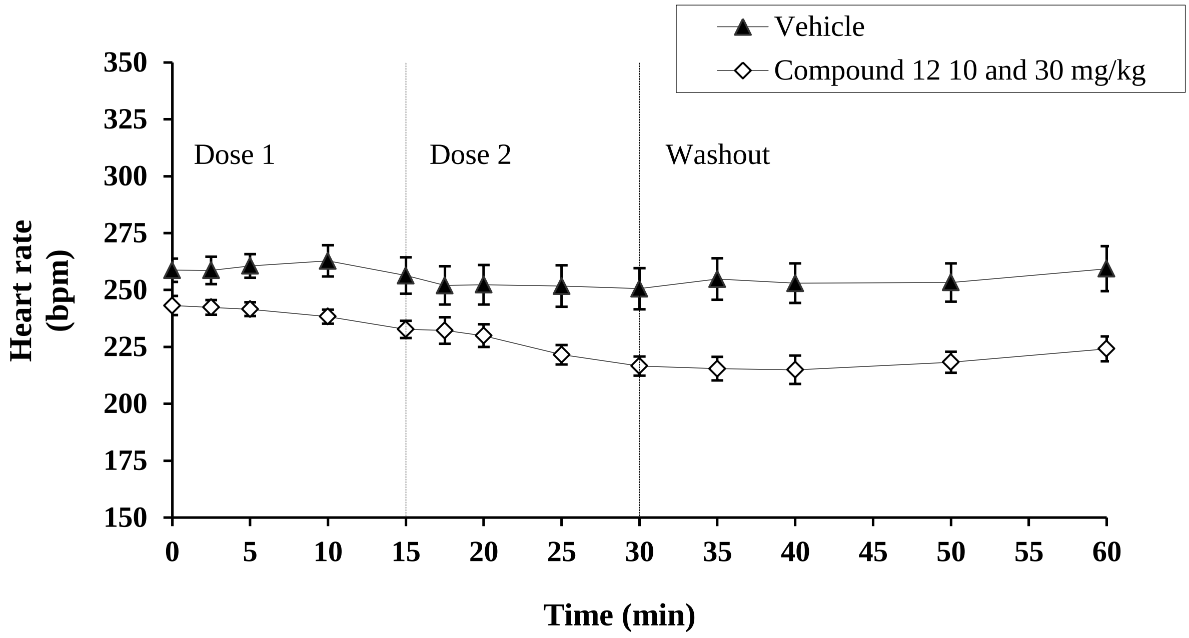

Figure 16e: Effects of vehicle and compound 12 on PR interval

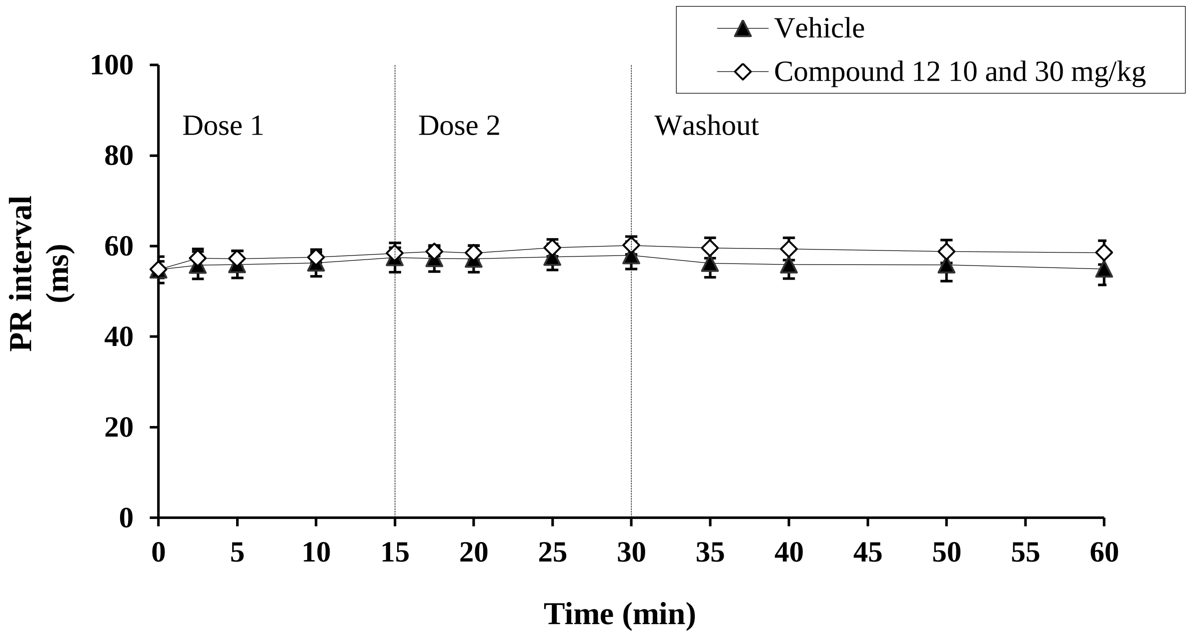

Figure 16f: Effects of vehicle and compound 12 on QRS duration

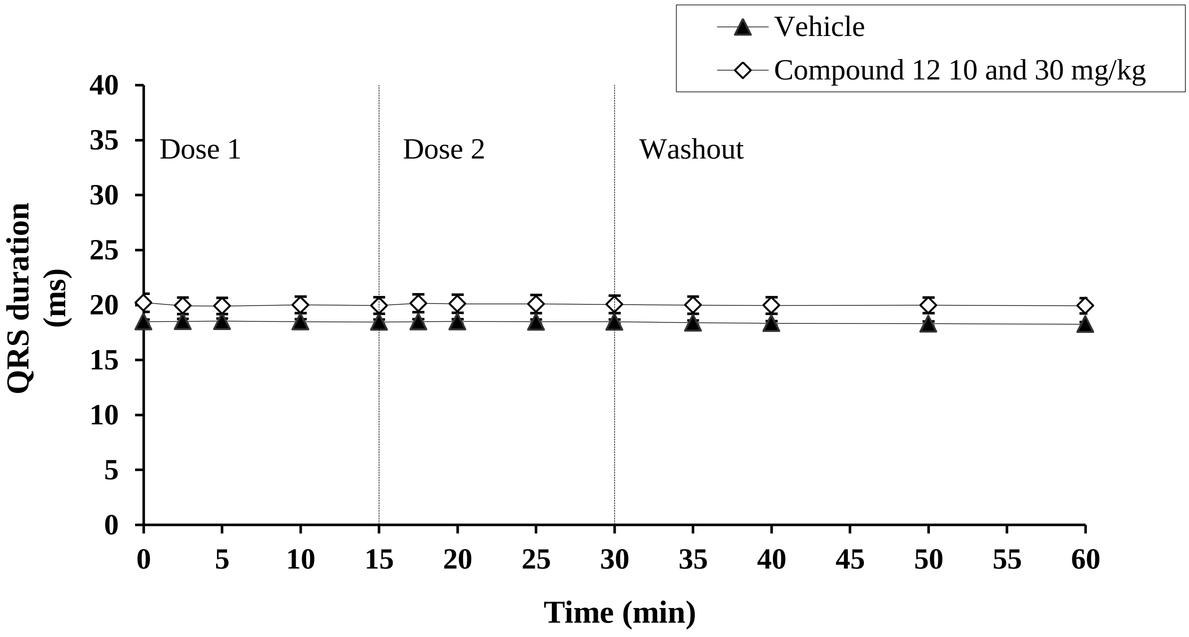

Figure 16g: **Effects of vehicle and compound 12 on QT interval**

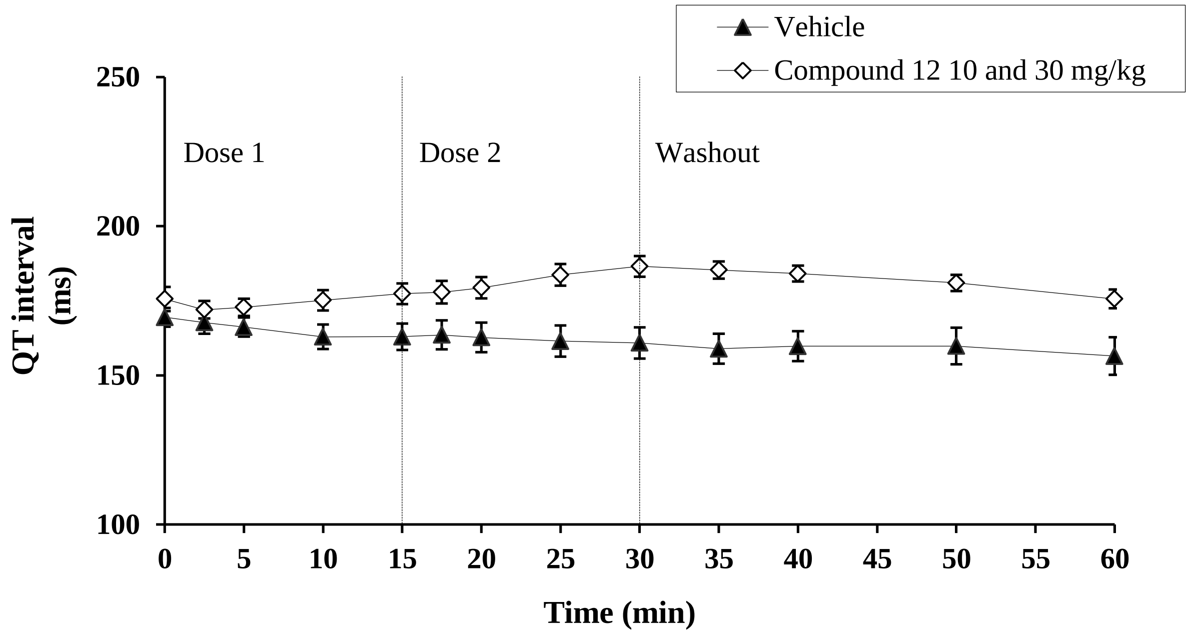

Figure 16h: **Effects of vehicle and compound 12 on QTcB interval**

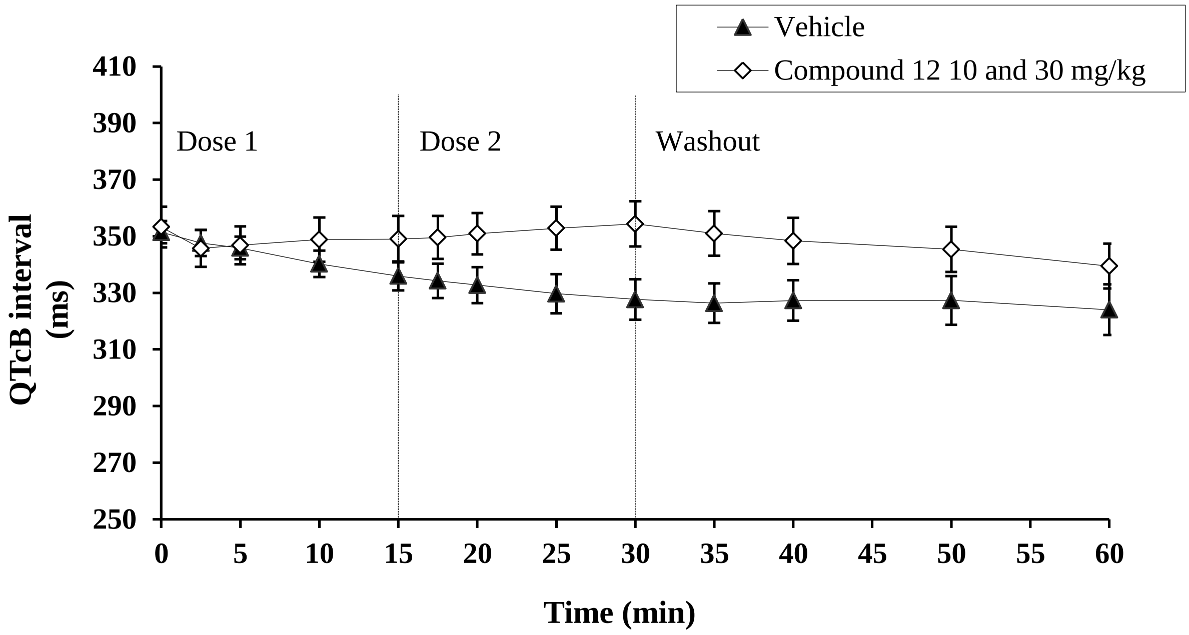

Figure 16i: Effects of vehicle and compound 12 on left ventricular systolic blood pressure

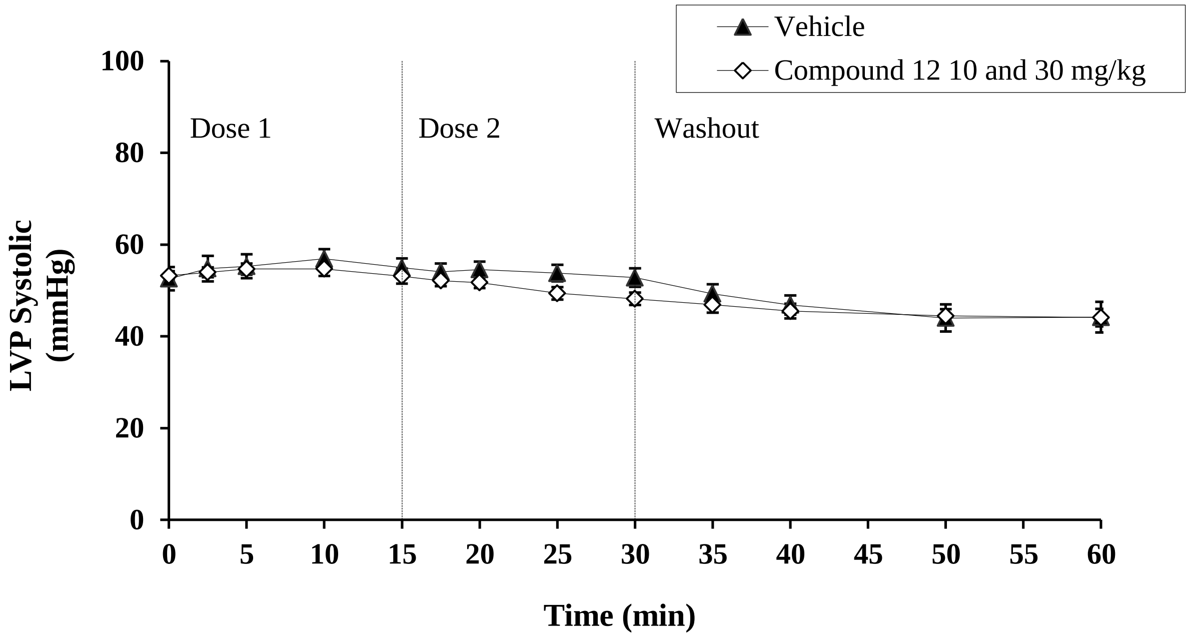

Figure 16j: **Effects of vehicle and compound 12 on left ventricular end diastolic pressure**

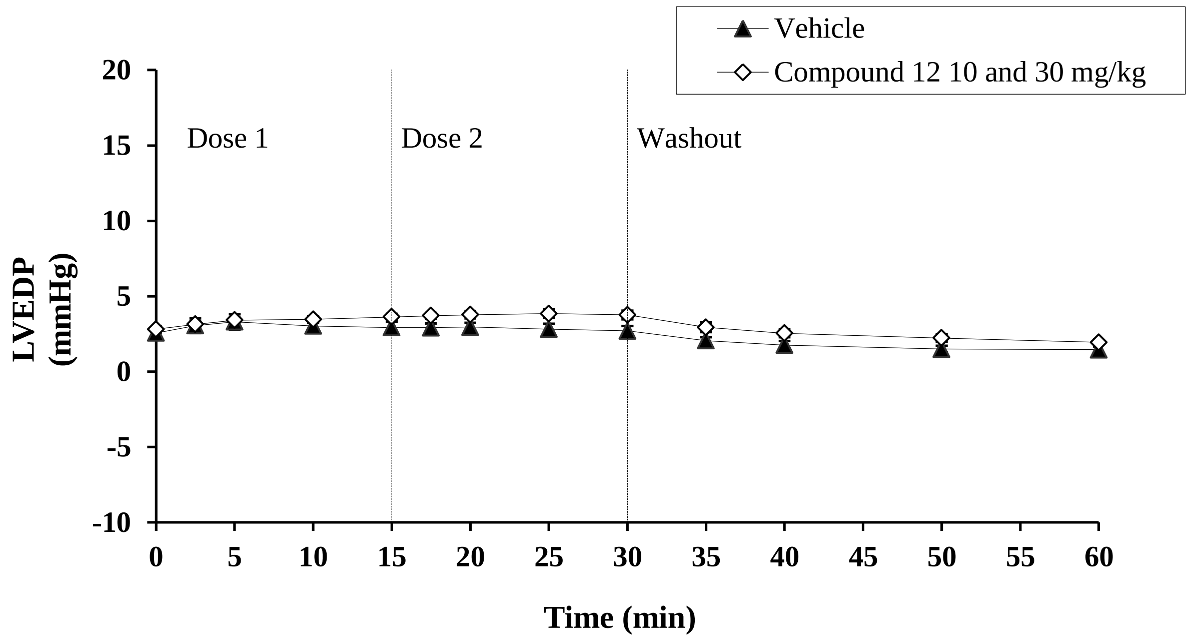

Figure 16k: **Effects of vehicle and compound 12 on dP/dtmax**

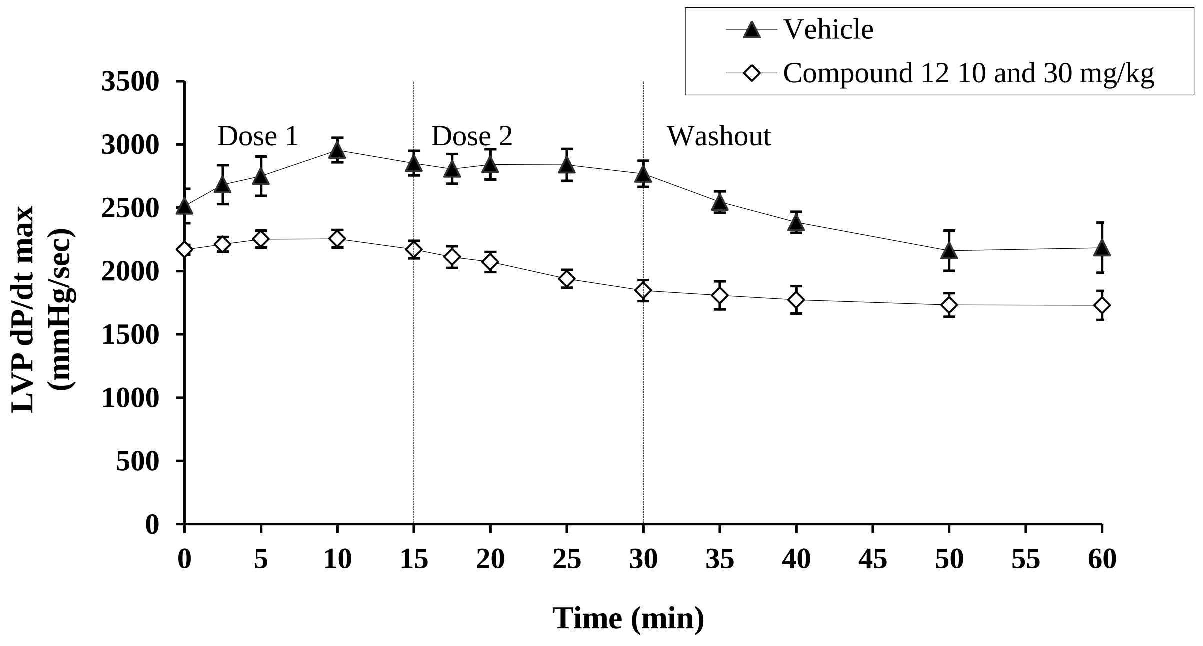

Figure 16l: Effects of vehicle and compound 12 on dP/dtmin

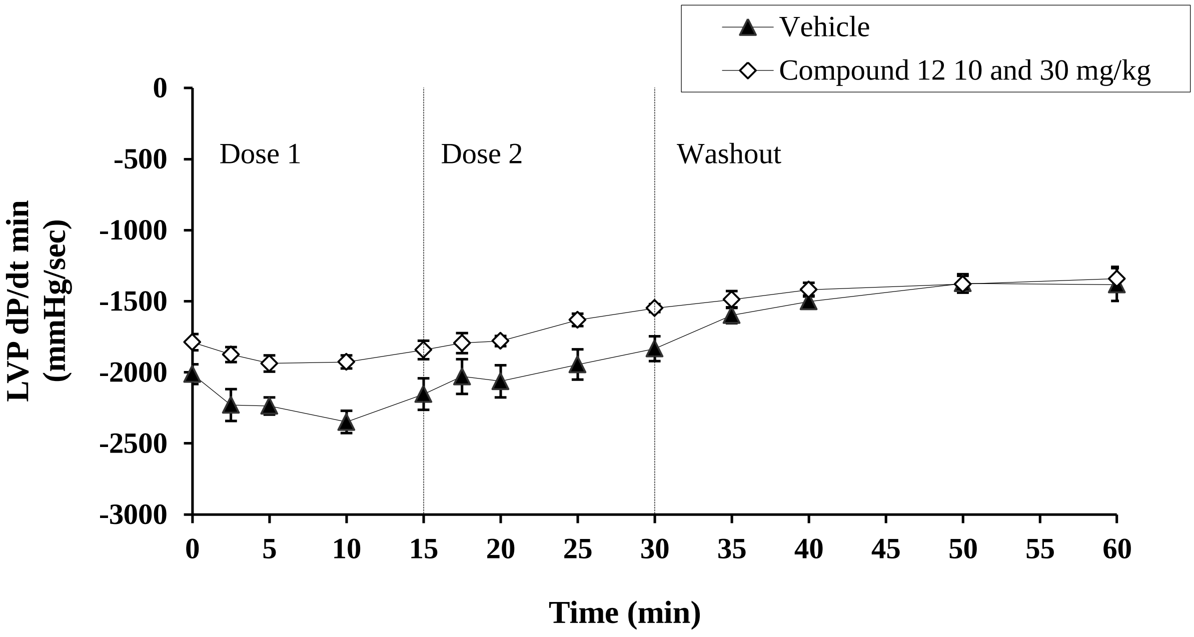

Figure 16m: Total plasma concentrations of compound 12 (Mean ± SEM)

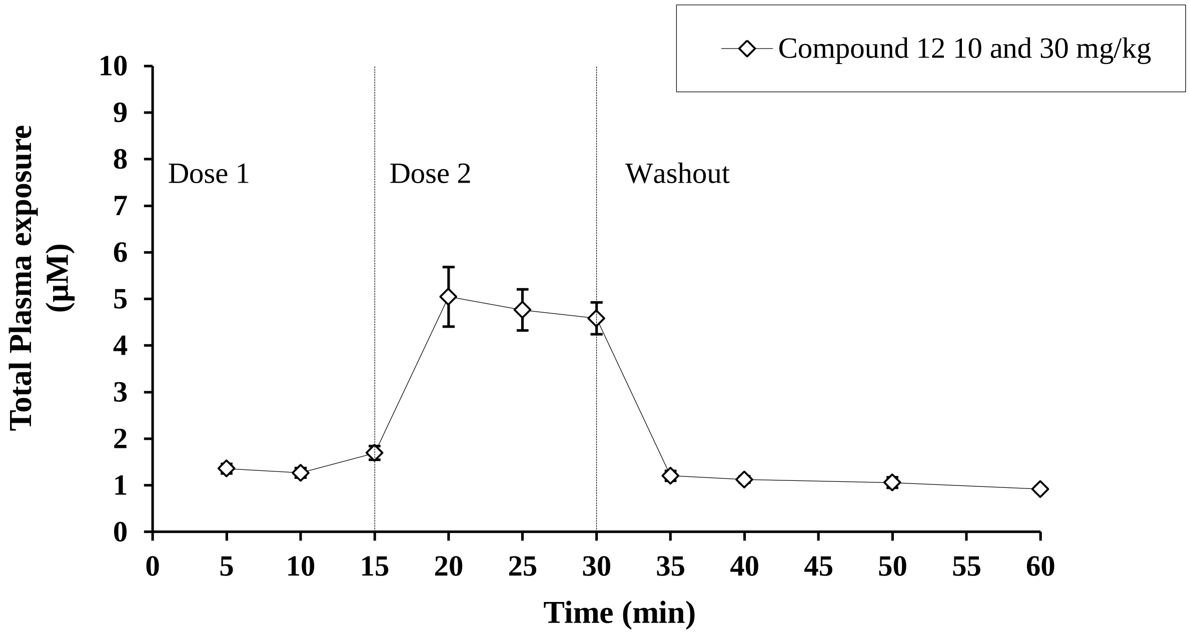

Supplementary Table 1:

TAPs retain their activity against a panel of *Pf* strains resistant to antimalarial agents in clinical use and in preclinical development. TAPs retain their antiplasmodial activity against a panel of *Pf* strains known to harbour mutations conferring resistance to known antimalarial drugs in clinical use. Results show the IC_{50} values obtained for each compound tested in duplicate from two independent experiments using a SYBR-Green based assay[2].

	IC_{50} (nM)					
**Compound**	**NF54**	**K1**	**7G8**	**W2**	**SB1**	**DD2**
5	60.0 ± 18	60.0 ± 18	75.2±11	81.9±0.09	29.1±3	58.1±20
8	57.2 ± 20	64.7 ± 12	74.4±17	78.8±7	34.7±3	38.3±1.5
9	18.5 ± 5	33.8 ± 5	26.5±5	28.06±8	25.6±13	19.6±7
Artemisinin	7.0 ± 4	6.5 ± 2	1.3±0.1	6.5±1	16.6±5	10.3±2
Chloroquine	17.1 ± 5	347.3 ± 82	163.9±17	374.7±228	11.8±4.5	251.3±9
Pyrimethamine	28.3 ± 11	8558.4 ± 1983	10041.7±1261	15379.7±2826	10.8±4	13838.6±8619
Atovaquone	1.4 ± 0.4	2.3 ± 0.8	2.3±0.7	1.2±0.6	>2000	2±0.02

Supplementary Table 2: Compound 6 retains activity against a panel of *Pf* strains harbouring mutations to novel antimalarial agents in development.

A representative TAP (compound **6**) was tested for its activity against a panel of *P. falciparum* strains known to harbour mutations conferring resistance to antimalarial agents under development. Artesunate and amodiaquine were included as additional control compounds showing no significant difference in IC_{50} values among all of the strains tested. Values are the means ± standard deviation derived from three independent assays performed in triplicate. IC_{50} values of control compounds against their specific resistant-mutant strain are highlighted in bold.

Compound	IC_{50} (nM)				
	Dd2	Dd2-DHODH	Dd2-HSP90	Dd2-PfCRT	Dd2-CYTbQi
**6**	29.53 ± 3	23.22 ± 5	27.9 ± 7	18.95 ± 1	25.73 ± 5
**Artesunate**	1.51 ± 0.2	1.81 ± 0.3	1.29 ± 0.2	1.84 ± 0.3	1.26 ± 0.4
**Amodiaquine**	9.83 ± 0.4	10.74 ± 2	8.96 ± 0.8	8.86 ± 1	9.9 ± 3
**Genz-669178**	5.79 ± 1	**76.8 ± 10**	16.2 ± 2	5.07 ± 4	9.94 ± 0.9
**Geldanamycin**	176 ± 30	257 ± 40	**658 ± 30**	204 ± 20	169 ± 40
**IDI-3783**	7.30 ± 7	16.9 ± 9	8.42 ± 1	**1045 ± 700**	3.16 ± 0.2
**IDI-5918**	2.60 ± 0.6	2.37 ± 0.5	ND	1.95 ± 1*	**618 ± 90**

*value reflects mean of two replicate assays ± standard deviation; ND = not determined.

Compound **6** does not show significant differences in IC_{50} in the strains assayed. Artesunate and amodiaquine, both with unrelated modes of action from the targets tested, do not show significant differences in IC_{50} values across all strains. Control compounds for each of the resistant mutants demonstrate expected phenotypes. The *Pf* DHODH mutant is resistant to Genz-669178, the *Pf* HSP90 mutant is resistant to Geldanamycin, the *Pf* CRT mutant is resistant to IDI-3783, and the *Pf* CYTb-Qi mutant is resistant to IDI-5918.

Supplementary Table 3: Compound 9 and 12 retain their activity against Pf strains harbouring mutations to novel antimalarial agents in clinical development. Compound **9** and **12** were tested for their antiplasmodial activity against a panel of *Pf* strains known to harbour mutations conferring resistance to novel agents under clinical development. Parasite growth inhibition is measured using the [^{3}H]-hypoxanthine incorporation assay. IC_{50}s are established for each strain (n ≥2) and compared to the IC_{50} of Dd2 (standard pan-resistant strain).

Compound	IC $_{50}$ (nM)							
	Dd2	Dd2 bc-1 ELQ-300	Dd2 PI4K Cpd 1	Dd2 DHODH DSM265	Dd2 PfATP4 NITD609	PfATP4 Cpd 2	PfATP4 Cpd 3	Cpd 4 Unpublished Target
ELQ-300	20	125						
PI4K Cpd 1	5.2		32					
DHODH DSM265	4.3			48				
PfATP4 NITD609	0.6				1.7			
PfATP4 Cpd 2	6.1					116		
PfATP4 Cpd 3	43						3194	
Cpd 4 Unpublished Target	6.9							803
Cpd 9	6	10	10	9.1	8.6	11	11	9.4
Cpd 12	3.5	8.2	7.1	6.5	6.8	8.1	10	8.2

Supplementary Table 4: The pharmacokinetic parameters for compound 9 estimated by fitting a one compartment POPK model.

Parameter	Unit	Estimate	% CV
Dose	mg kg^{-1}	10	
V/F	L kg^{-1}	70	37
Ka	h^{-1}	0.29	46
CL/F	L h^{-1} kg^{-1}	7.3	12
Dose	mg kg^{-1}	30	
V/F	L kg^{-1}	53	9
Ka	h^{-1}	0.62	13
CL/F	L h^{-1} kg^{-1}	7	5

Supplementary Table 5: The parameters estimated by modelling of the PKPD data for compound 9.

Parameter	Unit	Estimate	% CV
K_{gro}	h^{-1}	0.024	7
K_{kill}	h^{-1}	0.2	29
EC_{50}	µM	0.309	20

Supplementary Table 6: PK-PD parameters for the parent and metabolite in the *Pf*/SCID model.

PK parameters for the parent and metabolite (Ka, CL/F, Vc/F, Ke_0, CL_m, Vc_m, Ke_{om}) as well as PD parameters for the metabolite (EC_{50m}, H and K_{killm} estimated from the PK-PD model fit to the metabolite efficacy in the *Pf* SCID model) were incorporated (as ‘Fixed’) in the parent plus metabolite PK-PD model. The fixed parameter estimates and the model predicted estimates of K_{kill} and EC_{50} are shown in the table below.

Parameter	Unit	Estimate	% CV
N_{max}	--	12	Fixed
K_{gro}	h^{-1}	0.032	Fixed
EC_{50m}	µM	0.3	Fixed
K_{killm}	h^{-1}	0.2	Fixed
H	--	3	Fixed
Ka	h^{-1}	1	Fixed
CL/F	$L\ h^{-1}\ kg^{-1}$	39	Fixed
Vc/F	$L\ kg^{-1}$	462	Fixed
Ke_0	h^{-1}	0.06	Fixed
CL_m	$L.h^{-1}.kg^{-1}$	80	Fixed
Vc_m	$L\ kg^{-1}$	29	Fixed
Ke_{0m}	h^{-1}	0.06	Fixed
K_{kill}	h^{-1}	0.097	2.6

EC_{50}	µM	0.038	4.3

Supplementary Table 7: Parameters estimated by fitting the Michaelis-Menten model.

Parameter	Unit	Estimate	% CV
HLM estimates			
V_{max}	µM min^{-1}	0.258	53
Km	µM	22.490	93
MLM estimates			
V_{max}	µM min^{-1}	4.836	31
Km	µM	78.339	38

Supplementary Table 8: Rat blood PK parameters for compound 12.

		IV				Oral			
Parameter	Units	Rat 1	Rat 2	Rat 3	Mean	Rat 1	Rat 2	Rat 3	Mean
Dose	mg kg^{-1}	2	2	2	2	10	10	10	10
CL	mL $min^{-1}.kg^{-1}$	13.1	4.8	9.6	9.2				
Vss	$L.kg^{-1}$	9.3	6.6	7.2	7.7				
MRT	H	11.8	22.6	12.6	15.7				
t½	H	13.5	19.2	13	15.2				
F	%					121	52	96	90
C_{max}	µM					1.712	2.206	1.993	2
T_{max}	H					4	4	4	4
$AUC_{0\text{-}inf}$	µM h					33.1	38.4	36	35.8
$AUC_{0\text{-}t}$	µM h					31.8	37.2	35.1	34.7

Supplementary Table 9: Dog plasma PK parameters for compound 12.

Dog blood PK parameters were estimated from plasma PK parameters assuming a blood-plasma (BP) ratio of 2.0 in dog.

		IV			Oral		
Parameter	Units	Dog 1	Dog 2	Mean	Dog 1	Dog 2	Mean
Dose	mg kg^{-1}	2.1	2.1	2.1	5.1	5.1	5.1
CL	mL min kg^{-1}	27.5	21.3	24.4			
Vss	L kg^{-1}	24.9	20.4	22.7			
MRT	H	15.1	15.9	15.5			
t½	H	11	11	11	15.2	17	16.1
F	%				88.1	96.4	92.3
C_{max}	µM	0.641	0.515	0.578	0.3	0.603	0.452
T_{max}	H				2	2	2
$AUC_{0\text{-}inf}$	µM h	2.72	3.47	3.095	5.87	8.28	7.075
$AUC_{0\text{-}t}$	µM h	2.16	2.68	2.42	5.27	7.48	6.375

Supplementary Table 10: ***In vitro*** **Clint, Plasma protein binding (PPB), Blood-plasma ratio (BP ratio), observed and predicted blood CL based on IVIVC/IVIVE.**

**Parameter**	**Unit**	**Mouse**	**Rat**	**Dog**	**Human**	**Guinea pig**
Hepatocyte Clint	(µl.min^{-1}.10^{5})	ND	11.3	9.3	3.1	ND
B:P ratio		1.2	1.65	1.35	1.3	ND
Plasma protein binding	Plasma fu (% free)	0.005 (0.55 % free)	0.04 (4.0 % free)	0.038 (3.8 % free)	0.025 (2.5 % free)	0.055 (5.5 % free)
Predicted blood CL	(ml.min^{-1}.kg^{-1}) / % LBF	ND	18.8 / 26%	20.8 / 38%	3.8 / 24%	ND
Observed blood CL	(ml.min^{-1}.kg^{-1}) / % LBF	48-53 / 32-35%	5-13 / 7-18%	19 / 33%	NA	ND

Supplementary Table 11: Allometry parameters for compound 12.

	a (coefficient)	B (exponent)
CL	0.6	1.08
Vss	180.1	1.23

Supplementary Table 12: **Predicted human PK parameters.**

Parameter	Estimate
Dose	260 mg / 70 kg
Dose	8 µmol kg^{-1}
CL	0.26 L h^{-1} kg^{-1}
Vc	4.0 L kg^{-1}
CLd	1.2 L h^{-1} kg^{-1}
Vt	7.3 L kg^{-1}
Ka	0.3 h^{-1}
F	0.7

Supplementary Table 13: Resistance profile of TAP-resistant mutants.

In vitro selection of compound **6** resistance resulted in a 3-6 fold increase in IC_{50} values for **6** and **12** compared with the parental Dd2 line, but not to other antimalarial agents. Values are the means ± standard deviation derived from three independent assays performed in triplicate, and are shown for five independent clones (B5, C5, D8, D4 and G7) from two independent selections (F2 and F4).

Compound	IC_{50} (nM)					
	Dd2	Dd2-6^R F2 B5	Dd2-6^R F2 C5	Dd2-6^R F2 D8	Dd2-6^R F4 D4	Dd2-6^R F4 G7
**6**	38.7 ± 1.1	102.5 ± 11.2	96.3 ± 17.0	95.7 ± 17.6	98.9 ± 26.4	112.0 ± 27.4
**12**	16.1 ± 1.0	81.0 ± 17.9	70.1 ± 17.0	69.6 ± 21.8	81.6 ± 38.5	90.7 ± 37.9
**Artesunate**	2.6 ± 1.3	1.6 ± 0.2	1.2 ± 0.1	1.4 ± 0.1	1.6 ± 0.9	1.6 ± 0.2
**Mefloquine**	30.1 ± 7.2	11.7 ± 2.6	12.1 ± 1.2	12.0 ± 1.2	11.7 ± 2.5	10.7 ± 1.2
**Lumefantrine**	15.6 ± 4.8	6.8 ± 2.0	5.7 ± 1.0	6.0 ± 1.7	6.4 ± 2.4	5.1 ± 1.3

Compound **6** showed a 3-fold increase in IC_{50} compared with the parental Dd2 strain. Dd2-6^R clones are cross-resistant with compound **12** but not with standard antimalarials tested, indicating that resistance developed specifically for TAPs. The Dd2-6^R clones show increased sensitivity to artesunate, mefloquine and lumefantrine compared with Dd2, probably due to reduced copy number of the multidrug resistance protein 1 (PfMDR1)[3] in these clones (from 3 copies in Dd2 to 2 copies in the clones, as evidenced from our whole-genome sequence analysis). This phenomenon has been observed in another unrelated drug resistance selection using the Dd2 parental line (data not shown) and is consistent with the known variation over time *in* pfmdr1 copy number that can occur in cultured parasites[4].

Supplementary Table 14: Results from a comparative analysis of next-generation sequencing data from two independent resistance selection experiments.

chromosome	position	ref. nt	alt. nt	NGS data §	amino acid exchange	gene name	former name	function	manual annotation of the mutant position
polymorphisms identified in all clones from both selection flasks:									
Pf3D7_13_v3	1650402	C	A	F2_C5 / F2_B5 / F2_D8 / F4_D4 / F4_G7 / F4_bulk*	G29V	PF3D7_1341900	PF13_0227	vacuolar ATP synthase subunit D, putative	-
polymorphisms identified in all clones from selection flask 2:									
Pf3D7_03_v3	497151	C	T	F2_C5 / F2_B5 / F2_D8	W71*	PF3D7_0311500	PFC0486c	conserved Plasmodium protein, unknown function	protein 81 aa long, with repeat
Pf3D7_14_v3	2680179	G	A	F2_C5 / F2_B5 / F2_D8	S5727N	PF3D7_1465800	PF14_0626	dynein beta chain, putative	-
polymorphisms identified in individual clones:									
Pf3D7_06_v3	549835	T	A	F2_C5 / F2_D8 / F4_D4 / F4_G7 / F4_bulk*	N846I	PF3D7_0613500	PFF0655c	adapter-related protein, putative	in repeat region
Pf3D7_06_v3	509327	A	T	F4_D4	Y602N	PF3D7_0612100	PFF0590c	eukaryotic translation initiation factor 3 subunit L, putative	in N-repeat
Pf3D7_09_v3	929365	T	G	F4_D4	N1899H	PF3D7_0922800	PFI1120c	conserved Plasmodium protein, unknown function	polymorphic in parent
Pf3D7_09_v3	929368	T	G	F4_D4	N1898H	PF3D7_0922800	PFI1120c	conserved Plasmodium protein, unknown function	polymorphic in unrelated parent
Pf3D7_12_v3	1216381	G	C	F4_D4	N445K	PF3D7_1229600	PFL1430c	conserved Plasmodium protein, unknown function	in NK-repeat
Pf3D7_13_v3	372396	G	C	F2_C5	S46T	PF3D7_1308300	PF13_0045	40S ribosomal protein S27, putative	-
Pf3D7_13_v3	474828	T	C	F4_D4	N59D	PF3D7_1310800	MAL13P1.52	conserved Plasmodium protein, unknown function	in N-repeat
Pf3D7_14_v3	696323	A	G	F4_D4	N580S	PF3D7_1417200	PF14_0170	NOT family protein, putative	polymorphic in unrelated parent

* Next-generation sequencing data were retrieved from the selected flask 4 bulk culture parasites prior to cloning by limiting dilution.

§ some calls were removed from the list due to their occurence in unrelated, unpressured Dd2 parasites or were found to be commonly mutated in unrelated selection studies.

Supplementary Table 15: Secondary Pharmacology profiles of compounds 2 & 12

The secondary pharmacology profile of compound **2** and **12** against a panel of 25 diverse targets (8 enzymes, 5 ion channels, 10 GPCRs and 2 transporters) and hERG. The identity of majority of targets is not disclosed as this is currently proprietary AstraZeneca information. For assessment of activity against these targets, compounds were tested in an 8-point concentration response covering half-log units up to a top test concentration of 100 µM, and an IC_{50} or Ki determined using either radio ligand binding assays or enzyme activity assays.

Target #	Target/Class	Binding Ki / Enzyme IC_{50}	
		**2**	**12**
1	α_{1A} adrenoceptor	1.46	0.6045
2	Ion channel	0.933	2.62
3	AChE	0.179	78
4	Muscarinic M_2 receptor	1.1	1.0515
5	Ion channel	3.64	8.58
6	GPCR	2.695	7.82
7	Transporter	17.1	3.85
8	GPCR	4.735	>100
9	GPCR	>100	2.21
10	GPCR	8.42	>100
11	Enzyme	>100	4.29
12	GPCR	30.45	10.9
13	GPCR	38.7	11.5
14	Transporter	31.45	10.1
15	Enzyme	13	11.6
16	Ion channel	33.2	>100
17	Ion channel	59	>100
18	hERG	85.02	35.85
19	Enzyme	>100	14.5
20	GPCR 9	>100	>100
21	Ion channel	>100	>100
22	GPCR	>100	>100
23	Enzyme	>100	>100
24	Enzyme	>100	>100
25	Enzyme	>100	>100
26	GPCR	>100	>100

Supplementary Table 16: Preliminary rat toxicology findings for compound 2

Study	Dose (mg/kg)	A - Plasma C_{max} (μM) Total	Summary of clinical signs and effects	B – Efficacious Mouse C_{max} (μM) Total	Safety Margin (A / B)
3-day Rat Tolerability	100 (oral)	8.13	Non-specific toxicity including subdued behaviour & noisy respiration	8.9	None
	300 (oral)	19.18	Premature decedents and non-specific toxicity	8.9	None
Acute Anaesthetised Guinea Pig CVS Model	5 (i.v.)	4.53	Haemodynamic Effects	8.9	None
	15 (i.v.)	20.42	Haemodynamic & ECG Effects	8.9	None

Supplementary Note 1

Synthesis of compound **1-6**, **10** and **11** including their intermediate step compounds.

Synthesis of 5-(4-Methylpiperazin-1-yl) pyrimidine-2, 4(1H, 3H)-dione (Intermediate **1a**).

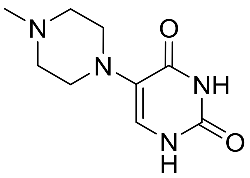

In a 20 mL Biotage microwave vial, 5-Bromouracil (3 g, 15.71 mmol, Aldrich) and N-methylpiperazine (2.61 mL, 23.56 mmol, Aldrich) were taken in pyridine (15 mL) to give a white suspension. The vial was then capped and subjected to microwave irradiation for 45 mins at 150 °C. Solvent was removed under vacuum and the residue was then triturated with ethyl acetate. The resultant suspension was filtered out and dried under vacuum to get 5-(4-methylpiperazin-1-yl)pyrimidine-2,4(1H,3H)-dione (3.30 g, 100 %) as a dark grey solid. Yield: 44%, Purity: >95% by HPLC (UV at 220 and 254 nm). ^{1}H NMR (300 MHz, DMSO-d_6) δ10.84 (br. s., 1H), 7.21 (s, 1H), 3.09-2.98 (m, 4H), 2.49-2.43 (m, 4H), 2.07 (s, 3H). High-resolution mass spectrometry, electrospray ionisation (HRMS (ESI)): M/Z calculated for $C_9H_{14}N_4O_2$ +H [M+H]: 211.1117. Found: 211.1130.

Synthesis of 2, 4-Dichloro-5-(4-methylpiperazin-1-yl)pyrimidine (Intermediate 1b).

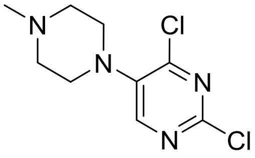

In a 250 mL two neck round-bottomed flask, 5-(4-methylpiperazin-1-yl)pyrimidine-2,4(1H,3H)-dione (Intermediate **1a**, 3.30 g, 15.70 mmol) was taken in phosphorus oxychloride (200 ml) to give a brown suspension. The reaction mass was then heated to 120 °C for 4 h. Solvent was evaporated under vacuum to get a thick dark residue. Ice was added to it and then neutralized with sodium bicarbonate to pH 8 under cooling condition. The suspension was then extracted with 10% methanol in dichloromethane. The combined organic layer was dried over sodium sulphate and then removed under vacuum. The residue was purified by flash chromatography on silica (CH_2Cl_2:MeOH10:1) to get solid of 2, 4-dichloro-5-(4-methylpiperazin-1-yl)pyrimidine (1.1 g, 28.4 %). Yield: 28.4%, Purity: >95% by HPLC (UV at 220 and 254 nm).^{1}HNMR (300 MHz, $CDCl_3$) δ 8.64 (s, 1H), 3.13 (s, 4H), 2.34-2.38 (s, 3H), 2.63-2.52 (m, 4H), high-resolution mass spectrometry, electrospray ionisation (HRMS (ESI)): M/Z calculated for $C_9H_{12}Cl_2N_4$ +H [M+H]: 247.0439. Found: 247.0412

Synthesis of 2-Chloro-5-(4-methylpiperazin-1-yl)-4-(pyrrolidin-1-yl)pyrimidine(Intermediate **1c**)

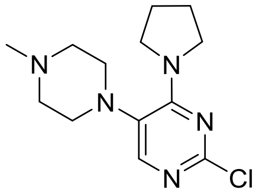

In a 100 mL round-bottomed flask, 2,4-dichloro-5-(4-methylpiperazin-1-yl)pyrimidine (Intermediate **1b**, 15 g, 60.70 mmol) and pyrrolidine (5.57 mL, 66.77 mmol) in ethanol (100 mL) was added DIPEA (15.90 mL, 91.05 mmol) and the reaction mixture was heated at 90°C for 1 h. Solvent was removed under vacuum and then ice cold water was added to get

solid which was filtered out and dried under vacuum to afford the 2-chloro-5-(4-methylpiperazin-1-yl)-4-(pyrrolidin-1-yl)pyrimidine (12.50 g, 73.1 %). Yield: 73.1%, Purity: >95% by HPLC (UV at 220 and 254 nm), high-resolution mass spectrometry, electrospray ionisation (HRMS (ESI)): M/Z calculated for $C_{13}H_{20}ClN_5$ +H [M+H]: 282.1407. Found: 282.1385. Intermediate **1c** was taken for next step without further characterization.

Synthesis of N-(4-Ethylphenyl)-5-(4-methylpiperazin-1-yl)-4-(pyrrolidin-1-yl)pyrimidin-2-amine (**1**)

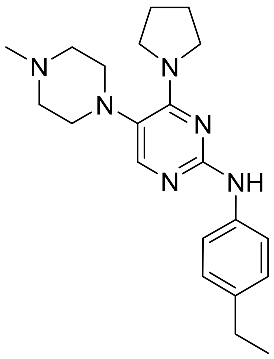

In a 100 mL round-bottomed flask, 2-chloro-5-(4-methylpiperazin-1-yl)-4-(pyrrolidin-1-yl)pyrimidine (Intermediate **1c**, 320mg, 1.14 mmol), 4-ethylaniline (138 mg, 1.14 mmol), $Pd_2(dba)_3$ (52.0 mg, 0.06 mmol), sodium tert-butoxide (218 mg, 2.27 mmol) and Xantphos (65.7 mg, 0.11 mmol) were taken in toluene (2 mL). The resulting reaction mixture was then refluxed under nitrogen for 5 hours. The reaction mixture was passed through celite and washed with ethyl acetate. The combined filtrate was washed with water, dried over sodium sulphate and concentrated under vacuum. The resultant crude product was purified by reverse phase column chromatography to afford N-(4-ethylphenyl)-5-(4-methylpiperazin-1-yl)-4-(pyrrolidin-1-yl)pyrimidin-2-amine (250 mg, 60.1 %). Yield: 60.1%, Purity: >95% by HPLC (UV at 220 and 254 nm). ^{1}HNMR (300 MHz, DMSO-d_6) δ 8.82 (s, 1H), 7.89 (s, 1H), 7.71 (d, J=8.5 Hz, 2 H), 7.10 (d, J=8.48 Hz, 2H), 3.78-3.68 (m, 4H), 3.40 (m, 6H), 2.92-2.79 (m, 4H),

1.99-1.87 (m, 4H) 2.27 (s, 3H)1.21 (t, *J*=7.54 Hz, 3H). ^{13}C-NMR (126 MHz, DMSO-d$_6$) δ 157.02, 156.19, 149.06, 139.35, 135.13, 127.42, 124.96, 117.91, 54.65, 52.33, 48.60. 45.66, 27.46, 24.91 and 15.82, high-resolution mass spectrometry, electrospray ionisation (HRMS (ESI)): M/Z calculated for $C_{21}H_{30}N_6$ +H [M+H]: 367.26043. Found: 367.26015.

Synthesis of 5-(4-Methylpiperazin-1-yl)-N-(2-methylpyridin-4-yl)-4-(pyrrolidin-1-yl)pyrimidin-2-amine (**2**)

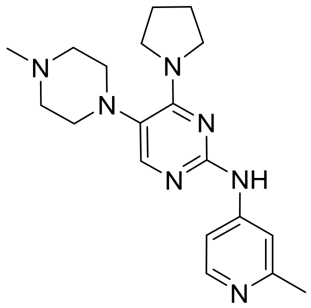

Compound **2** (201 mg, 46 %) was synthesized and purified using procedure analogues to compound **1** from intermediate 1c and 2-methylpyridin-4-amine (Sigma-Aldrich). Yield: 46%, Purity: >95% by HPLC (UV at 220 and 254 nm). 1H NMR (300 MHz, DMSO-*d*$_6$) δ 9.33 (s, 1H), 8.11 (d, *J*=5.84 Hz, 1H), 7.88 (s, 1H), 7.61-7.67 (m, 1H), 7.51 (dd, *J*=5.84, 1.88 Hz,1H), 3.70 (s, 4H), 2.81 (s, 6H), 2.34 (s, 3H), 2.25-2.05 (m, 5H), 1.81-1.96 (m, 4H). ^{13}C-NMR (126 MHz, DMSO-d$_6$) δ 157.41, 156.95, 155.45, 148.66, 148.61, 148.23, 126.19, 110.62, 109.39, 54.50, 52.07, 48.70. 45.75, 24.91 and 24.33, high-resolution mass spectrometry, electrospray ionisation (HRMS (ESI)): M/Z calculated for $C_{19}H_{27}N_7$ +H [M+H]: 354.240043. Found: 354.24003

Synthesis of tert-butyl 4-(2,4-dioxo-1,2,3,4-tetrahydropyrimidin-5-yl)piperazine-1-carboxylate (Intermediate **2a**)

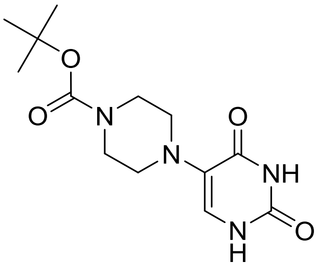

Intermediate **2a** (70 g, 42 %) was synthesized and purified using procedure analogues 1a. Yield: 42%, Purity: >95% by HPLC (UV at 220 and 254 nm), high-resolution mass spectrometry, electrospray ionisation (HRMS (ESI)): M/Z calculated for $C_{13}H_2N_4$ O_4+H [M+H]: 297.1485. Found: 297.1513. Intermediate 2a was taken for next step without further characterization.

Synthesis of tert-butyl 4-(2, 4-dichloropyrimidin-5-yl)piperazine-1-carboxylate (Intermediate **2b**)

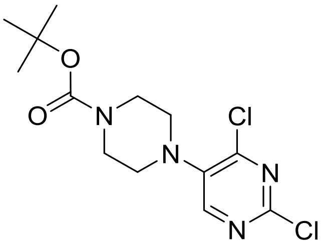

In a 2 L round-bottomed flask, (R)-tert-butyl 4-(2, 4-dioxo-1, 2, 3, 4-tetrahydropyrimidin-5-yl)-2-methylpiperazine-1-carboxylate (Intermediate **2a**, 22 g, 74.32mmol,) taken in phosphorus oxychloride (793 ml, 8506 mmol) to give a brown suspension. The reaction mixture was refluxed for 6 h. Phosphorus oxychloride was distilled out under reduced pressure; the remaining oil was diluted with THF (250 mL) and crushed ice. The reaction mixture was basified to pH 8 with Na_2CO_3. .To this was added di-tert-butyl dicarbonate (22.17 ml, 96.41 mmol, Aldrich) and stirred for 16 h at RT. The reaction mixture was diluted

with methanol and filtered it off to remove excess salt. The solvent was removed under vacuum and residue was diluted with water and extracted with ethyl acetate. The combined organic layers were dried over sodium sulphate and then removed under reduced pressure. The residue purified by flash chromatography on silica (EtoAC:hexane, 1:4) to obtain solid of tert-butyl 4-(2,4-dichloropyrimidin-5-yl)piperazine-1-carboxylate(23.00 g, 93 %). Yield: 93%, Purity: >95% by HPLC (UV at 220 and 254 nm), high-resolution mass spectrometry, electrospray ionisation (HRMS (ESI)): M/Z calculated for $C_{13}H_{18}$ $Cl_2N_4O_2$+H [M+H]: 333.0807. Found: 333.0790. Intermediate **2b** was taken for next step without further characterization.

Synthesis of tert-butyl 4-(2-chloro-4-(pyrrolidin-1-yl)pyrimidin-5-yl)piperazine-1-carboxylate (Intermediate **2c**)

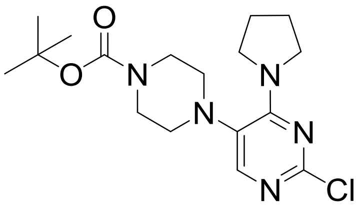

The above compound (Intermediate **2c**, 1.7 g, 95 %) was synthesized and purified as described for compound **1c** from intermediate **2b** and pyrrolidine (Sigma-Aldrich). Yield: 95%, Purity: >95% by HPLC (UV at 220 and 254 nm), high-resolution mass spectrometry, electrospray ionisation (HRMS (ESI)): M/Z calculated for $C_{17}H_{26}$ ClN_5O_2+H [M+H]: 368.1775. Found: 367.1745. Intermediate **2c** was taken for next step without further characterization.

Synthesis of compound tert-butyl 4-(2-((6-ethoxypyridin-3-yl)amino)-4-(pyrrolidin-1-yl)pyrimidin-5-yl)piperazine-1-carboxylate (Intermediate **2d**)

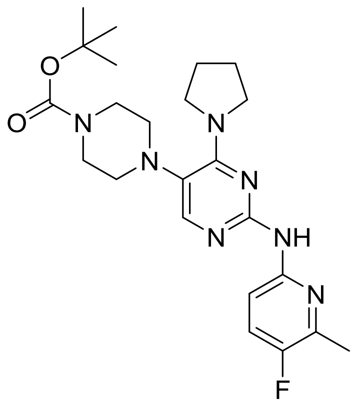

To tert-butyl 4-(2-chloro-4-(pyrrolidin-1-yl)pyrimidin-5-yl)piperazine-1-carboxylate (Intermediate **2c**, 1 g, 2.71 mmol) dissolved in Toluene(40 mL), was added 2-Amino-5-fluoro-6-methylpyridine (0.513 g, 4.07 mmol), potassium tert-butoxide(0.46 g, 4.076m mol) and degassed for 10 min. BINAP (0.010 g, 0.16 mmol), $Pd_2(dba)_3$(0.004g, 0.00543 mol) were added and again degassed for 10 minutes. The reaction was then refluxed at 110 °C for 12 h. The combined organic layer was washed with brine, dried over sodium sulphate and concentrated in vacuo. It was purified by flash chromatography on neutral alumina with gradient elution of 0.5 – 1.0% methanol in dichloromethane to get tert-butyl 4-(2-((6-ethoxypyridin-3-yl)amino)-4-(pyrrolidin-1-yl)pyrimidin-5-yl)piperazine-1-carboxylateas off white solid (0.600g, 51%). Yield: 95%, Purity: >95% by HPLC (UV at 220 and 254 nm).

^{1}H NMR ($CDCl_3$, 400 MHz) δ 8.18-8.14 (m, 1H), 7.82 (s, 1H),7.58 (s, 1H), 7.31-7.25 (m, 1H), 4.01-3.95 (m, 2H),3.75-3.64 (m, 4H),2.89-2.77 (m, 6H), 2.41-2.40 (s, 3H),1.95-1.91 (m, 4H),1.48 (s, 9H), high-resolution mass spectrometry, electrospray ionisation (HRMS (ESI)):

M/Z calculated for $C_{23}H_{32}FN_7O_2$+H [M+H]: 458.2602. Found: 458.2631. Intermediate **2d** is taken for next step without further characterization.

Synthesis of N-(6-ethoxypyridin-3-yl)-5-(piperazin-1-yl)-4-(pyrrolidin-1-yl)pyrimidin-2-amine (**3**)

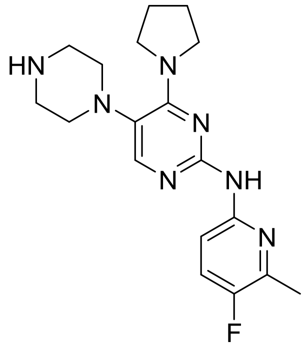

Tert-butyl 4-(2-((6-ethoxypyridin-3-yl)amino)-4-(pyrrolidin-1-yl)pyrimidin-5-yl)piperazine-1-carboxylate (intermediate **2d**, 300 mg, 0.94637 mmol) was taken in dry dichlromethane and added dioxane in HCl (2 mL) at 0°C. The reaction was warmed to 25°C and stirred for 16 h. The solvent was distilled under reduced pressure and the obtained solid was filtered and washed with dichloromethane and concentrated to obtain N-(6-ethoxypyridin-3-yl)-5-(piperazin-1-yl)-4-(pyrrolidin-1-yl)pyrimidin-2-amine white solid (220 mg, 65.47%) as a HCl salt. Yield: 95%, Purity: >95% by HPLC (UV at 220 and 254 nm).^{1}H NMR (400 MHz, DMSO-d_6) δ 13.4 (s, NH), 11.3 (s, 1H), 9.6 (s, 2H), 8.02 (s, 1H), 7.81-7.77 (m, 1H), 7.27 (d, J = 6.6 Hz, 1H), 4.06-4.04 (m, 2H), 3.68-3.66 (m, 2H), 3.31-3.29 (m, 2H), 3.16-3.13 (m, 4H), 3.02-2.9 (m, 2H), 2.54-2.45 (m, 3H), 1.95-1.89 (m, 4H) high-resolution mass spectrometry,

electrospray ionisation (HRMS (ESI)): M/Z calculated for $C_{18}H_{24}FN_7$+H [M+H]: 358.21496. Found: 358.21484.

Synthesis of (R)-tert-butyl 4-(2-chloro-4-(isopropylamino)pyrimidin-5-yl)-2-methylpiperazine-1-carboxylate (Intermediate **3a**)

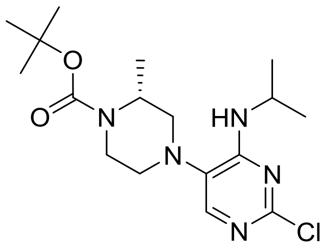

Intermediate **3a** (900 mg, 84 %) was synthesized and purified analogues procedure described for synthesis of for compound **1c** using compound **III** and isopropyl amine (Sigma-Aldrich). Yield: 84%, Purity: >95% by HPLC (UV at 220 and 254 nm), high-resolution mass spectrometry, electrospray ionisation (HRMS (ESI)): M/Z calculated for $C_{17}H_{28}ClN_5O_2$+H [M+H]: 370.1932. Found: 370.1915. Intermediate **3a** was taken for next step without further characterization.

Synthesis of (R)-N2-(5-fluoro-6-methylpyridin-2-yl)-N4-isopropyl-5-(3-methylpiperazin-1-yl)pyrimidine-2,4-diamine (**4**)

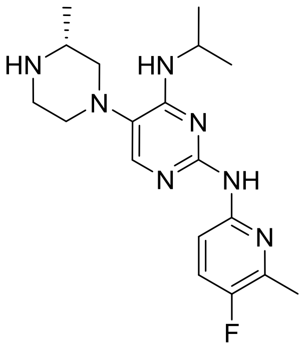

Compound **4** was synthesized suing procedures analogues to compound 7 (described in the main manuscript) using intermediate **3a** and 2-amino-5-fluoro-6-methylpyridine (Sigma-Aldrich). Yield: 63.8 % (90mg), Purity: >95% by HPLC (UV at 220 and 254 nm).

^{1}H NMR (300 MHz, DMSO-d_6) δ 13.55 (s, 1H), 11.44 (s, 1H), 9.96 (s, 1H), 9.31 (s, 1H), 8.12 (d, *J*=8.7 Hz, 1H), 7.93 (s, 1H), 7.81 (t, *J*=9.0 Hz, 1H), 7.20 (d, *J*=8.7 Hz, 1H), 4.36-4.58 (m, 1H), 3.36 (d, *J*=14.1 Hz, 3H) 3.00-3.14 (m, 2H), 2.92 (s, 1H), 2.80 (d, *J*=9.6 Hz, 1H), 2.54 (s, 3H), 1.28 (d, *J*=6.0 Hz, 9H). ^{13}C-NMR (126 MHz, DMSO-d_6) δ 157.69, 154.21, 152.58, 149.89, 147.73, 142.87, 142.74, 126.52, 125.00, 112.07, 53.89, 50.02, 47.39, 42.15, 40.06, 22.18, 17.08 and 15.45, high-resolution mass spectrometry, electrospray ionisation (HRMS (ESI)): M/Z calculated for $C_{18}H_{26}FN_7$ +H [M+H]:360.2306. Found: 360.2309

Synthesis of 6-Methyl-5-(2, 2, 2-trifluoroethoxy)pyridin-2-amine (Intermediate **4a**)

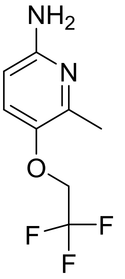

To a well stirred solution of 2, 2, 2-trifluoroethanol (0.64 g, 6.36 mmol) in dry DMF (20 mL), sodium hydride (2.6g, 6.96 mmol, 60% in mineral oil) was added at 0°C. After 10 minutes of stirring, the 3-chloro-2-methyl-6-nitropyridin (1 g, 5.8 mmol) was added and stirred at RT for 10 minutes. Then, reaction mixture was heated at 60°C for 5h. Upon completion of reaction, the reaction mixture was extracted with ethyl acetate. The organic layer was washed with water, brine solution, dried over sodium sulphate and concentrated under *vacuo*. The resultant crude was mixed with methanol (30 ml) and added 10% Pd/C (100 mg). The reaction mixture was stirred under hydrogen at RT for 16 h. The reaction was filtered through a pad of celite and washed with methanol. The filtrate was concentrated under vacuum and purified by column chromatography to get 6-methyl-5-(2, 2, 2-trifluoroethoxy)pyridin-2-amine (910 mg, 74%) as a colourless solid. Yield: 74%, Purity: >95% by HPLC (UV at 220 and 254 nm), high-resolution mass spectrometry, electrospray ionisation (HRMS (ESI)): M/Z calculated for $C_8H_9F_3N_2O$+H [M+H]: 207.0667. Found: 207.0705. Intermediate **4a** was taken for next step without further characterization.

Synthesis of (R)-N4-Isopropyl-N2-(6-methyl-5-(2, 2, 2-trifluoroethoxy)pyridin-2-yl)-5-(3-methylpiperazin-1-yl)pyrimidine-2,4-diamine (**5**)

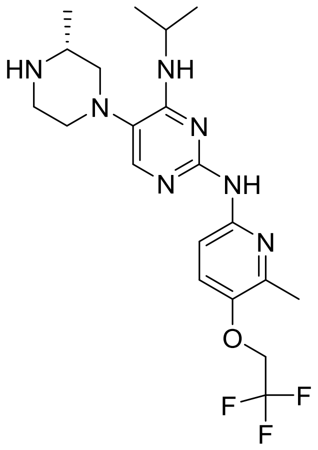

Compound **5** was synthesized as hydrochloride salt using procedures analogues to compound **7**(described in the main manuscript) using intermediate 3a and 4a .Yield: 61 % (140mg), Purity: >95% by HPLC (UV at 220 and 254 nm). ^{1}H NMR (300 MHz, DMSO-d_6) δ 13.70 (s, 1H), 11.33 (s, 1H), 9.94 (s, 1H) 9.31 (d, *J*=8.1 Hz, 1H) 8.08 (d, *J*=8.5 Hz, 1H) 7.93 (s, 1H) 7.71 (d, *J*=9.0 Hz, 1H) 7.17 (d, *J*=9.3 Hz, 1H) 4.83 (q, *J*=8.9 Hz, 4H) 4.47 (d, *J*=7.7 Hz, 1H) 3.39-3.29 (m, 2H) 3.06 (t, *J*=9.0 Hz, 2H) 2.83-2.69 (m, 1H) 1.28 (d, *J*=6.6 Hz, 9H). ^{13}C-NMR (126 MHz, DMSO-d_6) δ 157.74, 149.52, 147.65, 145.78, 145.16, 131.03, 124.99, 122.77, 111.33, 65.91, 65.64, 53.89, 47.41, 42.96, 40.01, 21.47, 18.25 and 15.47, high-resolution

mass spectrometry, electrospray ionisation (HRMS (ESI)): M/Z calculated for $C_{18}H_{26}FN_7$ +H [M+H]:440.2379. Found: 440.2381.

Synthesis of tert-butyl (R)-4-(2-chloro-4-(methylamino) pyrimidin-5-yl)-2-methylpiperazine-1-carboxylatecarboxylate (Intermediate **5a**)

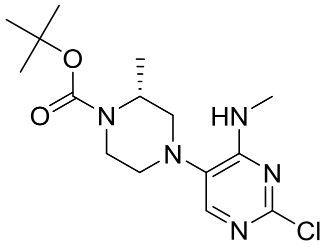

Intermediate **5a** (5.2 mg, 40 %) was synthesized and purified analogues procedure described for synthesis of compound **1c** using compound **III** and methylamine (Sigma-Aldrich). Yield: 40%, Purity: >95% by HPLC (UV at 220 and 254 nm). ^{1}H NMR (400 MHz, DMSO-d_6) δ 7.7 (s, 1H), 7.01 (s, NH),4.21 (s, 1H), 3.71-3.67 (m, 1H), 3.39-3.34 (m, 1H), 3.02-2.86 (m, 2H),2.75-2.72 (m, 3H), 2.49 (s, 1H), 2.31-2.24 (m, 1H), 1.46 (s, 9H),1.25(d, *J* = 6.6 Hz, 3H), high-resolution mass spectrometry, electrospray ionisation (HRMS (ESI)): M/Z calculated for $C_{15}H_{24}ClN_5O_2$+H [M+H]: 341.1619. Found: 341.1587. Intermediate **5a** was taken for next step without further characterization.

Synthesis of (R)-N4-Methyl-N2-(6-methyl-5-(2, 2, 2-trifluoroethoxy)pyridin-2-yl)-5-(3-methylpiperazin-1-yl)pyrimidine-2,4-diamine (**6**)

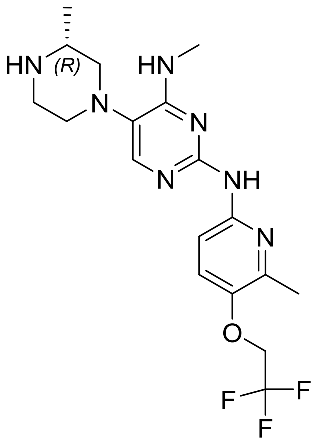

Compound **6** was synthesized as hydrochloride salt using procedure analogues to compound **7** (described in the main manuscript) using intermediate **4a** and **5a** .Yield: 77 % (57mg), Purity: >95% by HPLC (UV at 220 and 254 nm).^{1}H NMR (400 MHz, DMSO-d_6): δ 11.3 (s, 1H), 9.93 (s, 1H),9.48 (s, NH), 8.57-8.56 (m, 1H),7.93 (s, 1H),7.71 (d, *J* = 8 Hz, 1H),7.14 (d, *J* = 8 Hz, 1H),4.85-4.79 (m, 2H),3.56 (s, 1H), 3.37-3.31 (m, 2H), 3.12-3.00 (m, 4H),2.80-2.77 (m, 1H), 2.50-2.49 (m, 6H)1.27 (d, *J* = 8 Hz, 3 H). ^{13}C-NMR (126 MHz, DMSO-d_6) δ 159.13, 149.57, 147.66, 145.77, 145.17, 130.43, 127.62, 126.61, 125.13, 124.76, 122.92, 112.82, 66.15, 65.70, 62.76, 53.86, 50.28, 47.31, 42.32, 40.05, 28.04, 18.20 and 15.39, high-resolution mass spectrometry, electrospray ionisation (HRMS (ESI)): M/Z calculated for $C_{18}H_{24}F_3N_7O$ +H [M+H]:412. 1944. Found: 412.2064.

Synthesis of tert-butyl 1-(2, 4-dioxo-1, 2, 3, 4-tetrahydropyrimidin-5-yl)piperidin-4-ylcarbamate (Intermediate **10a**)

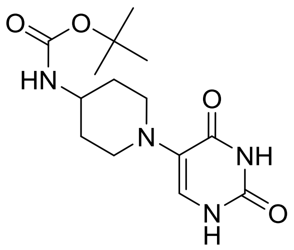

Intermediate **10a** (5.20 g, 97 %) was synthesized and purified using procedure analogues to compound 1a. Yield: 97%, Purity: >95% by HPLC (UV at 220 and 254 nm), high-resolution mass spectrometry, electrospray ionisation (HRMS (ESI)): M/Z calculated for $C_{14}H_{22}N_4$ O_4+H [M+H]: 311.1641. Found: 311.1625. Intermediate **10a** was taken for next step without further characterization

Synthesis of tert-butyl 1-(2, 4-dichloropyrimidin-5-yl)piperidin-4-ylcarbamate (Intermediate **10b**)

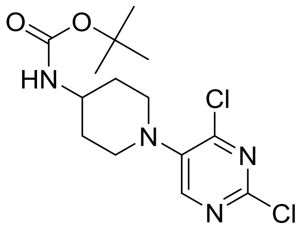

Intermediate 10b (0.84 g, 23.91 %) was synthesized and purified using procedure analogues to compound 2b. Yield: 23.91%, Purity: >95% by HPLC (UV at 220 and 254 nm), high-resolution mass spectrometry, electrospray ionisation (HRMS (ESI)): M/Z calculated for

$C_{14}H_{22}N_4O_4$+H [M+H]: 347.0963. Found: 347.0935. Intermediate **10b** was taken for next step without further characterization

Synthesis of tert-butyl (1-(2-chloro-4-((1, 5-dimethyl-1H-pyrazol-3-yl)amino)pyrimidin-5-yl)piperidin-4-yl)carbamate (**10c**)

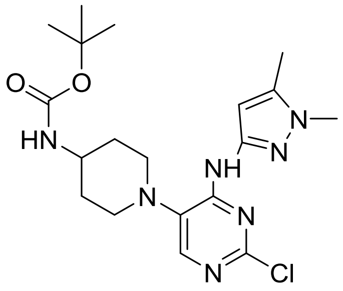

Intermediate 10c (210 mg, 34.6 %) was synthesized and purified using procedure analogues to compound **2c**. Yield: 34.6%, Purity: >95% by HPLC (UV at 220 and 254 nm), high-resolution mass spectrometry, electrospray ionisation (HRMS (ESI)): M/Z calculated for $C_{19}H_{28}ClN_7O_2$+H [M+H]: 422.1993. Found: 422.1964. Intermediate **10c** was taken for next step without further characterization.

Synthesis of 5-(4-aminopiperidin-1-yl)-N2-(4-cyclopropyl-5-fluoro-6-methylpyridin-2-yl)-N4-(1,5-dimethyl-1H-pyrazol-3-yl)pyrimidine-2,4-diamine (**10**)

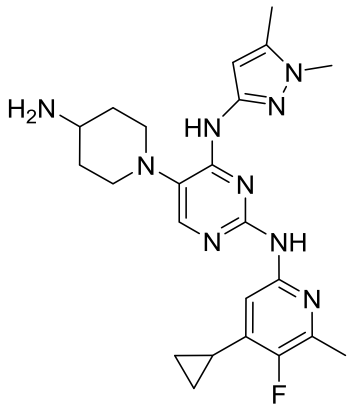

Compound **10** was synthesized as hydrochloride salt using procedure analogues to compound 7 (described in the main manuscript) using intermediate **10c** and **9a** .Yield: 48.8 % (45mg), Purity: >95% by HPLC (UV at 220 and 254 nm). ^{1}H NMR (300 MHz, DMSO-d_6) δ13.81 (s, 1H), 11.32 (s, 1H), 9.63 (s, 1H), 8.30 (s, 3H), 8.04 (s, 1H), 6.76 (d, *J*=4.5 Hz, 1H), 6.70 (s, 1H), 3.72 (s, 3H), 3.13-3.09 (m, 3H), 2.82-2.63 (m, 2H), 2.54 (d, *J*=3.2 Hz, 3H), 2.31 (s, 3H), 2.18-2.07 (m, 1H), 2.07-1.96 (m, 2H) 1.88 (d, *J*=8.9 Hz, 2H) 1.18 (d, *J*=8.5 Hz, 2H) 0.78 (d, *J*=6.4 Hz, 2H). ^{13}C-NMR (126 MHz, DMO-d_6) δ 156.93, 153.97, 152.02, 149.26, 147.43, 143.37, 141.98, 139.41, 132.66, 126.49, 107.09, 99.08, 49.79, 49.24, 46.87, 35.65, 29.58. 16.96, 10.91, 9.71 and 8.39, high-resolution mass spectrometry, electrospray ionisation (HRMS (ESI)): M/Z calculated for $C_{23}H_{30}FN_9$ +H [M+H]: 452.2608. Found: 452. 2680.

Synthesis of tert-butyl 4-(2, 4-dioxo-1,2,3,4-tetrahydropyrimidin-5-ylamino)piperidine-1-carboxylate (Intermediate **11a**)

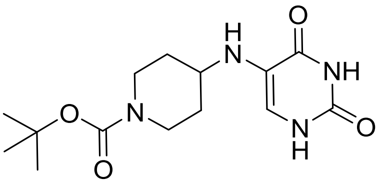

Intermediate **11a** (11,5g, 47.2 %) was synthesized and purified using procedure analogues to compound 1a using N-Boc-4-aminopiperidine and bromouracil. Yield: 47.2 %, Purity: >95% by HPLC (UV at 220 and 254 nm). ^{1}H NMR (300 MHz, DMSO-d_6) δ 11.16 (s, 1H), 10.23 (s, 1H), 6.47 (s, 1H), 4.02 (d, *J*=8.8 Hz, 1H), 3.85 (d, *J*=12.8 Hz, 2H), 3.07 (s, 1H), 2.80 (s, 2H), 1.80 (d, *J*=11.1 Hz, 2H), 1.19 (d, *J*=8.7 Hz, 2H), 1.39 (s, 9H), high-resolution mass spectrometry, electrospray ionisation (HRMS (ESI)): M/Z calculated for $C_{14}H_{22}N_4O_4$+H [M+H]: 311.1641. Found: 311.1644. Intermediate **11a** was taken for next step without further characterization

Synthesis of tert-butyl 4-((2,4-dichloropyrimidin-5-yl)amino)piperidine-1-carboxylate (Intermediate **11b**)

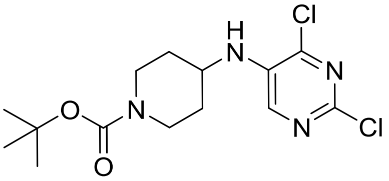

Intermediate **11b** (1.2 g, 26.8 %) was synthesized and purified using procedure analogues to compound 2b. Yield: 26.8%, Purity: >95% by HPLC (UV at 220 and 254 nm), high-resolution mass spectrometry, electrospray ionisation (HRMS (ESI)): M/Z calculated for

$C_{14}H_{22}N_4O_4$+H [M+H]: 347.0963. Found: 347.0956. Intermediate **11b** was taken for next step without further characterization.

Synthesis of tert-butyl 4-((2-chloro-4-((1, 5-dimethyl-1H-pyrazol-3-yl)amino)pyrimidin-5-yl)amino)piperidine-1-carboxylate (Intermediate **11c**)

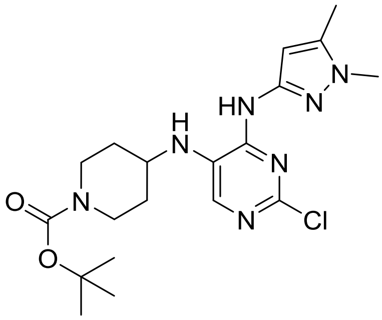

Intermediate **11c** (140 mg, 19.53 %) was synthesized and purified using procedure analogues to compound **2c**. Yield: 19.53%, Purity: >95% by HPLC (UV at 220 and 254 nm), high-resolution mass spectrometry, electrospray ionisation (HRMS (ESI)): M/Z calculated for $C_{19}H_{28}ClN_7O_2$+H [M+H]: 422.1993. Found: 422.1963. Intermediate 11c was taken for next step without further characterization**.**

Synthesis of tert-butyl 4-((2-((4-cyclopropyl-5-fluoro-6-methylpyridin-2-yl)amino)-4-((1,5-dimethyl-1H-pyrazol-3-yl)amino)pyrimidin-5-yl)amino)piperidine-1-carboxylate (Intermediate **11d**)

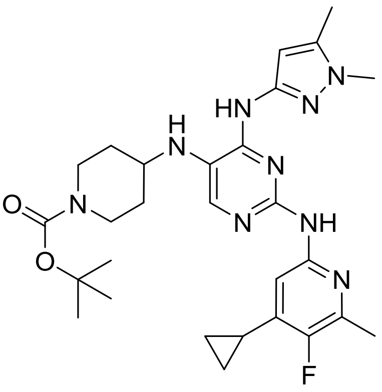

In a 100 ml RBF tert-butyl 4-((2-chloro-4-((1,5-dimethyl-1H-pyrazol-3-yl)amino)pyrimidin-5-yl)amino)piperidine-1-carboxylate (Intermediate **11c**, 300 mg, 0.71 mmol), 4-cyclopropyl-5-fluoro-6-methylpyridin-2-amine, HCl (Intermediate **9a**, 173 mg, 0.85 mmol), 2-(Dicyclohexylphosphino)-2',4',6'-tri-i-propyl-1,1'-biphenyl (40.7 mg, 0.09 mmol), Tris(dibenzylideneacetone)dipalladium (0) (65.1 mg, 0.07 mmol) and Sodium-t-butoxide (102 mg, 1.07 mmol) were taken in 1,4-dioxane (15 mL). The reaction mixture (RM) was then heated at 105°C for overnight under nitrogen. The reaction mixture was filtered off on a celite bed. The filterate was then concentrated and purified by column chromatography on silica gel (EtoAC:Hexane 4:1) to get tert-butyl 4-((2-((4-cyclopropyl-5-fluoro-6-methylpyridin-2-yl)amino)-4-((1,5-dimethyl-1H-pyrazol-3-yl)amino)pyrimidin-5-yl)amino)piperidine-1-carboxylate (110 mg, 28.0 %). Yield: 28.0 %, Purity: >95% by HPLC (UV at 220 and 254 nm), high-resolution mass spectrometry, electrospray ionisation (HRMS (ESI)): M/Z calculated for $C_{19}H_{28}ClN_7O_2$+H [M+H]: 552.3132. Found: 552.3165. Intermediate **11d** was taken for next step without further characterization.

Synthesis of N2-(4-cyclopropyl-5-fluoro-6-methylpyridin-2-yl)-N4-(1, 5-dimethyl-1H-pyrazol-3-yl)-N5-(1-methylpiperidin-4-yl)pyrimidine-2,4,5-triamine (**11**)

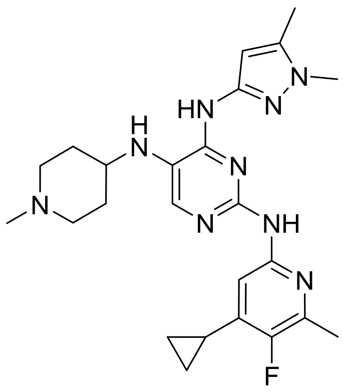

In a 10 ml RBF, tert-butyl 4-((2-((4-cyclopropyl-5-fluoro-6-methylpyridin-2-yl)amino)-4-((1,5-dimethyl-1H-pyrazol-3-yl)amino)pyrimidin-5-yl)amino)piperidine-1-carboxylate (Intermediate 11d, 80 mg, 0.15 mmol) was taken in 1,4-dioxane (2 mL). To this HCl (4N in Dioxane) (400 µL, 1.60 mmol) was added. The reaction mixture was then stirred at RT for 0.5 h. The solvent was evaporated and the residue was diluted with dichloromethane(5 mL). Triethylamine (1 mL) was added and then evaporated to dryness. To the resultant crude, ethanol (2 ml), formaldehyde (19.61 µL, 0.29 mmol) were added and the reaction mixture was stirred at room temperature (RT) for 15 mins. Then sodium cyanoborohydride (9.11 mg, 0.15 mmol) was added to this mixture. After stirring at RT for 15 mins, the reaction mixture was evaporated to dryness and the residue was purified by reverse phase chromatography to get N2-(4-cyclopropyl-5-fluoro-6-methylpyridin-2-yl)-N4-(1, 5-dimethyl-1H-pyrazol-3-yl)-N5-(1-methylpiperidin-4-yl)pyrimidine-2,4,5-triamine (20.00 mg, 29.6 %). Yield: 29.6 %, Purity: >95% by HPLC (UV at 220 and 254 nm).^{1}H NMR (300 MHz, DMSO-d_6) δ 8.76 (s, 1H), 8.80 (s, 1H), 7.67 (d, *J*=4.9 Hz, 1H), 7.52 (s, 1H), 6.86 (s, 1H), 4.65 (d, *J*=7.2 Hz, 1H),

3.64 (s, 3H), 3.17 (d, *J*=5.3 Hz, 1H), 2.76 (s, 1H), 2.80 (s, 1H), 2.32 (d, *J*=3.0 Hz, 3H), 2.23 (s, 3H), 2.21 (s, 2H), 2.13-1.98 (m, 3H), 1.98-1.87 (m, 2H), 1.51-1.33 (m, 2H), 1.25 (s, 2H) 1.10-0.98 (m, 2H), 0.85 (d, *J*=7.2 Hz, 1H), 0.76-0.65 (m, 1H), ^{13}C-NMR (126 MHz, DMO-d_6) δ152.02, 150.88, 150.52, 149.57, 146.31, 141.82, 140.15, 138.27, 136.77, 123.11, 121.17, 104.94, 99.79, 54.13, 49.79, 45.97, 35.25, 31.68. 17.18, 10.90, 8.73 and 8.60, high-resolution mass spectrometry, electrospray ionisation (HRMS (ESI)): M/Z calculated for $C_{24}H_{32}FN_9$ +H [M+H]: 466.2837. Found: 466. 2837.

Supplementary Note 2:

PK-PD model for the metabolite (compound 9) alone. Pharmacokinetic data from the *Pf*/SCID model was linked to efficacy using the following equation[5].

$$\frac{dP}{dt} = \overbrace{K_{gro} * P * \left(1 - \frac{(P)}{P\max}\right)}^{\text{Growth kinetics}} - \overbrace{K_{kill} * \left(\frac{C_e^{\,H}}{C_e^{\,H} + EC_{e50}^{\,H}}\right) * P}^{\text{Killing kinetics}}$$

Where P [log (total no. of parasites / mouse)], K_{gro} (growth rate constant), P_{max} (log (maximum no. of parasites / mouse)), K_{kill} (maximum kill rate constant), H (Hill slope), EC_{50} (concentration required for achieving half maximal kill rate) and C_e (concentration of the drug in the effect compartment). Percent parasitemia values were converted into total number of parasites in mouse blood assuming 1.7 X 10^9 (9.23 $\log_{10}$) RBCs per mouse. The lower limit of quantitation for % parasitemia was 0.01% or 5.23 $\log_{10}$. Effect compartment was used for modelling the delay observed in efficacy at a lower dose.

$$\frac{dC_e}{dt} = K_{eo} * C_p - K_{eo} * C_e$$

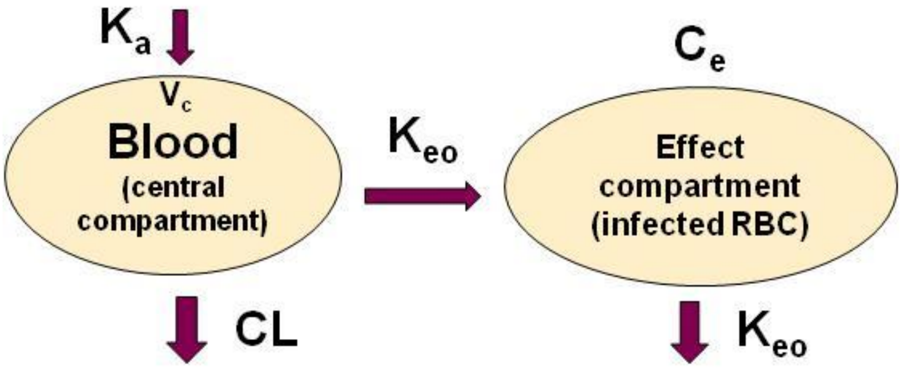

One compartment PK model was fit to the PK data obtained at 10 and 30 mg/kg, respectively (**supplementary Fig. 4 and supplementary Table 4**). All the PK-PD modeling reported in this article was performed using Phoenix WinNonlin V 6.2 and simulations were performed using Berkeley Madonna V 8.3.18. The parameters; Ka (0.3/h), CL/F (7 L/h/kg) and Vc/F (60 L/kg) were 'fixed' while modeling PK data along with the efficacy data using the PK-PD model described above (**supplementary Fig.5 and supplementary Table 5**). For the PK-PD model fit, parameters K_{e0} (0.06/h), N_{max} (12) and H (2.5) were also 'fixed'.

Supplementary Note 3: Metabolism of 12 in the presence of mouse (MLM) and human (HLM) liver microsomes.

The kinetics of metabolism of compound **12** to form the N-demethylated metabolite, **9**, was studied by incubating **12** with HLM or MLM. Compound **12** was incubated with 1 mg/mL of pooled liver microsomes at four different compound concentrations (5, 10, 20 and 30 µM) in 166 µL of buffer containing 100 mM phosphate buffer, pH 7.4 and 1 mM NADPH, at 37°C. Aliquots of 20 µL of incubation mix were quenched with 4 volumes of ice-cold acetonitrile at different time points, i.e., 0, 5, 10, 20 and 30 min in a 96 well microtitre plate. The quenched plate was centrifuged at 4000 rpm for 15 min and 20 µL of the supernatant was diluted to 200 µL with 50% acetonitrile in water. Depletion of **12** and formation of **9** was analysed by LC-MS/MS. Concentrations of **12** and **9** were determined using calibration curves prepared with synthetic standards. Kinetics of metabolite formation or parent depletion is shown in **supplementary Fig. 5, Fig. 6** and kinetic parameters are described in **supplementary Table 7**.

The rate and extent of metabolite formation in the presence of mouse liver microsomes was significantly high. The V_{max}/K_m ratio estimated in the presence of MLM was 6 times higher than in the presence of HLM.

Supplementary Note 4: Pharmacokinetic studies to estimate the blood levels of compounds in rats.

PK in Wistar rats (n = 2 per dose) was analyzed after a single dose administered intravenously (IV) or orally (PO). Blood samples were collected at 0.083, 0.25, 0.5, 1, 2, 4, 8, 23, 32, 48, 55 and 72 hours after an IV dose of 2 mg/kg or an oral dose of 10 mg/kg. Oral administration was done using oral gavage at a maximum dose volume of 10 mL/kg. Solution formulation containing 10% NMP, 20% DMA in dextrose / normal saline was used for IV administration and a suspension in 0.5% w/v HPMC with 0.1% v/v Tween 80 was used for oral administration. 25 µl of blood samples were mixed with 100 µL of Milli-Q water and vortexed for 5 minutes in the plate shaker at a speed of 900 rpm to ensure complete hemolysis. Proteins were precipitated by adding 125 µL of cold acetonotrile containing internal standard. After extraction on a plate shaker for 10 minutes at 900 rpm, the plate was centrifuged at 4000 rpm for 20 minutes. The supernatant (180 µL) was transferred to another plate and 2.5 µL of Zinc sulphate (200 mM) was added to separate the heme fraction. This mixture was centrifuged and 10 µL of the supernatant was analyzed by LC-MS/MS. Rat PK profiles are shown in **supplementary Fig. 10** and PK parameters are described in **supplementary Table 8**.

Pharmacokinetic studies to estimate the blood levels of compounds in dogs. PK in fasted, male Beagle dogs (n = 2 per dose) was studied after a single dose administered by intravenous infusion for 15 min (IV) or by an oral route (PO). Solution formulation containing 10% ethanol, 30% PEG 400, 60% saline was used for IV administration and a suspension in 0.5% w/v HPMC with 0.1% v/v Tween 80 was used for oral administration.

Blood samples were collected at 0.08, 0.25, 0.28, 0.33, 0.42, 0.75, 1.25, 3.25, 6.25, 12.25, 24.25 hours after an IV dose of 2 mg/kg and at 0, 0.25, 0.5, 1, 1.5, 2, 3, 4, 8, 12, 24, 32 and 48 hours after an oral dose of 5 mg/kg. Blood samples were centrifuged and plasma samples were analyzed by LC/MS/MS. Dog PK profiles are shown in **supplementary Fig. 11** and PK parameters are described in **supplementary Table 9**.

Supplementary Note 5: Prediction of human clearance for compound 12 by IVIVE.

Human clearance (CL) was predicted by *in vitro-in vivo* extrapolation (IVIVE) from the *in vitro* Clint in human hepatocytes[6]. *In vitro* Clint was scaled to *in vivo* Clint as follows:

$$\text{Clint}_{\text{ in vivo}} = \text{Clint}_{\text{ in vitro}} * \text{scaling factors} * \text{fu}_{\text{blood}}/\text{fu}_{\text{inc}}$$

The liver weights used for scaling were 40, 32 and 24 g/kg body weight in rat, dog and human respectively. Hepatocellularity used for scaling was 163, 169 and 120 (X 10^6) cells/g liver in rat, dog and human respectively. Free fraction in blood was calculated as fu_{plasma} /Blood:Plasma (BP) ratio. BP ratio and free fraction in the hepatocyte incubation mix, fu_{inc}, were measured *in vitro*. Slope and intercept from the linear regression analysis of IVIVC for a large number of AstraZeneca compounds was applied for final correction in the scaled *in vivo* Clint. A well stirred model was used for CL prediction.

$$\text{Predicted CL} = \frac{Q_h \times \textit{predicted}\ \text{CLint}_{\text{in vivo}}}{Q_h + \textit{predicted}\ \text{CLint}_{\text{in vivo}}}$$

Where Q_h is the liver blood flow. The values of Q_h in rat, dog and human are 72, 55 and 20 $mL.min^{-1}kg^{-1}$ in rat, dog and human, respectively. *In vitro* parameters, observed *in vivo* CL and predicted CL are shown in **supplementary Table 10**.

Prediction of human CL and V_{ss} by rat and dog allometry. Human CL for **12** was also predicted by simple, two species (rat and dog) allometry based on CL and V_{ss} estimates from rat and dog IVPK. The coefficient and exponent from allometry were used for human CL prediction by the Fu corrected intercept method (FCIM)[7]. CL was predicted as

$$\text{CL} = 33.35 \text{ x } (a/ R_{fup})\ 0.77$$

Where ‘a’ is the coefficient obtained from simple allometric scaling and R_{fup} is the ratio of free fractions in plasma, fu_p in rat to fu_p in human. V_{ss} in human was predicted by simple, rat and dog allometry (**supplementary Table 11**).

Supplementary Note 6:

Prediction of human PK profile. The predicted human CL and Vss estimates could be used for predicting human PK with a one compartment distribution. However, rat and dog IVPK profiles indicated a two compartment distribution. Parameters for the two compartment distribution in human were predicted by Wajima transformation[8] of rat and dog IVPK profiles. Human POPK profile was simulated at a dose of 260 mg (for 70 kg adult) using CL, CLd, Vt and Vc estimates, assuming similar Ka (0.3/h) and F (0.7) as observed in rats (**supplementary Fig. 12**).

Predicted human PK parameters are shown in **supplementary Table 12**. Human CL estimates predicted by the FCIM method and IVIVE (3.8 ml/min/kg) were identical. The predicted half-life in human is 36 hours. Human efficacy was predicted after treatment of 70 kg adult with a single 260 mg dose, assuming 2% parasitemia at the onset of treatment and assuming one compartment distribution of **12** in humans (**supplementary Fig. 13**). Similar efficacy was predicted if **12** was distributed in two compartments.

Supplementary Note 7:

Anaesthetised Guinea Pig Cardiovascular toxicity study for compound 12. Group mean ±SEM data are presented in **Supplementary Fig. 16a to 16l**. Mean ± standard error plasma exposure data are illustrated in **Supplementary Fig. 16m**. The mean of the two control recording periods was used as a baseline value. Administration of vehicle produced the expected level of variability in cardiovascular parameters over the time course of this study. This was considered within acceptable ranges for vehicle data. Actual changes caused by compound **12** were derived by subtracting the effect of the time-matched vehicle data and are discussed using mean % change from baseline values. All findings reported below achieved statistical significance ($P < 0.05$), unless indicated as a trend. Isolated incidences of statistical significance, that show no relation to compound effects, were not considered of biological significance.

Blood pressure, LVP and heart rate effect: Following i.v. administration of compound **12** at 10 mg/kg, the following observations were noted:

- A 15% decrease in dP/dtmax (an index of cardiac contractility).

As the dose increased to 30 mg/kg, the following effects were observed:

- A 8% decrease in heart rate (HR),
- A 10% decrease in left ventricular systolic pressure (LVsys),
- A 27% decrease in dP/dtmax.

There was evidence of recovery for dP/dtmax during the 30 minutes wash-out period. Despite attaining statistical significance, the magnitude of the effects on dP/dtmax at

10 mg/kg and heart rate and LVsys at 30 mg/kg in this study were not considered to be of biological relevance.

The magnitude of the statistically significant change in dP/dtmax observed at 30 mg/kg may in part be driven by a degree of variability in the data obtained from some of the vehicle-only treated animals. However, a decrease in dP/dtmax was observed in all six of the compound **12**-treated animals during the 30 mg/kg infusion suggesting that exposure to the compound affected cardiac contractility at the peak exposures achieved.

ECG findings: PR interval, QRS duration and QTcB interval were considered unaffected by treatment with compound **12** following administration at 10 mg/kg.

As the dose increased to 30 mg/kg, the following effect was observed:

- A 7% increase in QTcB.

This parameter remained elevated during the wash-out period. The apparent effect on QT may have been driven by a degree of variability in the data obtained from several of the vehicle animals. A trend towards an increase in QTcB was observed in some but not all of the compound **12**-treated animals. Therefore, while a change in QT was observed at the exposures achieved in this study, it is not clear whether this change was directly attributable to compound **12**.

Plasma Exposures: Peak total plasma concentrations were 1.69 ± 0.15 and 5.05 ± 0.64 μmol/L following administration of 10 and 30 mg/kg compound **12**, respectively.

Conclusion: Compound **12** caused a decrease in the index of cardiac contractility (dP/dtmax) following dosing in the guinea pig at 30 mg/kg, which showed evidence of recovery by the end of the 30 minute wash-out period. Additionally, QTcB values were higher at 30 mg/kg when compared with time-matched controls and these values remained

elevated during the wash-out period. While a change in QT was observed at the exposures achieved in thisstudy it was not clear whether this change is directly related to compound **12**. The NOEL was considered to be 10 mg/kg.

As toxicity was observed at exposures levels required for efficacy, compound **2** did not demonstrate a safety margin in either toxicity study.

Protein binding (human free)

Compound **2**	40.17%.
Compound **12**	2.5%

Guinea Pig Study: Anaesthetised guinea pigs (n=2) were treated with compound **2** at 5 mg/kg followed by 15 mg/kg (AstraZeneca, Alderley Park, UK) using methodology based on that used to assess compound **12** at Biotrial, France. Haemodynamic and electrocardiography changes were noted at both dose levels as outlined in the summary table above. Efficacy was achieved with compound **2** in an early mouse model once exposures reached approximately 9 μM Cmax (total). Consequently, compound **2** was classified as having no therapeutic window preclinically in this model.

Rat 3 Day Toxicology Study: Male Han Wister rats (n=4) received compound **2** at dose levels of 100 or 300 mg/kg for three days (AstraZeneca, Waltham, USA).

One animal was found dead, and one considered moribund on the morning of Day 4, before necropsy. These two animals considered premature decedents as they did not complete the

scheduled study or the general condition was considered sufficient to remove from the study for welfare reasons, had necropsy been scheduled.

Supplementary References:

1. Gouet, P., Robert, X. and Courcelle, E. ESPript/ENDscript: extracting and rendering sequences and 3D information from atomic structures of proteins. *Nucl. Acids Res.* **31**:3320-3323 (2003).
2. Smilkstein, M., Sriwilaijaroen, N., Kelly, J.X., Wilairat, P., Riscoe, M. Simple and inexpensive fluorescence-based technique for high-throughput antimalarial drug screening. *Antimicrob. Agents Chemother* **48**, 1803-1806 (2004).
3. Sidhu ABS, Uhlemann A-C, Valderramos SG, Valderramos J-C, Krishna S, Fidock DA. 2006. Decreasing pfmdr1 copy number in *Plasmodium falciparum* malaria heightens susceptibility to mefloquine, lumefantrine, halofantrine, quinine, and artemisinin. J. Infect. Dis. 194:528-535.
4. Preechapornkul P, Imwong M, Chotivanich K, Pongtavornpinyo W, Dondorp AM, Day NP, White NJ, Pukrittayakamee S. 2009. *Plasmodium falciparum* pfmdr1 amplification, mefloquine resistance, and parasite fitness. Antimicrob Agents Chemother 53:1509-1515
5. Fidock, D.A., Rosenthal, P.J., Croft, S.L., Brun, R., Nwaka, S. Antimalarial drug discovery: efficacy models for compound screening. Nat Rev Drug Discov. 3, 509-520 (2004).
6. Huang, L., et al. Relationship between Passive Permeability, Efflux, and Predictability of Clearance from In Vitro Metabolic Intrinsic Clearance. *Drug Metab Dispos.* **38**, 223-231 (2010).
7. Mahmood, I. Prediction of human drug clearance from animal data: application of the rule of exponents and 'fu Corrected Intercept Method' (FCIM). *J Pharm Sci.* **95**, 1810-21 (2006).
8. Wajima, T., Yano, Y., Fukumura, K., and Oguma, T. Prediction of human

pharmacokinetic profile in animal scale up based on normalizing time course profiles. *J Pharm Sci*. **93**, 1890-1900 (2004).
